# Approach to Study pH-Dependent Protein Association Using Constant-pH Molecular Dynamics: Application to the Dimerization of β-Lactoglobulin

**DOI:** 10.1021/acs.jctc.1c01187

**Published:** 2022-02-16

**Authors:** Lucie da Rocha, António M. Baptista, Sara R. R. Campos

**Affiliations:** Instituto de Tecnologia Química e Biológica António Xavier, Universidade Nova de Lisboa, Av. da República, 2780-157 Oeiras, Portugal

## Abstract

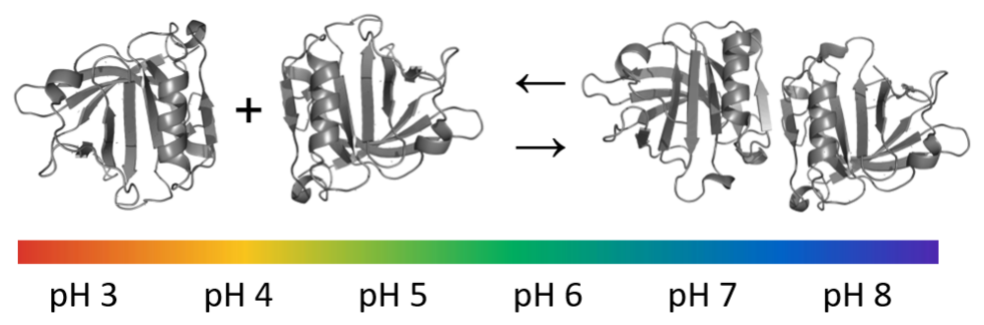

Protein–protein association is often mediated by electrostatic interactions and modulated by pH. However, experimental and computational studies have often overlooked the effect of association on the protonation state of the protein. In this work, we present a methodological approach based on constant-pH molecular dynamics (MD), which aims to provide a detailed description of a pH-dependent protein–protein association, and apply it to the dimerization of β-lactoglobulin (BLG). A selection of analyses is performed using the data generated by constant-pH MD simulations of monomeric and dimeric forms of bovine BLG, in the pH range 3–8. First, we estimate free energies of dimerization using a computationally inexpensive approach based on the Wyman–Tanford linkage theory, calculated in a new way through the use of thermodynamically based splines. The individual free energy contribution of each titratable site is also calculated, allowing for identification of relevant residues. Second, the correlations between the proton occupancies of pairs of sites are calculated (using the Pearson coefficient), and extensive networks of correlated sites are observed at acidic pH values, sometimes involving distant pairs. In general, strongly correlated sites are also slow proton exchangers and contribute significantly to the pH-dependency of the dimerization free energy. Third, we use ionic density as a fingerprint of protein charge distribution and observe electrostatic complementarity between the monomer faces that form the dimer interface, more markedly at the isoionic point (where maximum dimerization occurs) than at other pH values, which might contribute to guide the association. Finally, the pH-dependent dimerization modes are inspected using PCA, among other analyses, and two states are identified: a *relaxed state* at pH 4–8 (with the typical alignment of the crystallographic structure) and a *compact state* at pH 3–4 (with a tighter association and rotated alignment). This work shows that an approach based on constant-pH MD simulations can produce rich detailed pictures of pH-dependent protein associations, as illustrated for BLG dimerization.

## 1 Introduction

Protein–protein association occurs in various cellular processes and is an essential part of the function of many proteins. Hence, understanding and predicting the key molecular features underlying this process are fundamental. In particular, many of these associations are mediated by electrostatic interactions that are physiologically modulated by pH. Nonetheless, although protein–protein association has been widely studied by experimental and computational methods,^1−9^ the effects on protonation upon association have been often overlooked. They were addressed in some computational studies of protein–protein or ligand–protein association using either continuum electrostatics^10−18^ or constant-pH molecular dynamics (MD) simulations (CpHMD),^3,19−22^ but a detailed joint analysis of the structural and protonation features of protein–protein association was rarely pursued.

In this work, we present a methodological approach based on CpHMD that can be used to obtain a detailed description of a pH-dependent protein association and apply it to the dimerization of β-lactoglobulin (BLG). The approach consists of performing CpHMD simulations of the monomeric and dimeric forms, followed by a set of analyses aiming to integrate and synthesize the huge amount of data relating protonation and structure, monomer and dimer, and their dependence on pH. First, we estimate free energies of dimerization using a computationally inexpensive approach, in contrast with the usual free energy methods (e.g., thermodynamic integration or perturbation). The pH-dependent change of the dimerization free energy is computed solely from CpHMD simulations of the monomeric and dimeric forms, by making use of thermodynamic differential relations based on Wyman’s^23^ and Tanford’s^24^ linkage theory. Linkage relations have already been used in CpHMD studies^19−22,25−27^ but use a different approach to integrate the protonation curves (a necessary step in the calculation). In this study, a more robust procedure based on cubic Hermite splines with slopes directly derived from the simulations is used. Second, we calculate correlations between the proton occupancies of pairs of sites to identify electrostatically coupled residues possibly playing a key functional role. Since these coupled sites may restrict the protein charge distribution and lead to kinetically trapped protonation states, proton-exchange times were also investigated. Third, we analyze how protein charge distribution is affected by pH and whether dimerization is driven by charge complementarity. This is done by using the solution ionic distributions, which avoids the calculation of the protein electrostatic potential (e.g., using a Poisson–Boltzmann model) and its averaging over the sampled conformation and protonation states. Finally, we analyze pH-dependent dimerization modes, considering both the dimer tightness and the relative orientation of the two chains. Among other things, we perform principal component analysis (PCA) based on a judicious choice of coordinates, uncoupling the fitted and the PCA-transformed protein regions.

Our model system is β-lactoglobulin, a small β-barrel protein (18.3 kDa) containing 162 amino acids that has been widely studied over the past 60 years.^28−31^ It can be found in the milk of ruminant species, being a major component of bovine milk,^29^ and it is known to cause an allergic reaction in susceptible individuals.^32^ This protein belongs to the lipocalin superfamily of transporter molecules, binding small hydrophobic molecules in its hydrophobic cavity such as retinol, fatty acids, progesterone, and others. Although its function is not totally understood, it is believed to act as a specific transporter of such ligands^28,29^ and might also assist the delivery of immunoglobulins to ruminant offspring.^33^ BLG experiences a pH-dependent conformational transition, the so-called Tanford transition,^34^ that has been associated with the movement of a loop (the EF loop) located at the entrance of the hydrophobic cavity. This loop acts as a gate to the cavity, and therefore, BLG can be found in an open or in a closed conformation.^35,36^

**Figure 1:**
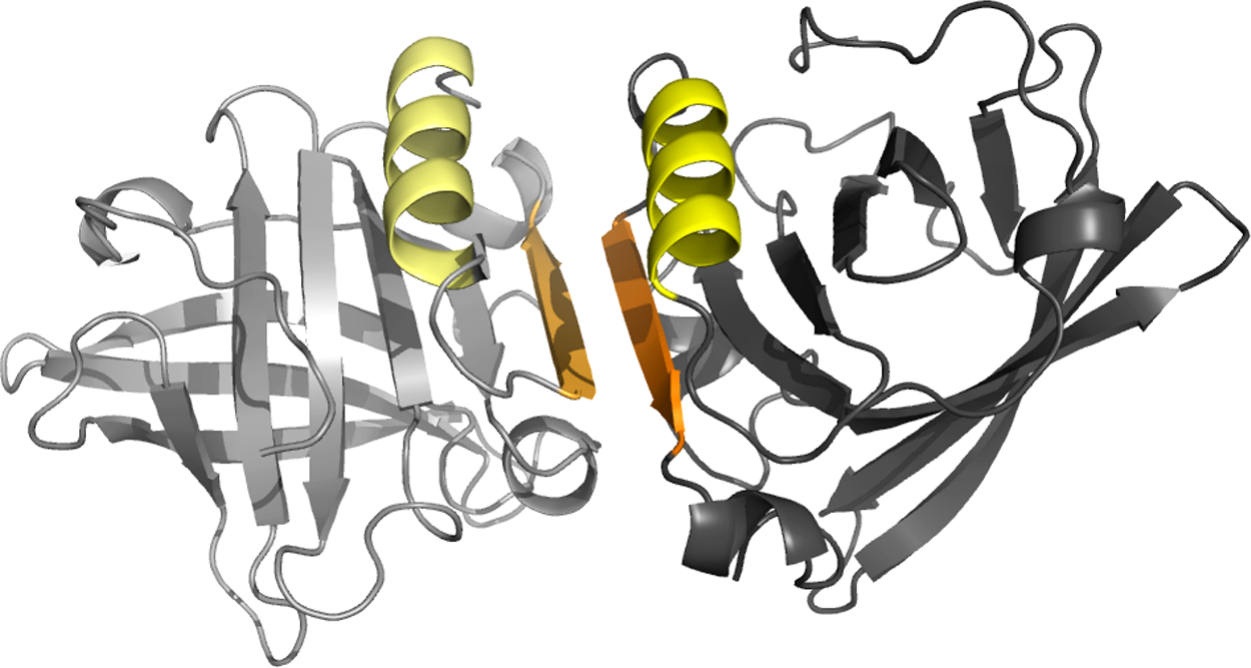
Structure of the β-lactoglobulin dimer (generated from PDB entry 1BSY(36) as described in section 2.1). The pair of helices and the pair of β-strands at the interface are highlighted in yellow and orange, respectively.

Most studies of BLG association have focused on the monomer–dimer equilibrium,^37−41^ although higher aggregates can also be found.^4,39^ These studies have been performed using essentially every available biophysical technique (SAXS, light scattering, sedimentation and diffusion, isothermal titration calorimetry, etc.), but due to differences in experimental conditions, somewhat unclear results appear when comparing the literature data (see ref (41) and references therein). The monomer–dimer equilibrium appears to be strongly pH-dependent at a certain protein concentration range, although ionic strength and temperature can also affect the equilibrium.^4,37,42−44^ In most experimental conditions tested, the monomeric form is predominant at pH below 3 and above 8, and between these there is the formation of a reversible dimer,^37,41,44−46^ with dimerization being maximized at pH values near the isoelectric point (∼5.3^47,48^) and decreasing as |pH-pI| increases.^37,41,49−52^ In particular, this was verified by Mercadante et al.^41^ who have measured the dissociation equilibrium constants for BLG at pH values 2.5, 3.5, 6.5, and 7.5 and observed a huge fraction of the dimeric form at pH 4.5 and 5.5. Moreover, as in any self-association, the fraction of the dimeric form in solution also markedly depends on the protein total concentration (see Figure S1). The structure of the dimer is shown in Figure 1, which highlights the pair of helices (yellow) and of β-strands (orange) aligned in an antiparallel orientation at the interface.

Herein, we use the stochastic titration method^53,54^ to perform CpHMD simulations of the monomeric and dimeric forms of BLG, in the pH range 3–8. We then perform a thorough analysis of the structural and protonation data using the general analysis approach described above, which yields an integrated detailed description of the pH-dependent dimerization of BLG. This work shows how a good analysis of results can greatly boost the power of CpHMD methods to study protein–protein association.

## 2 Theory and Methods

### 2.1 Structural Models

The structural models chosen for this study were the monomer and dimer of bovine β-lactoglobulin variant A. Two different conformations were chosen from the protein data bank (PDB): one corresponding to the EF loop in the open position (1BSY^36^) and the other corresponding to the closed position (3BLG^36^). These two structures correspond to the monomeric form, and their files contain the matrices for crystallographic symmetry transformations. The respective dimeric forms were created by generating the symmetry partners within 20 Å, using PyMOL,^55^ and selecting the proper partner after comparison to an available, though incomplete, dimer PDB structure.^56^

After being placed in a rhombic dodecahedron box with periodic boundary conditions, the open and closed monomers were solvated with a total of 9760 and 9445 water molecules, respectively, and the open and closed dimers were solvated with a total of 37730 and 36306 water molecules, respectively.

Preliminary simulations of the monomer and dimer without ions were used to estimate the net charge of the protein at each pH (shown in Table S1). We then computed the numbers of Na^+^ and Cl^–^ ions that would give neutralization and an ionic strength of 0.1 M (i.e., that would simultaneously satisfy the equations *Z*_protein_ + *N*_Na^+^_ – *N*_Cl^–^_ = 0 and *I* = (*N*_Na^+^_ + *N*_Cl^–^_)/(2*N*_A_*V*)), which were then rounded to the nearest integer (shown in Table S1). Solvent molecules were then randomly replaced with those Na^+^ and Cl^–^ ions, which should produce a simulation box typical of ionic strength 0.1 M that would remain approximately neutral during the simulations (see Results and Discussion).

### 2.2 Molecular Mechanics (MM) and Molecular Dynamics (MD) Settings

The GROMOS 54A7 force field^57^ and the SPC water model^58^ were used to describe the molecular system. MM/MD simulations were performed with the GROMACS package version 4.0.7,^59^ modified in-house^60^ to include ionic strength as an external parameter when using the generalized reaction field (GRF).^54^ All bonds were constrained using the LINCS algorithm^61^ for the protein and the SETTLE^62^ algorithm for the water. Nonbonded interactions were treated using a twin-range method with cutoffs 0.8 and 1.4 nm, with lists updated every 5 simulation steps. The GRF method^63^ was used for electrostatic interactions with a dielectric constant of 54^64^ and ionic strength set to 0.1 M. The temperature was coupled separately for the solute and solvent at 300 K using a v-rescale coupling bath^65^ with a relaxation time of 0.1 ps. A Parrinello–Rahman pressure coupling bath^66^ was set at 1 atm with a relaxation time of 0.5 ps. The equations of motion were integrated using a Verlet leapfrog algorithm with a time step of 2 fs.

A two-step minimization was achieved with ∼10000 steps of steepest descent with position restraints on all non-hydrogen protein atoms, with a force constant of 1000 kJ mol^–1^ nm^–2^, followed by another ∼10000 steps with no restraints. The MD relaxation phase consisted of 50 ps of NVT with all protein non-hydrogen atoms restrained, followed by 50 ps of NVT where only C^α^ atoms were restrained, and ended with 100 ps of NPT with the C^α^ restrained, using the same restraint force.

### 2.3 Constant-pH MD Simulations

Constant-pH MD simulations were performed using the stochastic titration method developed by Baptista and co-workers.^53,54^ This methodology performs an MM/MD simulation at a given pH, that is periodically interrupted to update the protonation states using Poisson–Boltzmann (PB) and Monte Carlo (MC) calculations, which results in a proper sampling of conformational and protonation states.^53^ All constant-pH simulations were performed for 100 ns at pH values 3, 4, 5, 6, 7, and 8. For the closed and open monomers, two simulation replicates were performed at each pH with different sets of random initial velocities, whereas for the closed and open dimers, four replicates were analogously done, amounting to 72 independent simulations. The structures were saved every 10 ps.

PB/MC simulations were performed every 10 ps, followed by an MD step of solvent relaxation of 0.2 ps.^53^ Proton tautomerism^67,68^ was applied for all sites considered titratable in the pH range 3–8 (Asp, Glu, His, Nter, and Cter), selected based on preliminary rigid-structure PB/MC calculations with the crystallographic structures. In particular, we note that, although a free Cys in water has a p*K*_a_ of 8.6,^69^ close to our upper bound, the only free Cys in BLG, Cys121, yielded a p*K*_a_ ≥ 15 in those preliminary calculations and remained deeply buried in its hydrophobic region during the subsequent CpHMD simulations. The reduced titration approach^54^ was used, where a site exclusion list was updated every 50 cycles of CpHMD, considering a threshold of 0.999 protonation state frequency.

The PB calculations were done by using the MEAD package (version 2.2.9)^70^ and consisted of finite difference calculations using a two-step focusing approach with grid spacings of 1.0 and 0.25 Å. The atomic charges and radii were obtained from the GROMOS 54A7 force field as explained in ref (68). The molecular surface was defined with a solvent probe of radius 1.4 Å and a Stern layer of 2.0 Å, whereas the dielectric constant was set to 2 for the molecular interior and 80 for the solvent. The temperature was set to 300 K, and the ionic strength was set to 0.1 M. The preparation of all the necessary files for the PB calculations with tautomers was made using the in-house package meadTools (version 2.2).^60,67^

The sampling of protonation states was done by using the MC method implemented in the PETIT program (version 1.6).^60,67,71^ 10^5^ MC cycles were performed, with each cycle consisting of random choices of state according to the Metropolis rule^72^ for all individual sites and for pairs of sites with a coupling above 2.0 p*K*_a_ units.^71^

The CpHMD implementation used in this work (version ST-CpHMD-v4.1_GMX4.07) relies on GROMACS (version 4.0.7)^59^ for MM/MD simulations, MEAD (version 2.2.9)^70^ for PB calculations, and PETIT (version 1.6)^67,71^ for the protonation sampling with MC. These are all available on our lab’s web site.^60^

### 2.4 Relative Dimerization Free Energy

The set of CpHMD simulations produces average protonations of the monomeric and dimeric forms at several pH values, which can be used to compute pH-dependent relative values for the dimerization free energy. In particular, we know from linkage function theory^23,24^ that for a dimerization reaction
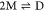
1the slope of its free energy change is given by
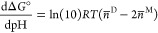
2where *n̅*^M^ and *n̅*^D^ are the average numbers of protons bound to the monomer and the dimer, respectively, at the pH value being considered. The relative dimerization free energy at a given pH is then
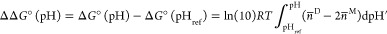
3where pH′ stands for the integration variable, and pH_ref_ is an arbitrary reference pH. Usually, pH_ref_ is assigned the lowest sampled pH (3 in the present study), but that is just a practical convenience in computing the integral. In other words, the integration actually provides only a *shape* for the Δ*G*°(pH) function.

Since each total protonation is a direct sum of individual contributions from all titratable sites, the relative free energy can be easily split into site-specific contributions,^19,22^ which makes it possible to identify which sites are most determinant for the pH sensitivity of the dimerization process. In the case studied here, where the dimer consists of two identical chains A and B, it is convenient to index each site relative to a single chain. Using this indexing, the free energy can be split as
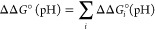
4where
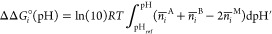
5is the contribution from site *i*, and  is the average protonation of site *i* in chain X.

The calculation of the above integrals tends to be trivial in rigid-structure PB/MC studies,^12,73,74^ since the many and closely spaced pH values that are typically sampled make it possible to get an accurate result using a rectangle or trapezium method. In contrast, CpHMD studies usually sample a small and sparse set of pH values (here six values with a relatively large spacing of 1 pH unit), which can make the calculation problematic. One solution is to fit a Hendersen–Hasselbalch or Hill curve to the computed average protonations of each individual site, which can then be integrated to an analytic expression that depends only on the fitted parameters (p*K*_a_ and Hill coefficients).^19,21,22^ However, although these fits can be a suitable way to estimate the pH value of midpoint titration, as done in the present study (see section 2.7), the fitted curve often fails to capture local features of the observed titration profile, which might look like relatively small local deviations but could lead to significant cumulative integration errors; and in some cases, these fits can be very poor (see section 3.1). The method adopted here also uses an analytical expression for the integral, but the integrand is instead derived from a Hermite cubic spline,^75^ taking advantage of the fact that we actually know the slope of each titration curve at all simulated pH values. Indeed, the slope of the total titration curve is^23,76,77^
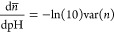
6where var(*n*) is the variance of the total number of bound protons at the considered pH value, which can be directly computed from the simulations. Similarly, as shown in the Appendix, the slope of the individual titration curve of site *i* is
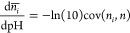
7where cov(*n*_*i*_,*n*) is the covariance between the occupancy of *i* and the total number of bound protons, a quantity that can also be directly computed from the simulations. By having the sampled average protonations and their corresponding slopes, we can then derive an interpolating Hermite cubic spline^75^ and easily compute its integral as an analytical expression (see section 2.1 in the Supporting Information). In this way, the pH-dependent ΔΔ*G*° and ΔΔ*G*_*i*_° profiles are obtained from spline interpolation curves that pass through the actual average protonations, instead of from fitted Hill curves that may deviate from them in some pH regions. In particular, these spline-derived curves do not depend on the fitting-derived p*K*_a_ and Hill coefficient values.

### 2.5 Ionic Density

The probability densities of the Na^+^ and Cl^–^ ions around the protein were calculated in a grid of mesh size 1 Å and converted to concentrations in mM. A Gaussian kernel estimator^78,79^ of bandwidth 2 Å was used to calculate the probability densities with the program LandscapeTools,^60,78^ after determining the positions of Na^+^ and Cl^–^ ions when the protein is fitted to a central structure.^78^

For comparison, an estimate of the ion concentrations was obtained by performing PB calculations and applying the equation^80,81^
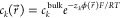
8where *c*_*k*_(*r⃗*) is the concentration of an ion of species *k* at position *r⃗*, *c*_*k*_^bulk^ is its concentration in the bulk, *z*_*k*_ is its charge (in protonic units), *F* is the Faraday constant, *R* is the gas constant, *T* is the absolute temperature, and ϕ(*r⃗*) is the electrostatic potential at position *r⃗*, calculated with the PB equation. The PB equation is usually derived for a fully static molecule, meaning that the provided charges and the computed potential should, in principle, refer to a specific protonation state and to a specific protein conformation. However, since the PB equation is linear, the potential averaged over all protonation states can be computed by simply using the corresponding averaged charges, which can be obtained from the protonation states sampled by the CpHMD simulations. The effect of the conformation variability can be approximately included by using a single representative structure. Therefore, the PB calculations were performed on a single central structure^78^ for each pH value, using average charges. The PB equation was solved by use of the MEAD program version 2.2.9^70^ and the settings described for the CpHMD simulations.

### 2.6 Conformational Characterization Using Principal Component Analysis

Principal component analysis (PCA)^82^ was used to inspect the pH-dependency of two different structural arrangements: the arrangement of the two dimer chains and the EF loop conformation. PCA operations were performed using the Python scikit-learn package,^83^ after carefully selecting the structural fitting of the input structures and the coordinates to be PCA-transformed.

In the analysis of the dimer configurations, the selected coordinates were the Cartesian coordinates of the backbone atoms (N, C^α^, C) of one chain after fitting the backbone atoms of the other chain to the crystallographic structure. This choice of coordinates intended to capture the rigid-body motion of the two dimer partners. The analysis used all frames from all equilibrated simulations at different pH values and included the coordinates extracted from each chain after fitting the respective partner. The first two principal components (PCs) captured more than 80% of the total variance.

The PCA of the EF loop was performed using the Cartesian coordinates of the backbone atoms of the loop (residues 84–90) after fitting the remaining backbone atoms to the crystallographic structure. Once again, the atoms used for the fitting and the ones used for the PCA are mutually exclusive, in order to capture the intended domain motion. All frames from all equilibrated simulations at different pH values were used in the analysis, which included the monomer and both dimer chains. The first two PCs captured more than 70% of the total variance.

For both analyses, energy landscapes in the space of the first two PCs were calculated for separate pH values, using the program LandscapeTools.^60,78^ First, the probability density was computed on a grid using a Gaussian kernel density estimator with a bandwidth of σ(4/3*N*)^1/5^, where σ is the standard deviation of the *N* sampled points.^78,79^ The grid mesh size was 2 Å in the analysis of the dimer configuration and 0.5 Å in the analysis of the EF loop. Then, the corresponding free energy surface was calculated according to
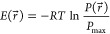
9where *r⃗* is the coordinate in the two-dimensional space, and *P*_max_ is the maximum of the probability density function, *P*(*r⃗*).

### 2.7 Other Analyses

Standard analyses were performed using the GROMACS package^59,84^ or in-house tools on the equilibrated last 70 ns of each simulation. The equilibration time was decided based on the observation of the temporal evolution of several properties: root-mean-square deviation (RMSD, Figure S2), secondary structure content (Figure S3), and protonation (Figures S6 and S7). Dissociation events were observed in some simulations, usually short-lived (see Figures S4 and S5 in the Supporting Information), in some cases with anomalous reassociation. The distance between the centers of mass (COM) of the two partners and the RMSD relative to the crystallographic structure of the dimer were measured (Figure S8), and structures with distances greater than 3.3 nm and/or RMSD greater than 1.2 nm were considered dissociated and excluded from all other analyses of the dimer simulations.

The titration curves were obtained by averaging at each pH value the occupancy states of each titratable site over the equilibrated system. A Hill curve
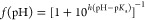
10was fitted to the average protonations to obtain the p*K*_a_ and *h* (Hill coefficient) values for each titratable site. These fits were done by using the Marquardt–Levenberg nonlinear least-squares algorithm^85^ implemented in gnuplot.^86^

The errors of all protonation-related quantities were computed using the bootstrap method described in ref (87), as explained in detail in section 2.2 of the Supporting Information.

The coupling between the protonation of pairs of titratable sites was measured using the Pearson correlation coefficient,^88^ which can detect direct interactions between pairs of proton-binding sites and also indirect effects mediated through other sites.^71^ The correlation coefficient (ρ_*ij*_) for a pair of sites *i* and *j* is determined from the covariance and variance of their proton occupancies
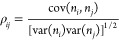
11with *n*_*i*_ and *n*_*j*_ being equal to zero when the site is empty or one when occupied. ρ_*ij*_ is comprised between −1 and +1.

The protein solvent-accessible surface area (SASA) was calculated with the GROMACS tool g_sas using a rolling probe with a radius of 0.14 nm. The contact surface area between the two dimer partners was calculated as
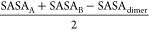
12where A and B represent each of the dimer partners alone.

Several analyses have used a *central structure*, as defined in ref (78), calculated with the in-house tool grms_to_central.^60^

Unless stated otherwise, all histograms were computed using a Gaussian kernel estimate with a bandwidth of σ(4/3*N*)^1/5^, where σ is the standard deviation of the *N* sampled points, as implemented in gnuplot,^79,86^ and normalized using 1/*N*.

All molecular representations were done by using the PyMOL software.^55^

## 3 Results and Discussion

Overall, the secondary structure of BLG is well conserved in the simulations (Figure S3). In addition, as observed in Table S2, the simulation box remains approximately neutral at all pH values, deviating on average less than one charge unit. Since the total number of added ions was computed from the average protein charge in preliminary simulations without ions (see section 2.1), the fact that the simulation box remains neutral indicates that the protein charge is not significantly affected by the presence of ions when a reaction field treatment is used for the electrostatics, as previously observed for other solvated systems.^89^ This robustness means that the discrete stochastic titration CpHMD method used here can do without the strict neutralization adopted in other methods,^90,91^ perhaps because there are no continuous dynamical titration variables which may propagate fluctuations to the structural ones.

### 3.1 Protonation Curves

**Figure 2:**
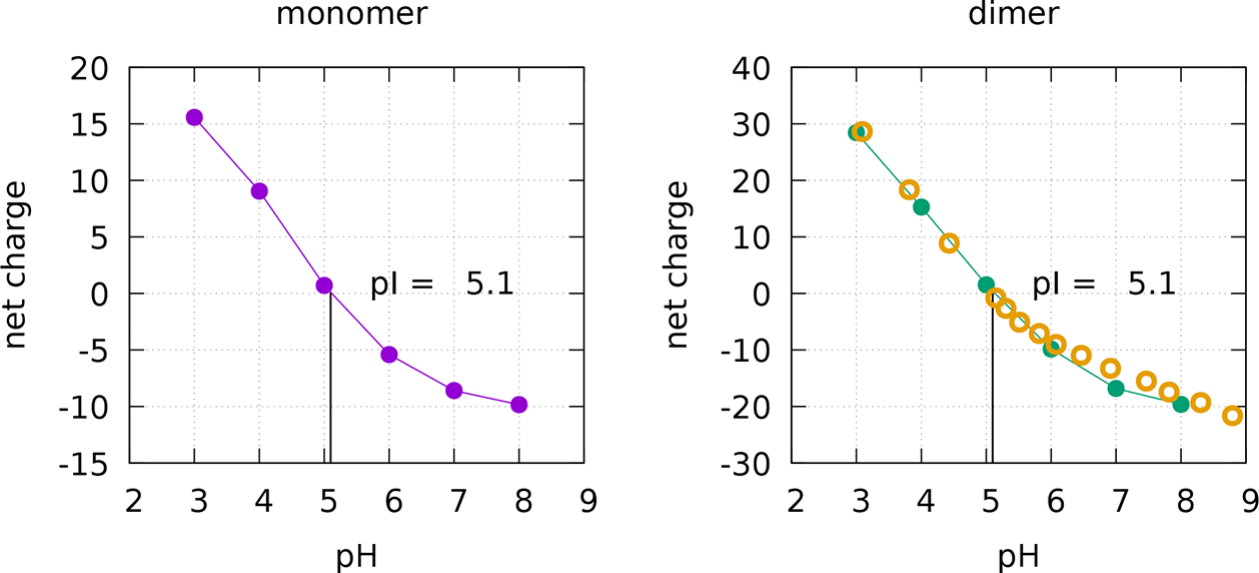
Mean protein charge as a function of pH for the monomer (purple) and dimer (green). The errors are inferior to half a charge. The pI is also shown. The empty orange circles are data points from potentiometric titration obtained by Basch and Timasheff.^48^

The global protonation curves of BLG in the monomeric and dimeric forms are shown in Figure 2. In both forms, the isoionic point (pI) is 5.1, which is in good agreement with the experimental values reported in the literature (in the range 5.2–5.4, estimated in the presence of bound cations^47,48^). A potentiometric titration curve obtained by Basch and Timasheff^48^ is also depicted in Figure 2 for the dimer, in very good agreement with the simulation results except for a small deviation at pH 7.

The protonation curves of each individual titrating site are presented in the SI (Figure S9), and the respective p*K*_a_ values are presented in Table 1 (extrapolated values lower than 3 should be regarded as indicative only). The available NMR-based p*K*_a_ values were either measured at pH below 3.3^45^ or were derived by combining experimental data with PB calculations^92^ and do not show a good agreement between them.

**Table 1:** p*K*_a_ Values in the Monomer and Dimer^a^ and the Corresponding Shift from Monomer to Dimer

site	monomer p*K*_a_	dimer p*K*_a_	Δp*K*_a_
Nter	6.9 ± 0.1	7.0 ± 0.1	0.2
Asp11	3.2 ± 0.0	3.2 ± 0.0	0.0
Asp28	3.3 ± 0.1	3.1 ± 0.1	–0.2
Asp33	4.2 ± 0.1	4.4 ± 0.2	0.2
Glu44	4.1 ± 0.1	4.2 ± 0.1	0.1
Glu45	5.5 ± 0.1	5.5 ± 0.0	0.0
Glu51	3.7 ± 0.1	3.6 ± 0.0	–0.1
Asp53	4.9 ± 0.2	4.8 ± 0.1	–0.1
Glu55	5.9 ± 0.1	6.0 ± 0.1	0.2
Glu62	4.4 ± 0.1	4.3 ± 0.0	–0.1
Asp64	4.2 ± 0.0	4.3 ± 0.0	0.1
Glu65	4.7 ± 0.0	4.7 ± 0.0	–0.0
Glu74	4.9 ± 0.1	4.8 ± 0.1	–0.0
Asp85	4.1 ± 0.1	3.9 ± 0.1	–0.2
Glu89	4.4 ± 0.2	4.7 ± 0.2	0.4
Asp96	2.7 ± 0.2	3.0 ± 0.1	0.2
Asp98	2.4 ± 1.7	0.9 ± 0.5	–1.4
Glu108	5.5 ± 0.1	5.1 ± 0.1	–0.4
Glu112	3.7 ± 0.3	3.9 ± 0.1	0.2
Glu114	4.3 ± 0.1	4.3 ± 0.1	0.0
Glu127	4.3 ± 0.0	4.1 ± 0.1	–0.2
Asp129	3.8 ± 0.1	3.4 ± 0.1	–0.4
Asp130	3.9 ± 0.0	3.1 ± 0.2	–0.8
Glu131	4.1 ± 0.1	3.9 ± 0.0	–0.1
Glu134	4.7 ± 0.0	4.6 ± 0.1	–0.1
Asp137	3.4 ± 0.1	1.8 ± 0.8	–1.6
His146	6.7 ± 0.1	6.0 ± 0.1	–0.6
Glu157	5.3 ± 0.0	5.4 ± 0.0	0.1
Glu158	5.4 ± 0.0	5.6 ± 0.0	0.2
His161	4.3 ± 0.7	1.6 ± 16.4	–2.8
Cter162	4.8 ± 0.2	4.7 ± 0.2	–0.1

a With bootstrap errors, computed as described in section 2.2 of the Supporting Information.

**Figure 3:**
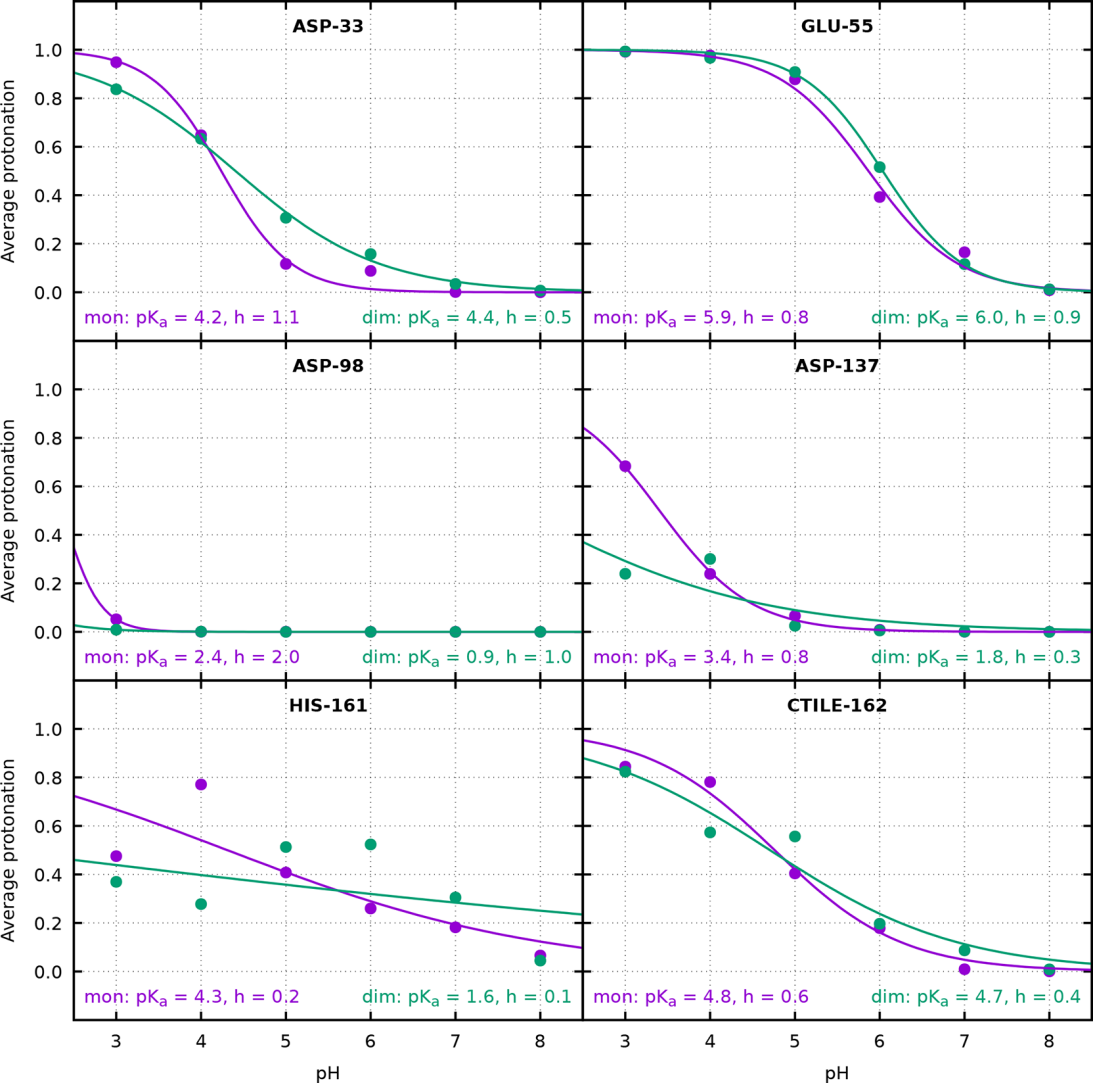
Average protonation of selected sites in the monomer (purple) and dimer (green), showing the p*K*_a_ values and Hill coefficients *h* obtained from the fit of a Hill curve. See Figure S9 for errors.

Figure 3 presents some sites with interesting or unusual protonation curves. Asp33 experiences a substantial change in the shape of its protonation curve upon dimerization, despite a very small p*K*_a_ shift, illustrating the fact that a p*K*_a_ value alone does not fully describe the protonation behavior. A p*K*_a_ higher than usual is observed for the solvent-exposed Glu55, possibly due to the concerted effect of its several charged or titratable neighbors. On the other hand, Asp98 presents an unusually low p*K*_a_, which can be related to the positively charged environment that surrounds it, namely the proximity of lysines. The effect of dimerization is strongly felt by Asp137, which becomes partially buried at the interface where it establishes salt bridges with the several Arg and Lys groups in its proximity; this affects both the shape and the midpoint of the protonation curve, with a significant decrease in the p*K*_a_. The very atypical protonation curve of His161 is the most striking example, particularly in the dimer, and the p*K*_a_ values estimated from fitting a Hill curve should be regarded as indicative only. This residue is near the dimer interface and in both the dimer and monomer is internalized in a hydrophobic area close to three phenolic rings, which results in a transient kinetic trapping of the protonation states (see section 3.3), leading to the observed dispersion of points and large errors (shown in Figure S9). On the other hand, the neighbor C-terminus in Ile162 is solvent-exposed and presents a smaller deviation from a Hill curve.

### 3.2 Dimerization Free Energy

**Figure 4:**
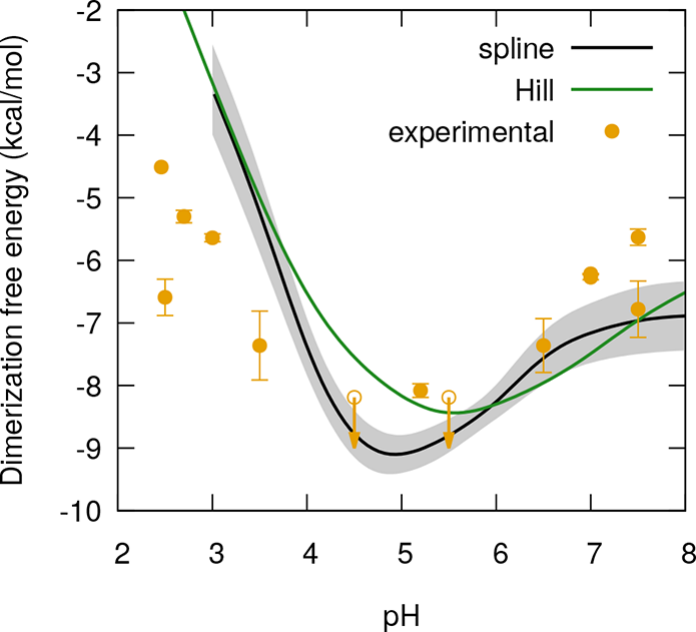
Dimerization free energy as a function of pH, calculated with the splines-based (black line) and the Hill-based (green line) methods. The gray shadow area delimits the error bounds obtained using a bootstrap method, as explained in section 2.4. The yellow dots (with error bars) correspond to experimental points with a temperature of 20 °C^37,52,93^ or 25 °C^41,49−51^ (see Table S3 for details). The dots with downward arrows are upperbounds.

**Figure 5:**
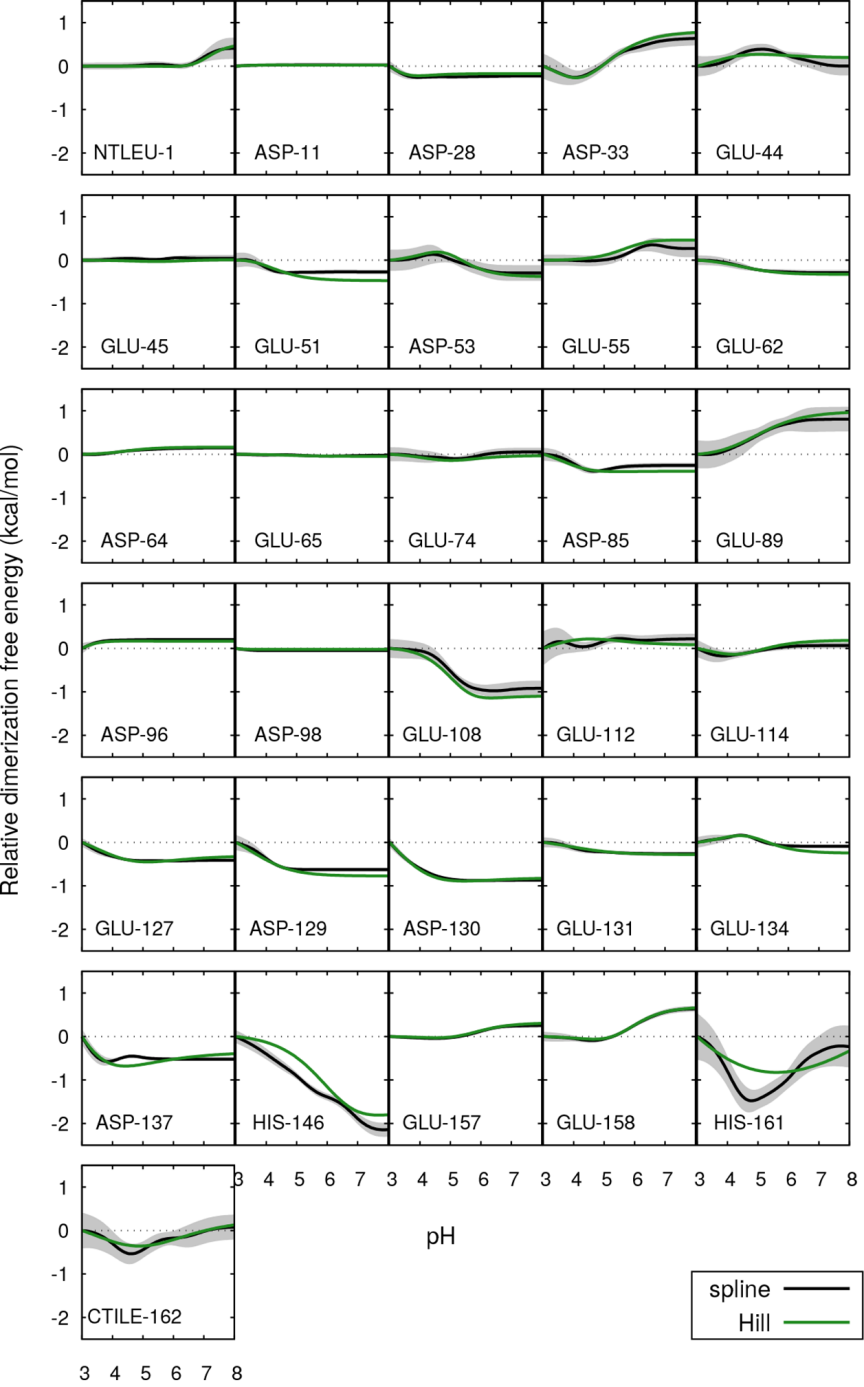
Site-specific contributions for the dimerization free energy relative to pH 3, as a function of pH. For further details, see the caption of Figure 4.

As explained in section 2.4, the protonation curves of the monomeric and dimeric forms of BLG can be used to compute pH-dependent relative values for the dimerization free energy, by applying linkage function theory.^23,24^ This approach requires a sound method to calculate the integrals of the protonation curves obtained with CpHMD, which typically contain a small and sparse set of pH values. Here, we use a new spline-based integration method and compare it with the more approximate Hill-based integration method (see section 2.4 for details). Both approaches are used to calculate the dimerization free energy of BLG (Figure 4) and the individual contributions from all titratable sites to the relative dimerization free energy (Figure 5). There are several reported dimerization constants that can be compared with our results (see Table S3 in the Supporting Information) and were included in Figure 4. Since, as discussed in the end of section 2.4, the calculation of the integral in eq 3 produces only a curve shape (corresponding to a relative free energy, ΔΔ*G*°(pH)), the computed spline-based and Hill-based curves were each vertically least-squares fitted to the experimental data shown in the figure (producing an absolute free energy curve, Δ*G*°(pH)). The individual contributions shown in Figure 5 pertain to the relative free energies using pH 3 as reference.

The dimerization free energy curves (Figure 4) obtained with the two alternative integration methods present different shapes. The pH of maximum dimerization differs by almost a pH unit, and a plateau at pH 7–8 is only observed with the spline-based calculation. Unsurprisingly, the two methods give equivalent or very similar results for the free energy contributions of most individual sites, whose protonation is indeed well expressed by a Hill curve. The differences arise from those sites that either present small deviations from a Hill curve that end up accumulating in the calculation of the integral (e.g., Glu51, Glu108, Glu112) or that strongly deviate from this shape in at least one of the BLG forms (e.g., Glu44, Asp137, His146, His161, Cter), which can be observed by comparing Figure 5 with the individual protonation curves presented in the SI (Figure S9). Furthermore, the spline-based profiles are less sensitive to the uncertainty of the midpoint titration: e.g., although the p*K*_a_ estimated for Asp137 in the dimer is essentially qualitative, the errors of its average protonations are not high (Figure S9), which is reflected in the thin error envelope that it displays in Figure 5. As observed here, the spline-based integration method is a robust procedure that does not require the data to obey any particular shape and can be applied to atypical sites, whereas the Hill-based integration method may lead to significant cumulative errors even for sites that just slightly deviate from the typical Hill curve. Therefore, we focus our analysis on the dimerization free energy curve obtained with the spline-based integration method.

According to our results, the dimeric form of BLG is most favorable around the isoionic point (∼pH 5), as could be expected, and least favorable at pH 3. At higher pH values, a plateau can be observed. A free energy minimum around the same pH value is found in the experimental data, and despite the considerable dispersion of points obtained with different biophysical techniques, there is a general good agreement with our simulations at high pH values. However, at low pH, our simulations seem to overestimate the dimerization free energy by 2–3 kcal/mol.

In Figure 5, we can identify the sites that most contribute to the change in the dimerization free energy, which are also the ones whose titration curve most changes upon dimerization. Most of them favor association at increasing pH (e.g., Glu108, Asp129, Asp130, Asp137, His146), though Asp33, Glu89, and Glu158 contribute significantly for dissociation at those pH values. With a different profile, the C-terminal region groups His161 and the C-terminus itself display a peak around pH 5. Despite most of these residues being located at or near the interface, there are exceptions such as Glu108, Glu158, and Glu89.

### 3.3 Protonation Correlations

**Figure 6:**
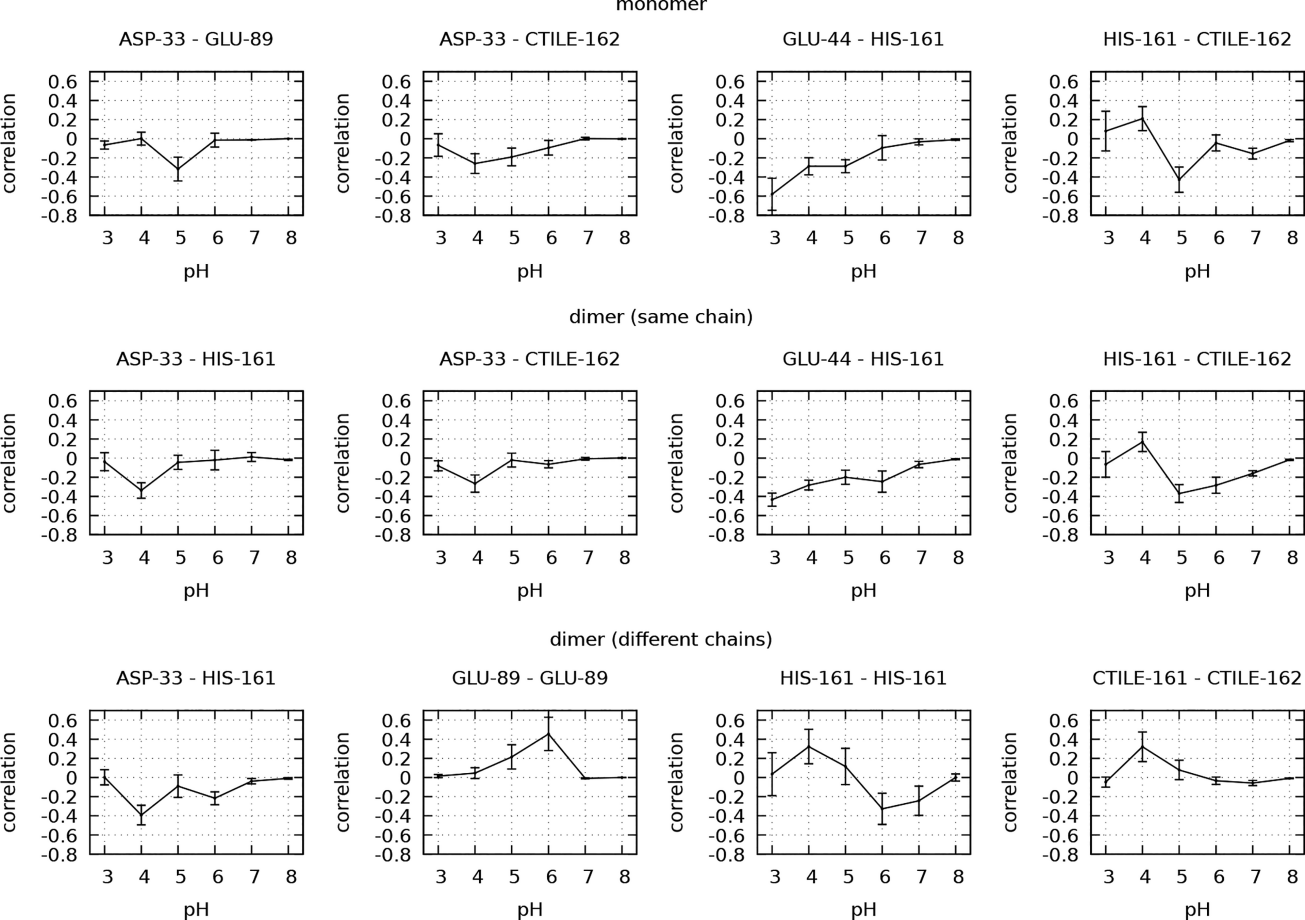
Correlation coefficient of the protonation of selected pairs of sites as a function of pH, for the monomer and the dimer (intrachain and interchain).

The protonation correlations among all sites were calculated using the Pearson’s correlation coefficient.^71,88^ This type of analysis may help identify key functional residues involved in electrostatics-dependent mechanisms.^71,94−98^ If a pair of titratable sites tends to exhibit opposed proton occupancies (empty/occupied) during the simulation, it will have a negative correlation, which can reach the limit value of −1 if they never display identical occupancies. Conversely, if a pair of titratable sites tends to exhibit identical proton occupancies (empty/empty or occupied/occupied), it will have a positive correlation, which can reach the limit value of +1 if they never display opposed occupancies. If the occupancies of two sites are completely unrelated, their correlation is zero. Negative correlations are the most widespread and easy to understand, typically resulting from the direct electrostatic interaction between pairs of sites, as when two nearby Asp residues tend to avoid the less favorable configuration where both are negatively charged (i.e., where both are empty). Positive correlations are not as common or intuitive, typically resulting from indirect interactions that arise from a chain of direct interactions involving three or more sites; for example, if three Asp residues display a roughly linear spatial arrangement, the central one may have negative correlations with the others (due to the avoidance of two negative charges in close proximity), while the two outermost residues may end up being positively correlated due to the “mediation” of the central one (when the central one is occupied they tend to be simultaneously empty and vice versa). When multiple titratable sites are interacting, the interplay of direct and indirect interactions may result in a complex network of negative and positive correlations. A set of maps depicting the correlations among sites is shown in Figures S10–S12, and the change in the correlation coefficient with pH is shown for a set of selected pairs in Figure 6.

In general, dimerization leads to a higher number of correlated sites, most correlations are lost at pH 7–8, and they are most abundant at pH 4. Strong correlations may involve residues located far apart in the protein structure (e.g., Glu89 of both chains in the dimer at pH 6 and Glu44 and Asp137 of the same chain in the dimer at pH 3), and in both the monomer and dimer simulations, strong positive correlations were observed besides the more usual negative ones. Some of the positive correlations can be understood through the mediation of a third site: e.g., in the dimer, at pH 5, Glu89 is positively correlated with the C-terminus of the other chain, and both directly interact and are negatively correlated with Asp33 of the Glu89 chain (see Figure S11 in the Supporting Information). In other cases, it is not easy to identify a chain of mediators responsible for the long-range positive correlations: e.g., in the dimer, at pH 5, no mediators were found that could explain the positive correlation between Glu89 of the two chains, even looking at correlations as small as ±0.15 (see Figure S11 in the Supporting Information). Overall, the observed networks of correlations are complex and subtle, making it difficult to disentangle all the underlying cumulative effects established among sites.

**Figure 7:**
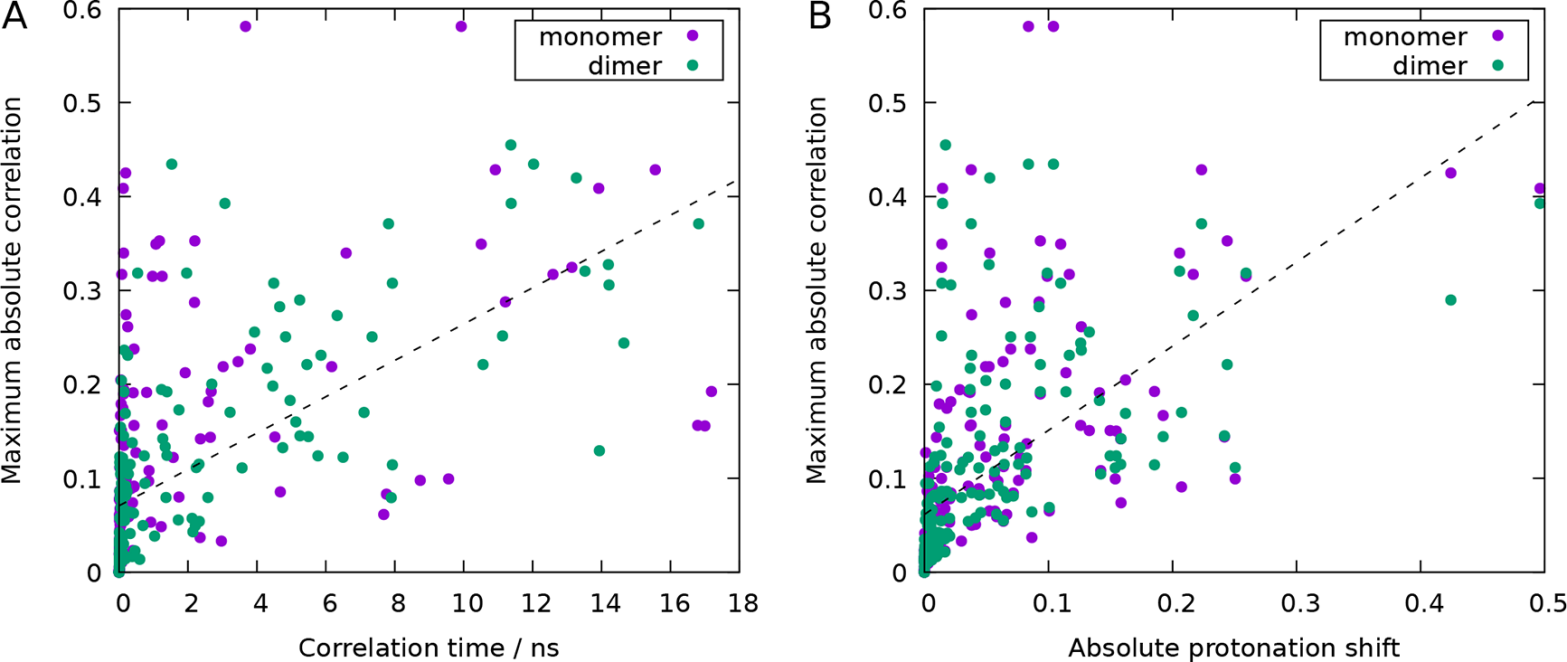
Scatter plots of the protonation correlations of each site versus its occupancy correlation time (A) and the absolute value of its monomer-to-dimer protonation shift (B). Each point corresponds to a site at a particular pH value, and the value plotted in the *y* axis is the maximum absolute value over all protonation correlations involving that site. The correlation time was estimated as the time at which the autocorrelation function^99^ of the proton occupancies (0 or 1) becomes lower than 0.1. The protonation shift is the difference between the protonation of the monomer and the dimer, in absolute value, which determines ΔΔ*G*_*i*_ (eq 5). Scatter plots very similar to A and B are obtained if, instead of the maximum absolute correlation, we use the standard deviation of the (signed) correlations; using the average correlation is not helpful, since it is always close to zero due to cancellation of positive and negative values.

Interestingly, we observe that the sites most frequently involved in stronger correlations tend to exchange protons at an unusually low rate, as seen from the correlation times computed from the autocorrelation function of the proton occupancies of each site (Figure 7A; see also Figure S13 for the correlation time of each site versus pH). Correlation times higher than 10 ns are observed, showing that a substantial kinetic trapping can occur. This suggests that the correlation networks, which become even more extensive upon association, tend to keep the system restricted to specific configurations of protonation states, which collectively preclude some protonation fluctuations and largely determine the protein charge distribution.

Remarkably, the sites that are more frequently or strongly correlated also tend to be the ones that contribute significantly to the dimerization free energy (e.g., Asp33, Glu89, Asp137, His161, C-terminus). The sites with higher ΔΔ*G*_*i*_ contributions are those whose protonation most changes upon dimerization (eq 5), which, as seen in Figure 7B, are indeed the ones with stronger correlations. As observed before, most of these residues are located at the interface region but not all. The complex network of correlations that is observed in BLG (see Figures S10–S12 in the Supporting Information) may explain the observed long-range effects, such as the correlation of distant sites or the large contribution to the dimerization free energy of noninterfacial sites. This makes it difficult to isolate the role of a single site or to predict the effect of point mutations.

### 3.4 Electrostatic Complementarity

As correctly predicted by our simulations, the optimal dimerization pH for BLG is around its isoionic point. This might simply reflect the fact that the overall repulsion between the like-charged partners of the homodimer will be minimal at this pH, when their net charge is around zero, which can be interpreted as an indication that the dimerization could be hydrophobic. On the other hand, experimental studies have observed that the monomer–dimer equilibrium is affected by the presence of ions^4,43,44^ and that the dimer interface contains charged groups and salt bridges,^56^ suggesting that electrostatics is also important, perhaps involving some degree of charge complementarity of the two interface surfaces. Indeed, some works propose that both hydrophobicity and electrostatics play an important role in BLG dimerization.^37,38,100^

The analysis of protein electrostatics often consists of performing a PB calculation and mapping the resulting electrostatic potential either on the protein surface or on isosurface countours that extend into the solution.^101,102^ This is typically done by using a single conformation and a single set of protonation states of the system, but that would be a step back with respect to our current approach, since it would ignore the explicit sampling of conformations and protonation states done by using the CpHMD simulations. Therefore, we adopted instead an approach that, in a sense, turns around the rationale of the PB model: since the charge distribution of the protein determines the distribution of the nearby solution ions, this ionic distribution can be used as a direct fingerprint of the protein electrostatics. Thus, the analysis of the spatial densities of both anions and cations will directly reveal the regions near the protein surface that tend to be positive (accumulating anions) or negative (accumulating cations). To check its performance, we also computed ionic densities with a PB model using a central structure and average charges (see section 2.5).

**Figure 8:**
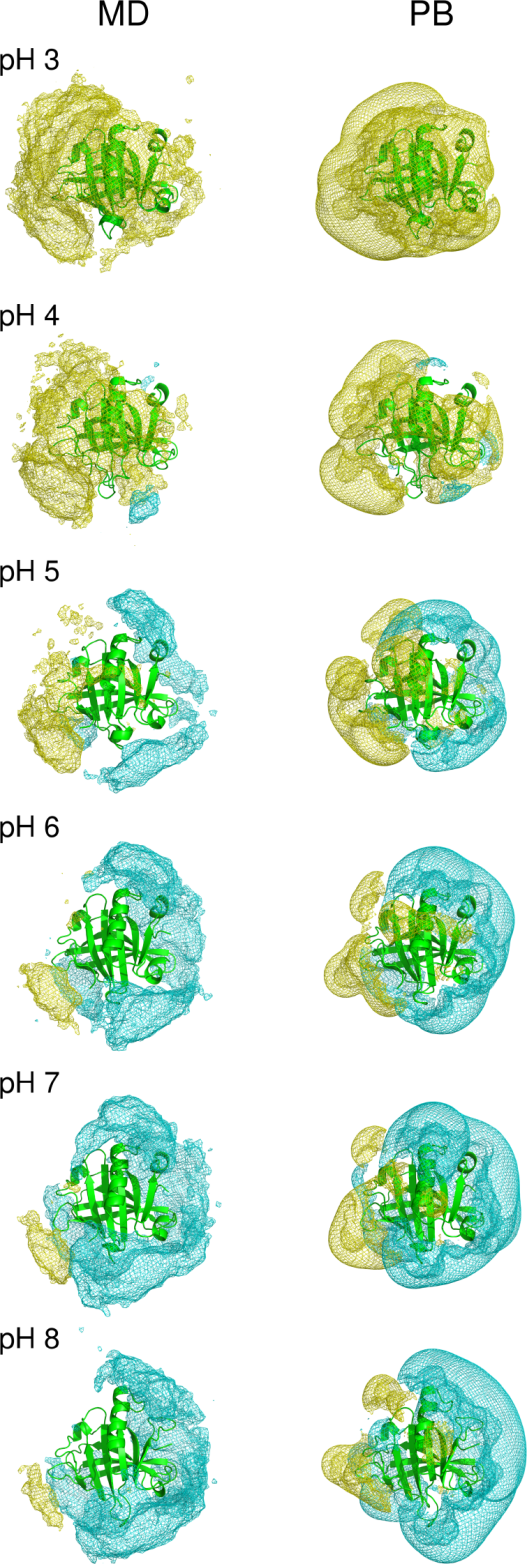
Density contours of 200 mM for the Na^+^ (cyan) and Cl^–^ (yellow) ions in the monomer, at different pH values, computed from either the MD simulations (left) or the PB model (right).

The ionic densities obtained from the monomer simulations are presented in the left panel of Figure 8, which shows the iso-density contours encompassing regions where the ion concentrations are at least twice their bulk value. The protein is essentially enveloped by Cl^–^ ions at low pH, reflecting its strongly positive net charge (see Figure 2), and as pH is increased, it gradually acquires patches of Na^+^ ions that eventually dominate the distribution at high pH, when the protein exhibits a negative net charge. The mixed population of Na^+^ and Cl^–^ patches is more marked at pH 5, near the isoionic point. The right panel presents the results obtained with the PB model, showing a reasonable overall agreement with the left panel, even though the PB density contours tend to be more extensive and smoother. This indicates that, although a judicious choice of structure and charges can produce PB-derived ionic distributions reasonably similar to those actually observed in the CpHMD simulations, the bias introduced by using a single structure cannot be avoided. Furthermore, ion–ion correlations are lost in the PB approximation,^103^ which might explain some of the observed differences between the MD and PB contours.

Similarly to the monomer case, the ionic densities obtained from the dimer simulations exhibit a higher mix of Na^+^ and Cl^–^ density patches near the isoionic point (Figure S14) . Again, the PB densities show a reasonable overall agreement with the MD ones.

**Figure 9:**
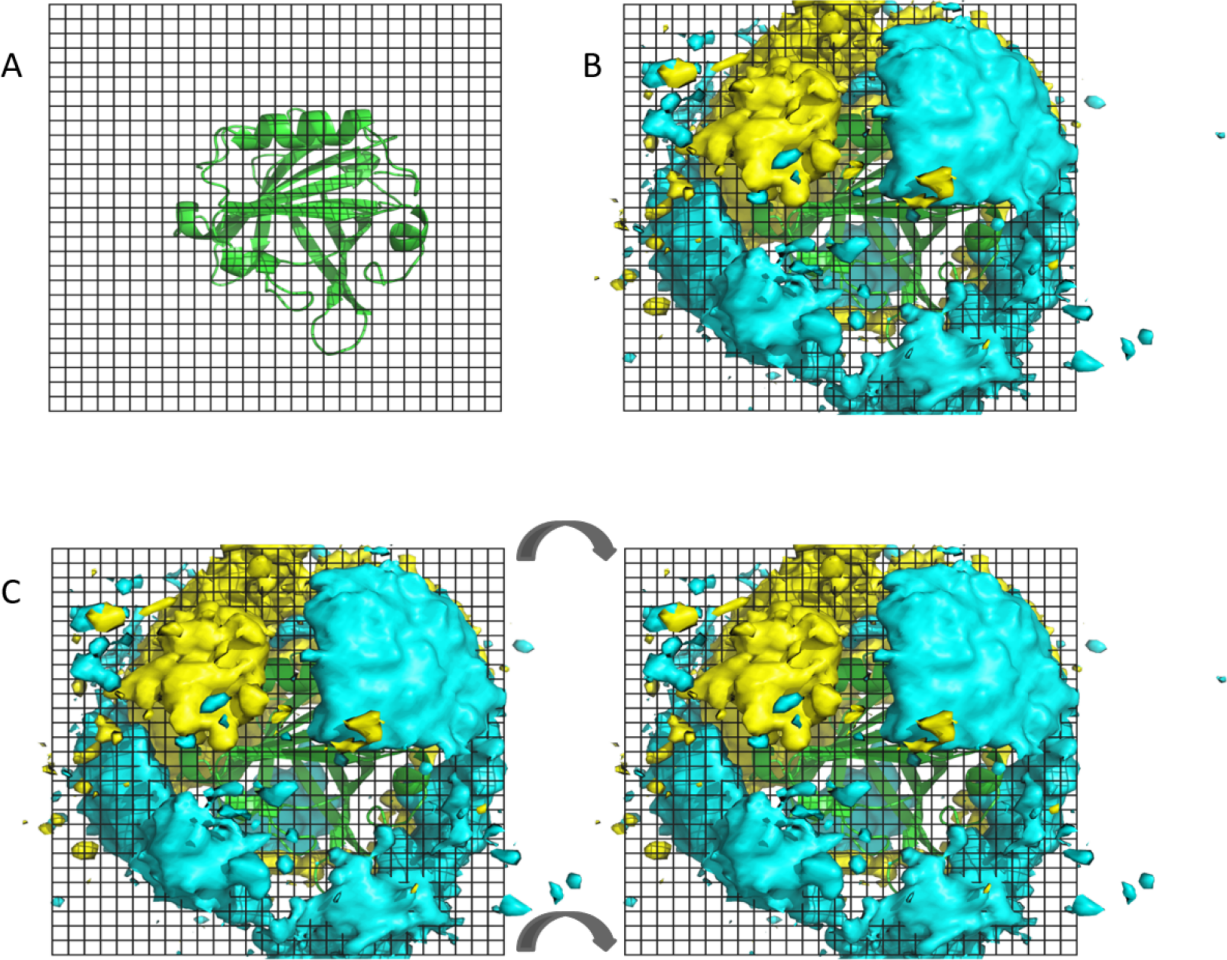
Electrostatic complementarity at the dimer interface. (A) Monomer face that would be found at the interface of the dimer, with the plane between both partners shown as a grid. (B) Density contours of 150 mM for Na^+^ (cyan) and Cl^–^ (yellow) ions computed from the monomer MD simulations at pH 5. (C) Electrostatic complementarity of two potentially dimerizing partners. The two images are arranged in analogy to the facing pages of an open book; when the book closes, the two faces meet in the correct antiparallel conformation.

If we inspect the region of the monomer that becomes its contact surface upon dimerization, we find that the ionic density contours indicate some degree of electrostatic complementarity between the two potentially associating partners, especially at pH 5, as shown in more detail in Figure 9. The ionic density in the lower half of the image protrudes away from the interface plane (grid) mostly at the bottom, not “in front” of the protein, in a region that would remain occupied by solvent even after dimerization. This indicates that the monomer has no significant net charge in the lower half of the protein interface plane. In contrast, the ionic density in the upper half of the image is located right “in front” of the protein, with a high concentration of Cl^–^ on the left and Na^+^ on the right. This asymmetry, which is largely aligned along the interface helix, indicates that the upper left of the protein is positive, while the upper right is negative, thereby forming a strong interfacial dipole (in the opposite direction of the main-chain α-helix dipole, whose effect is already included). In order to get dimerization, two partners would have to eventually come into contact in the antiparallel arrangement observed in the dimer structure, which would put their interfacial dipoles in an electrostatically favorable head-to-tail orientation. Therefore, these interfacial dipoles create an electrostatic complementarity that may help the dimerization process. Of course, a full characterization of the role of electrostatics on the dimerization of BLG would require studying the intermediate stages of the association process because, as seen in section 3.1 for the final dimer configuration, each of them would affect protonation equilibrium, thereby modulating electrostatics. Nonetheless, Figure 9 indicates that the monomers already exhibit some degree of electrostatic complementarity that may mediate some intermediate stages of the dimerization process. Furthermore, as pH moves away from the isoionic point, this complementarity decreases and eventually vanishes (Figure S15). This trend is consistent with the experimental and computed free energies of dimerization (Figure 4), reinforcing that, indeed, electrostatics plays an important role in the dimerization process.

### 3.5 Dimer Configurations

**Figure 10:**
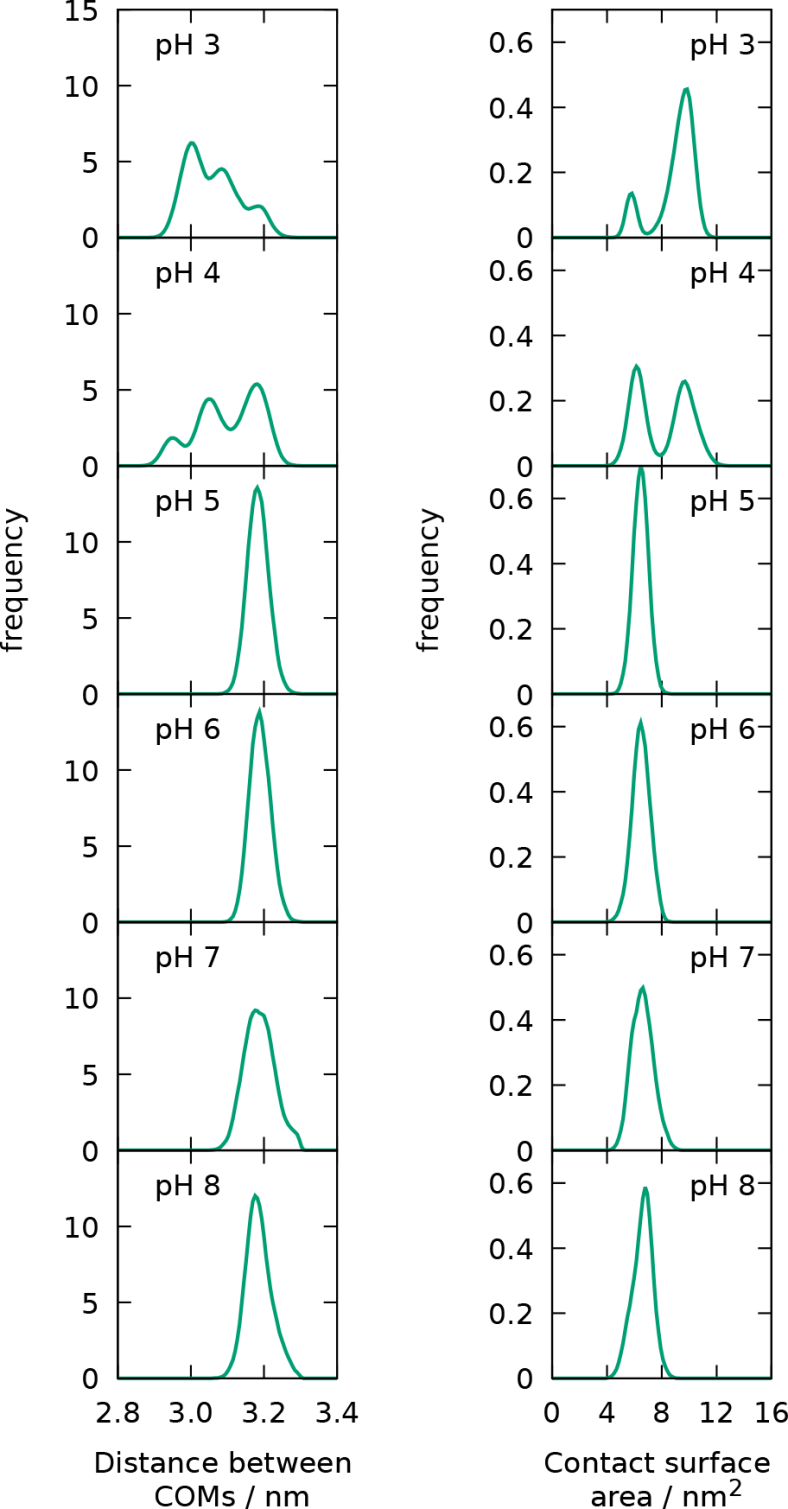
Histograms of the distance between the centers of mass of the two dimer partners (left panel) and of the contact surface area between the two (right panel).

Although the dimeric form of BLG is more predominant at pH values near its isoionic point, this does not imply that the two dimer partners are necessarily closer to each other at that pH. Indeed, analysis of the dimer simulations shows that the histogram of the distance between the centers of mass (COM) of the two dimer partners (Figure 10) has peaks around 3.2 nm for the pH range 5–8, while peaks at smaller distances become gradually populated as the pH is lowered, with a predominant peak at around 3.0 nm at pH 3. A corresponding trend is observed in the histogram of the contact surface area between both partners (Figure 10), where a single peak is observed around 6–8 nm^2^ for pH 5–8, while higher areas are populated as the pH is lowered, reaching a peak around 10 nm^2^ in the distribution at pH 3. Thus, the dimer appears to exhibit a more tightly arranged *compact state* predominant at pH 3 and a *relaxed state* predominant at pH 5–8. At pH 4, both types of configuration (and intermediate ones) are observed.

**Figure 11:**
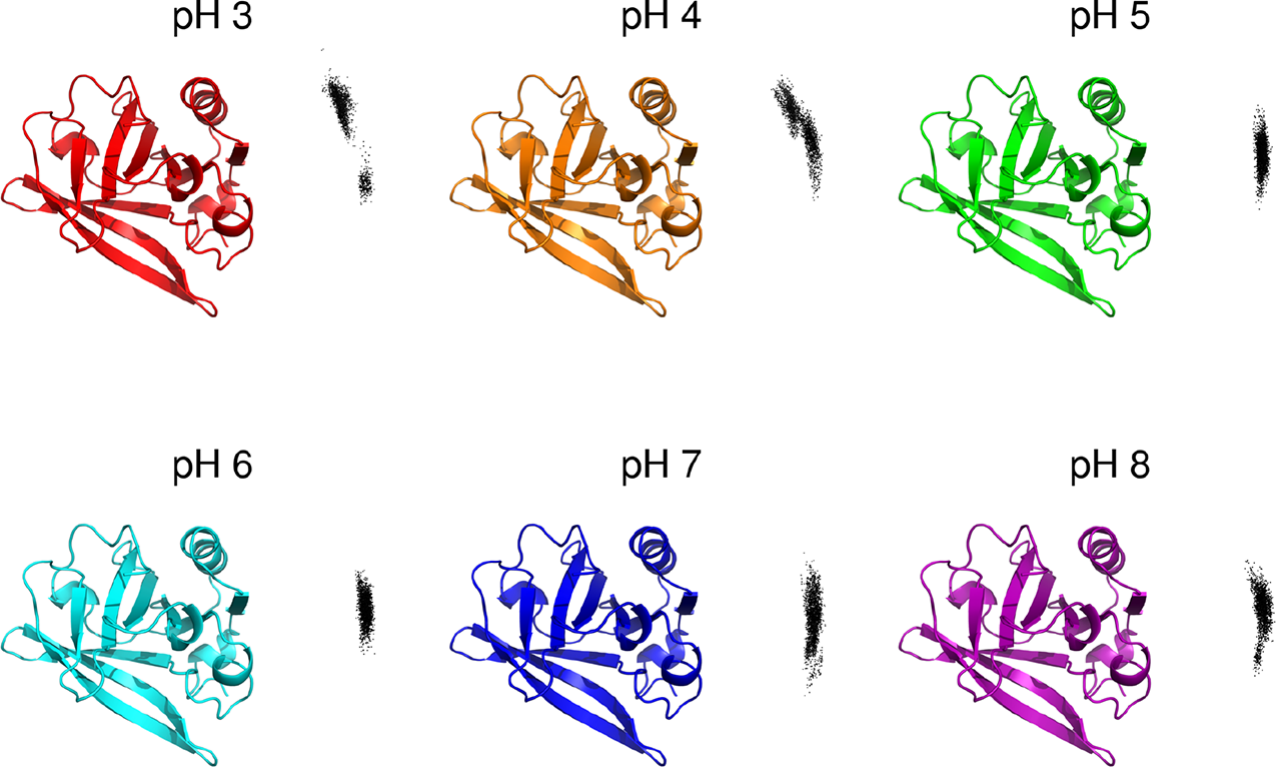
Relative positions of the COM of one dimer partner (dots) against a reference structure of the other partner (ribbon cartoon). The reference structure (crystallographic structure) was used to fit each of the partners in turn, while the COM of the other partner was represented as a dot. Snapshots at regular intervals of 50 frames were used.

In order to characterize the dimer configuration in more detail, we analyzed the relative position of the dimer partners, displaying the center of mass of one partner and a reference structure to which the other partner was fitted (Figure 11). The observed distributions of points are in line with the previous results, roughly clustering into two major regions: a “bottom” region that is the one populated at pH 5–8, and a “top” region that is the one overwhelmingly populated at pH 3. At pH 4, both regions are significantly populated, analogous to what was observed in the COM distance and contact surface area histograms.

**Figure 12:**
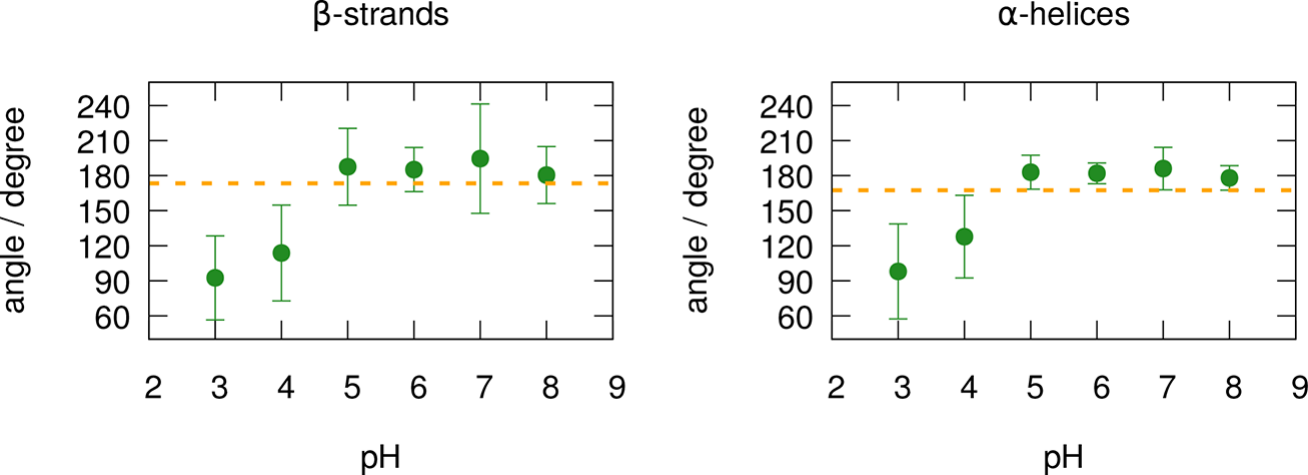
Dihedral angles between the two nonbarrel β-strands (left) and the two α-helices (right) in the dimer interface. The points and error bars were calculated as, respectively, the means and standard deviations of the angle average of each of the 8 replicates. The dihedral angles were defined using main chain atoms from the two opposite ends of each secondary structure motif (see Figure S16 in the Supporting Information). The dashed lines represent the average dihedral angles of the open and closed crystallographic structures used in this work (1BSY and 3BLG were obtained, respectively, at pH values 7.1 and 6.2;^36^ see section 2.1).

**Figure 13:**
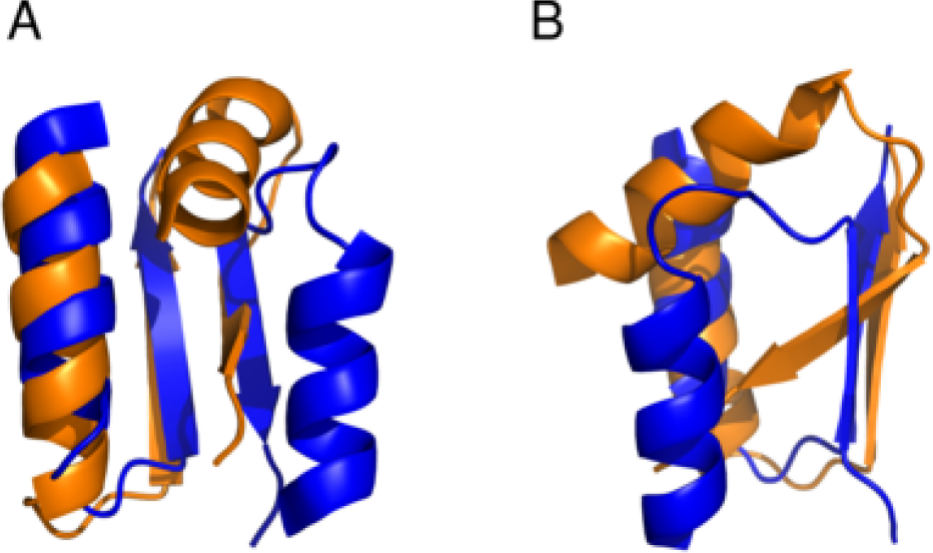
Superimposed dimer structures at pH 3 (orange) and pH 5 (blue), obtained by fitting one of the partners. For clarity, only the interface main structural elements are represented, focusing on the alignment alterations of the α-helices (view A) and of the nonbarrel β-strands (view B). The structures shown are representative of the most populated orientations at, respectively, pH 3 and 5, the latter being also representative of the pH range 5–8.

In the crystallographic structures of the BLG dimer, the three-turn α-helix of each chain is part of the interface region, where it is paired with the helix of the other partner in an antiparallel arrangement (shown in yellow in Figure 1). A similar situation is observed for the nonbarrel β-strand, which is also paired with the corresponding strand of the other partner in an antiparallel arrangement (shown in orange in Figure 1). Therefore, we can use the dihedral angles formed by the helices and by the nonbarrel strands to measure the relative orientation of the two partners. As seen in Figure 12, the average dihedral angles between the α-helices and between the β-strands show very similar profiles. At pH 5–8, both dihedrals remain around 180°, corresponding to the antiparallel orientation observed in the crystallographic structures (obtained in that pH range), shown by the dashed lines in the figure. However, the average orientation changes to almost perpendicular (around 100°) at pH 3, with intermediate orientations at pH 4. This relative rotation of the partners can be illustrated with snapshots typical of each pH, as shown in Figure 13: at pH 5 (blue), an antiparallel alignment is observed for both the α-helices and the β-strands, but that alignment has been lost at pH 3 (orange), as seen in the dihedral analysis. This relative rotation of the dimer partners is associated with the small displacement seen in Figure 11, which together seem to make possible the approximation and tighter association observed at low pH in Figure 10. Thus, as one goes below pH 5, these concerted configurational changes make the dimer partners rotate, slide, and approach relative to each other. Several carboxyl and amino groups exist in this interface region, some of which are titrating and/or involved in strong protonation correlations between pH 3 and 5 (e.g., Asp33, Asp130, Asp137, His161, and the C-terminus, mentioned in previous sections), in interplay with these pH-induced concerted changes.

**Figure 14:**
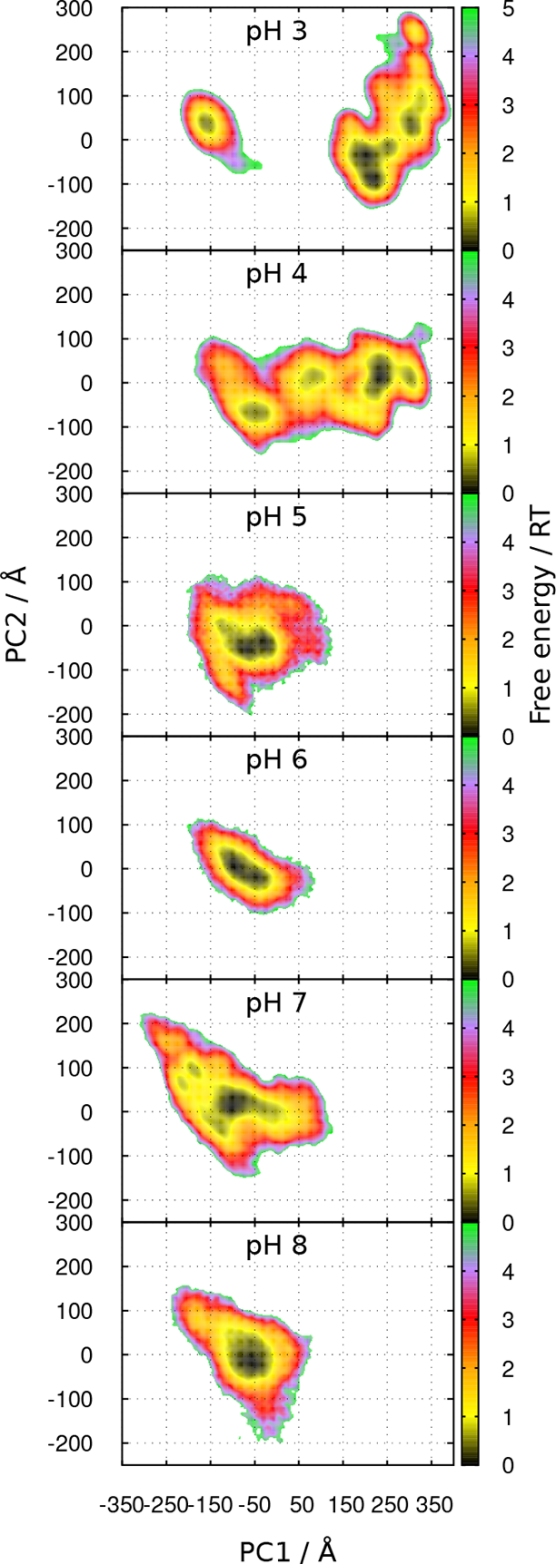
Free energy landscape over the first two principal components (PC1 and PC2) obtained from the PCA of the dimer configurations, at different pH values. See section 2.6 for methodological details.

**Figure 15:**
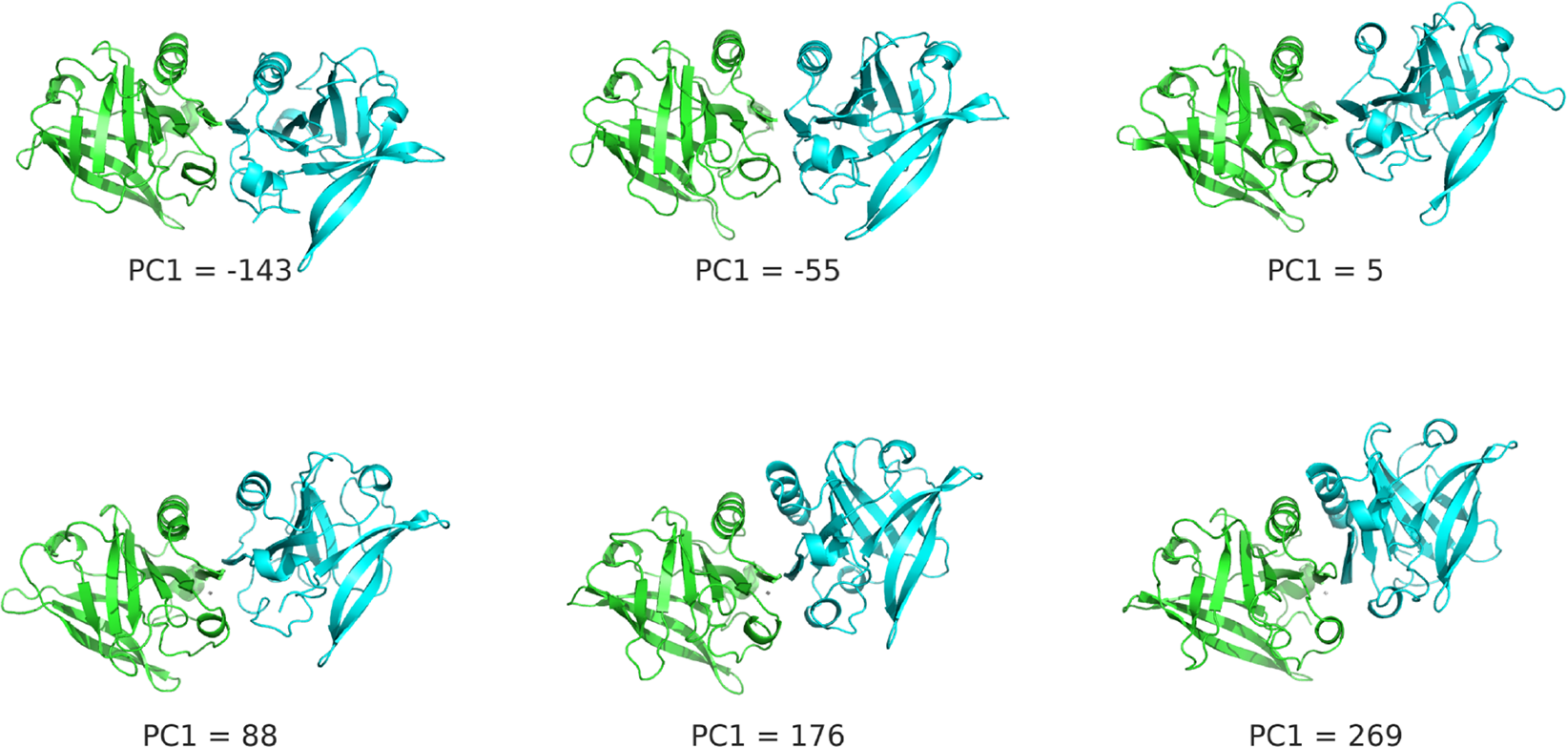
Selection of some representative frames of BLG taken along the PC1 coordinate, illustrating the relative rotation of the two chains that was captured along this PC. The chain in green was fitted to a reference structure, whereas the resulting coordinates for the other chain (blue) were used in the PCA. See section 2.6 for methodological details.

This dependency of the dimer configuration on pH can be further examined using a principal component analysis (PCA) of the arrangement of one partner relative to the other (see methodological details in section 2.6). Figure 14 shows the free energy landscapes in the space of the first and second principal components (PC1 and PC2) obtained from this PCA. For the pH range 5–8, the configurations cluster roughly in the same region on the left of the plots (−300 ≲ PC1 ≲ 100), whereas those from pH 3 are mostly found in the right region (100 ≲ PC1 ≲ 350), with a small population on the left region. Analogous to the previous analyses, the configurations at pH 4 appear to populate the regions typical of either pH 3 or pH 5–8 and also some intermediate states. Thus, we find again a split into two major sets of dimer configurations: a *compact state* typical of pH 3 and a *relaxed state* typical of the pH range 5–8, both of which are observed at pH 4. Interestingly, the transition between these two states seems to be well captured by the PC1 coordinate, with the transition taking place around PC1 ≈ 100. Figure 15 shows a set of dimer configurations taken along the PC1 coordinate, in which the rotation of the two chains relative to each other is clearly observed (also in Film1.avi given as SI). Indeed, as shown in the SI, the PC1 averages at different pH values are nicely correlated with the averages of the COM distances (Figure S17) and of the rotation dihedrals (Figure S18), despite some dispersion of the distributions at pH 3 and 4.

Overall, the results in this subsection indicate that the configuration of the BLG dimer seems to experience a pH-induced transition between two major states: a *relaxed state* observed in the pH range 5–8 and a *compact state* observed at pH 3, with both states being present at pH 4. In addition, the results show that, although BLG dimerization is known to be higher at pH 5, this is not related to how closely or tightly associated the two partners are, since a closer/tighter association is actually observed for the *compact state* typical of pH 3.

### 3.6 EF Loop

A reversible conformational change near pH 7.5 was detected via optical rotation by Tanford and co-workers, who also suggested a concomitant release of a buried carboxyl group, whose protonation would explain a steepening of the global titration curve observed near that pH.^34,47^ Forty years later, Qin et al.^36^ proposed that the Tanford transition corresponds to the movement of the EF loop, which forms a lid at the entrance of the calyx that was observed closed in crystallographic structures obtained at pH 6.2 and open at pH 7.1 and 8.2. This loop contains a carboxyl group, Glu89, which presumably corresponds to the one proposed by Tanford et al., since it is buried (and presumably neutral) in the closed conformation^36,56^ and exposed to the solvent (and presumably charged) in the open one.^36^

**Figure 16:**
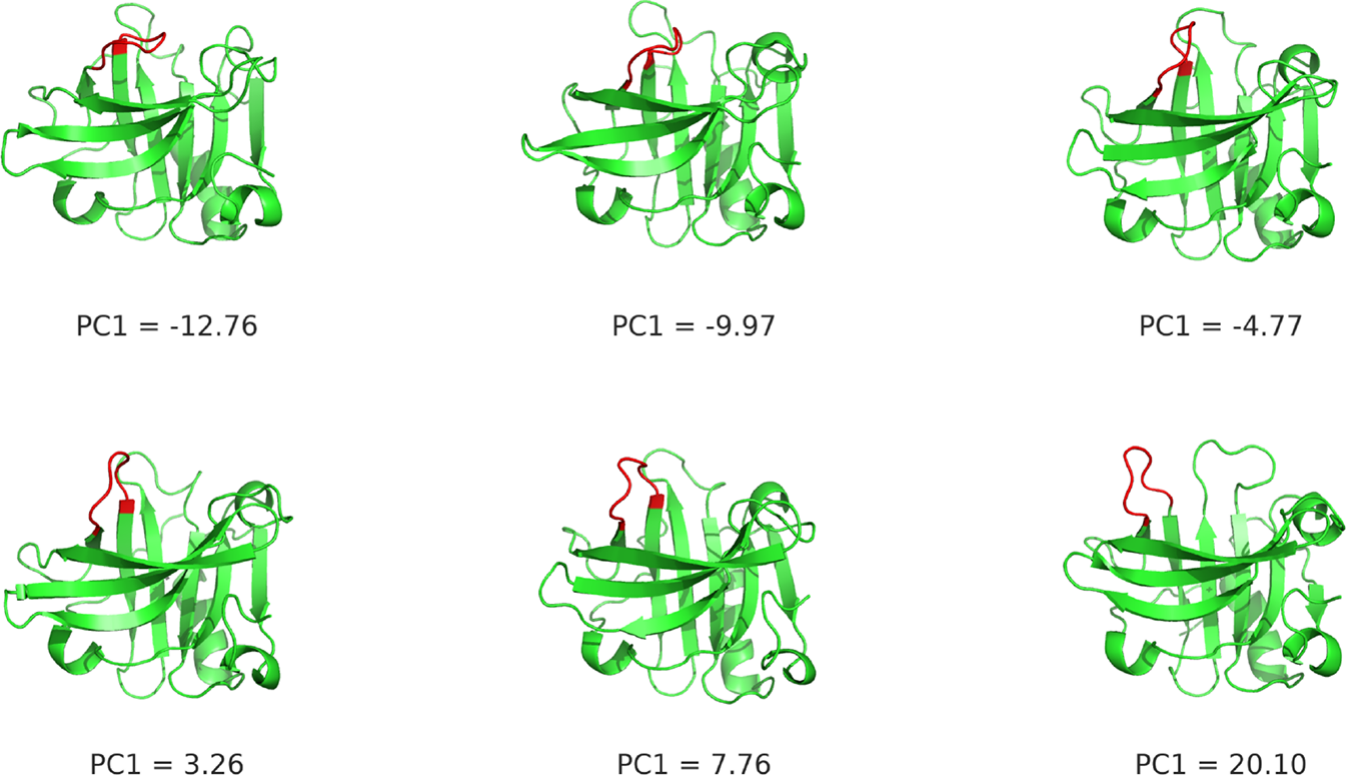
Different frames of the EF loop, along the PC1 coordinate. This illustrates the EF loop transition observed in PC1, from closed to open. The EF loop is represented in red.

**Figure 17:**
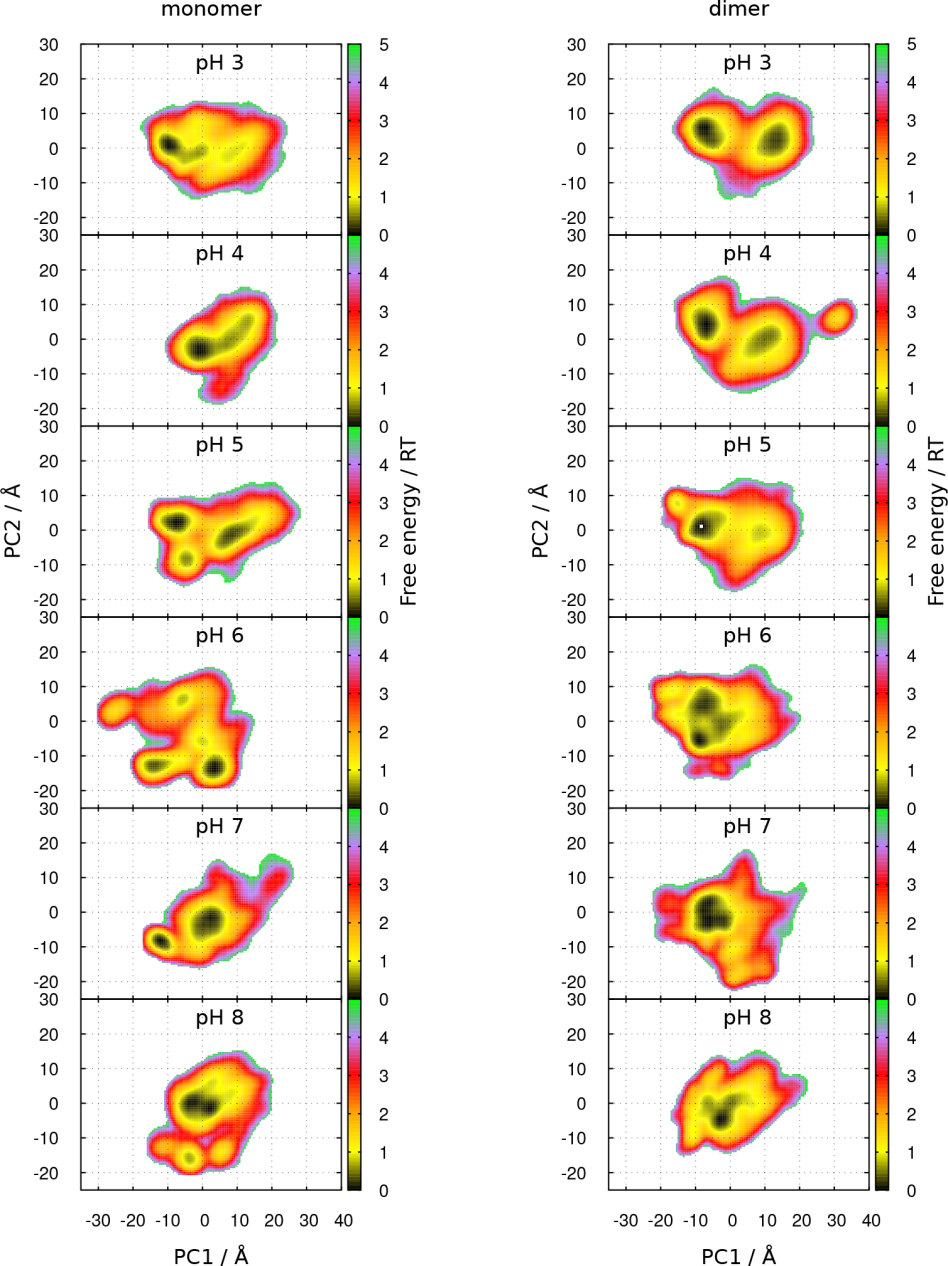
Free energy landscapes in the 2D space obtained from the PCA of the EF loop (residues 84–90), for the monomer and dimer at different pH values. See section 2.6 for methodological details.

**Figure 18:**
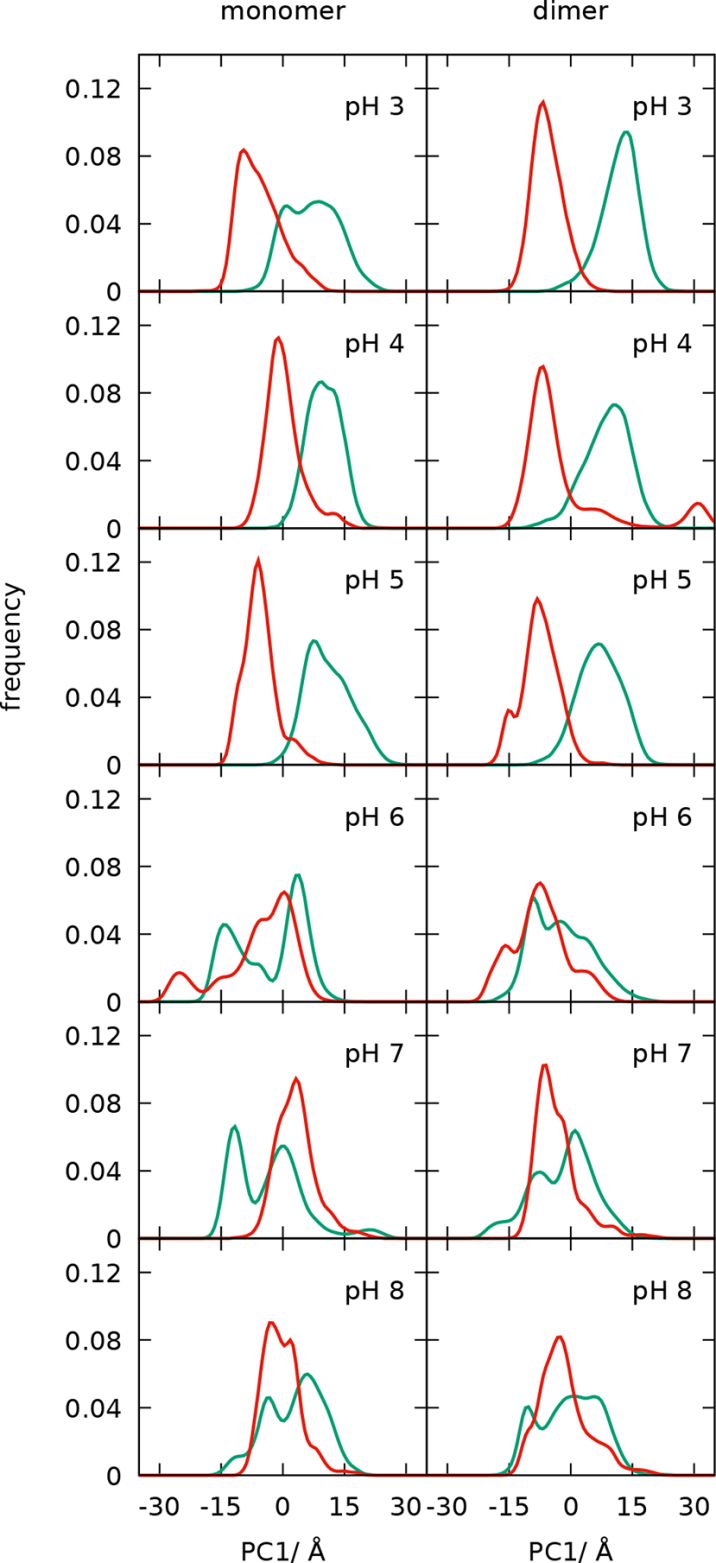
Histograms of the PC1 values from the EF loop analysis shown in Figure 17, separating the open (green) and closed (red) initiated set of conformations, for the monomer and dimer at different pH values.

A PCA was performed using the coordinates of the EF loop after fitting to a crystallographic structure the backbone of the whole protein with the exception of the EF loop itself (for details see section 2.6). This analysis captured the opening/closing movement of the loop, as verified by inspection of the frames along the first PC in Figure 16 (also shown in the SI, Film2.avi). The free energy landscapes in the space of the first two PCs at each pH value are shown in Figure 17. In addition, the two sets of configurations sampled from simulations started with either a closed structure or an open one can be compared in Figure 18, where the respective histograms of the first PC are represented. The histograms show that at pH 3–5 the loop is trapped in the initial configuration, but at higher pH, the movement of the loop is already observed, which could be explained by a lowering of the energy barrier for this transition, in either direction. In fact, as observed in Figure 17, the topography of the energy landscapes changes with pH. In the case of the dimer, two main basins are observed at pH 3–5, and just a main one is observed at pH 6–8. At the low pH range, the energy barrier between these two basins seems to decrease with pH, but their true relative depths are unknown due to the observed conformational trapping. In the case of the monomer, the energy surfaces are more rugged, perhaps due to the smaller sampling (half the number of dimer replicates), even though some of the basins have similar positions in the monomer and dimer at the same pH. Additionally, in both the monomer and dimer, the basins at pH 6–8 seem to be moving in the closed-to-open direction (see Figure S19 in the Supporting Information) in agreement with the conformational transition observed at this pH range in crystallographic structures.^36^

**Figure 19:**
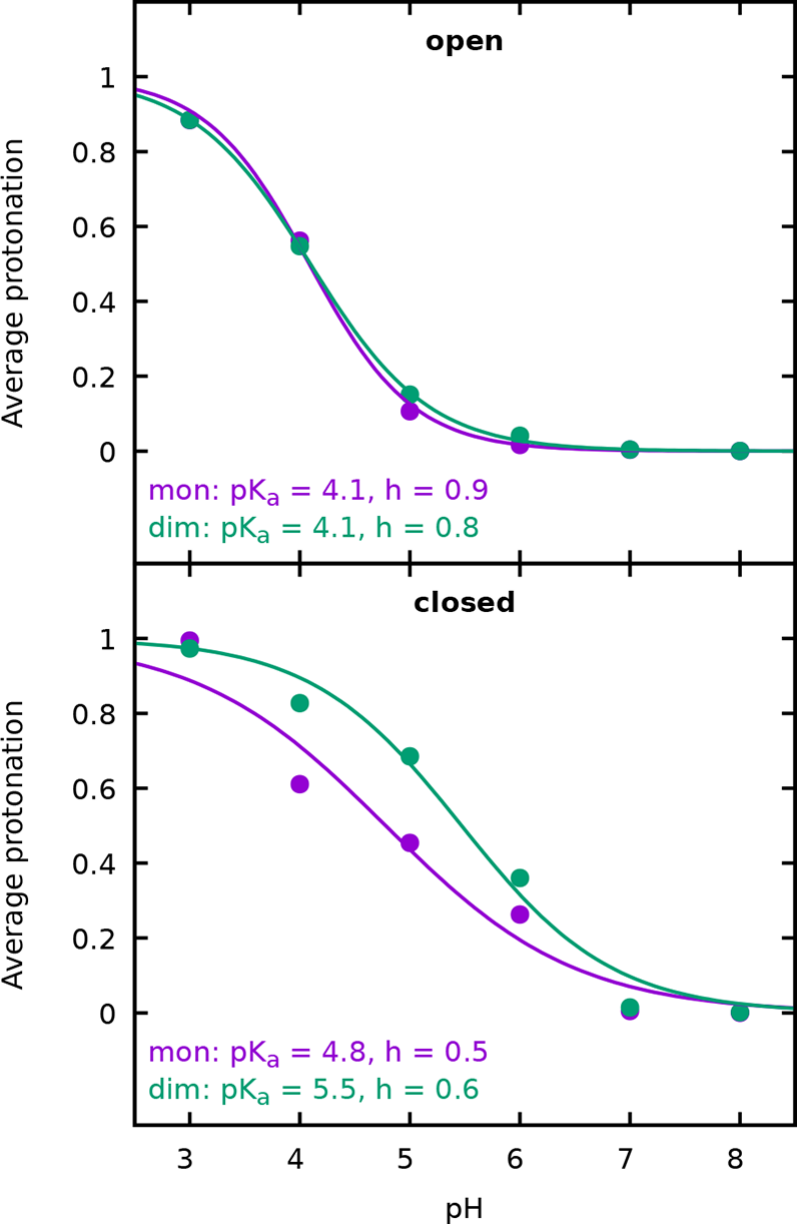
Average protonation of Glu89, in the monomer (purple) and dimer (green), considering the simulations started either with an open or closed loop. The p*K*_a_ values and Hill coefficients *h* obtained from the fit of a Hill curve are shown.

Figure 19 shows the protonation curves of Glu89 (presumably associated with the loop conformational transition), as obtained from the set of simulations started with either the closed or open loop. Consistently with the conformational trapping observed at low pH, the simulations started with an open loop give a p*K*_a_ typical of a solvent-exposed carboxyl group (around 4), while those started with a closed loop give higher p*K*_a_ values (4.8 for the monomer and 5.5 for the dimer), as expected for a less solvent-exposed group, but still substantially lower than the pH of the Tanford transition, around 7.5. Also, a gentler slope is observed for the set of simulations started with the closed loop, which usually indicates a stronger dependence on the protonation of other sites (as expressed by eq 7). The observed conformational trapping may somewhat affect the protonation sampling of Glu89 at low pH, but this should not markedly affect the overall analyses of the dimerization, especially considering its distance from the interface.

In conclusion, although the simulations were successful in detecting the EF loop transition at higher pH values, observing a displacement of the open/closed equilibrium consistent with the proposed role of this loop in the Tanford transition, they seemed too short to provide an adequate sampling of this movement at low pH. In fact, NMR spectroscopy observations suggest that motions in the top part of the loop only occur in the millisecond time scale or even slower.^104,105^ However, though a detailed study of this transition may be the object of future research, it falls out of the scope of the present work.

## 4 Conclusions

The present study proposes a general approach to study the pH dependency of protein–protein association using CpHMD simulations, applying it to investigate the dimerization of BLG. The results are in very good agreement with the available experimental data and, in addition, reveal some features that would remain unnoticed by usual methodologies.

The computed isoionic point of the BLG dimer is in excellent agreement with the experimental ones,^47,48^ and aside from a small deviation around pH 7, its global titration curve between pH 3 and 8 nicely matches the potentiometric data.^48^ Although no comparison with experimental data is possible for the individual titratable sites of BLG, they display a varied and rich behavior, with several of them exhibiting unusual p*K*_a_ values and/or titration curves (e.g., His161, Asp98), which in some cases are affected by dimerization.

The pH dependence of the dimerization free energy of BLG, computed using a Wyman–Tanford linkage relation, is also well reproduced, particularly the maximum dimerization near the isoionic point (Figure 4). We also identified the titratable sites with higher contributions to this free energy profile, which are the ones whose protonation curves are most affected by dimerization (e.g., Asp33, Asp137). The total and individual free energy profiles, computed with a new method using thermodynamically based splines, show some differences with the more approximate profiles computed using fitted Hill curves, indicating that the latter approach can introduce non-negligible errors.

The analysis of correlations between the proton occupancies of pairs of titratable sites reveals many correlated pairs that, at some pH values, form large correlation networks that extend across BLG and whose concerted effect gives rise to unexpected strong correlations between distant pairs of sites (e.g., between Glu89 of both dimer partners). Remarkably, the sites most frequently involved in strong correlations turn out to be the ones with high contributions to the dimerization free energies (e.g., Asp33, Asp137), and they tend to be slow proton exchangers (i.e., their occupancies have higher correlation times).

Ionic densities are used as a fingerprint of the protein charge distribution, revealing that, near the isoionic point, the interface region of BLG exhibits electrostatic complementarity between the two dimer partners (most markedly than at other pH values). This complementarity may mediate the association of the partners, reinforcing the importance of electrostatic interactions in the dimerization process.

Two major configurational forms of the BLG dimer were observed: a *compact state* at pH 3–4 and a *relaxed state* at pH 4–8. The relaxed state is the one also observed in the crystallographic structures (at higher pH values), with the interfacial helices and β-strands in an antiparallel alignment, while the compact state results from the approximation and relative rotation of the two partners, which makes those interfacial elements roughly perpendicular. The transition takes place around pH 4 and is nicely captured by a PCA analysis using a judicious choice of coordinates.

The EF loop, whose motion is presumably associated with the Tanford transition of BLG,^34,36^ was also analyzed. Although some tendency for the opening of the loop can be observed in our simulations around the pH values of the Tanford transition, its motion seems very slow, as previously observed in experiments and simulations.^104,105^ Therefore, much longer simulation times or enhanced sampling methods would be needed to properly study this process.

Overall, the present results indicate that BLG has an extensive network of slow-exchanging correlated sites, which, by restricting the possible protonation arrangements, may determine its charge distribution and end up modulating the pH dependence of its dimerization. This observation challenges the traditional view of attributing functional relevance to only a few key residues when, in fact, a somewhat large network of correlated sites may work in a concerted and delocalized way. This hypothesis could not have been reached through more standard methodologies that ignore the protonation–conformation coupling.

The set of analyses proposed here includes approaches that are not widely used, particularly in the context of constant-pH MD simulations. This is the case for the calculation of association free energies using a Wyman–Tanford linkage relation (calculated here with a new spline method), the inspection of proton occupancy correlations, and the use of ionic densities as a fingerprint of protein charge distribution. Altogether, the selection of analyses proposed in this work offers a compelling route to study the main aspects involving the pH dependency of protein association, as nicely illustrated for BLG dimerization.

## Appendix

This Appendix follows the formalism of linkage function theory,^23^ from which eqs 2 and 6 are usually derived, but is extended to a “microscopic” description of the binding state. As usual, we assume low protein concentrations, such that ratios of activities of its forms can be replaced with the corresponding ratios of concentrations.

The binding state of a macromolecule A with *s* binding sites for a ligand X is here represented by the vector **n** = (*n*_1_,*n*_2_,···,*n*_*s*_), where *n*_*i*_ = 0 or 1 depending on whether site *i* is empty or occupied by ligand X, with **0** = (0,0,···,0) denoting the ligand-free state. The total number of ligand molecules bound to A is *n* = ∑_*i*_ *n*_*i*_. Each of the 2^*s*^ different species is denoted as AX(**n**), and its concentration can be written as [AX(**n**)] = [AX(**0**)]β(**n**)*a*^*n*^, where *a* is the activity of ligand X, and β(**n**) is the equilibrium constant of the reaction

13

The total concentration of A can then be written as

14

where the sum runs over all 2^*s*^ states **n**, and *Q* = ∑_**n**_ β(**n**)*a*^*n*^ is a partition function. The fraction of species AX(**n**) is then

15

and we can write the ensemble averages *n̅* = ∑_**n**_ *nf*(**n**) and *n*_*i*_ = ∑_**n**_ *n*_*i*_*f*(**n**). Differentiation with respect to the natural logarithm of *a* then gives

16

17

and

18

where cov(*n*_*i*_,*n*) is the covariance between *n*_*i*_ and *n*. When H^+^ is the ligand, ln *a* = −ln(10)pH, and we get eq 7; and since the covariance is additive and cov(*n*,*n*) = var(*n*), we also get eq 6.

## Supporting Information Available

Dependence of fraction of dimer as function of BLG concentration; number of added ions and average system net charge; description of spline interpolation; description of bootstrap errors calculation; equilibration analyses, including time series of several properties; protonation curves of each individual site; dimerization free energies in literature; networks of protonation correlations; correlation times of proton occupancy; ionic concentration contours for dimer; electrostatic complementarity at different pH values; additional material for dimer configuration analyses; and average values of first PC of EF loop PCA, at different pH values (PDF)
Film1.avi: film illustrating structural transition along first principal component of dimer configuration PCA (AVI)
Film2.avi: film illustrating structural transition along first principal component of EF loop PCA (AVI)

## Supplementary Material

ct1c01187_si_001.pdf

ct1c01187_si_002.avi

ct1c01187_si_003.avi
