# Correction to “Approach to Study pH-Dependent Protein Association Using Constant-pH Molecular Dynamics: Application to the Dimerization of β-Lactoglobulin”

**DOI:** 10.1021/acs.jctc.2c00605

**Published:** 2022-06-29

**Authors:** Lucie da Rocha, António M. Baptista, Sara R. R. Campos

We detected a few minor mistakes in our article that are here corrected. In subsection 2.5 of Theory and Methods, we noticed a few inaccuracies: we used a triangular kernel estimator to compute the probability density of ions around the protein, instead of a Gaussian kernel estimator as stated before; we used a one-step focusing approach with grid spacings of 1.0 and a dielectric constant of 4 to solve the PB equation, which we forgot to add to the text. In the beginning of the Results and Discussion (section 3), there is a reference to Table S2 whose values have been corrected and presented in the Supporting Information accompanying this correction; the differences are minor and do not require any alteration in the text. In subsection 3.3 of the Results and Discussion, there was a mistake in the calculations of the monomer’s correlations: Figures 6 and 7 in the manuscript are replaced with the ones presented here, and a new Figure S10 is now provided in the Supporting Information accompanying this correction; these corrections do not affect the observed trends and, thus, the discussion and conclusions in the text do not require any changes.

**Figure 6:**
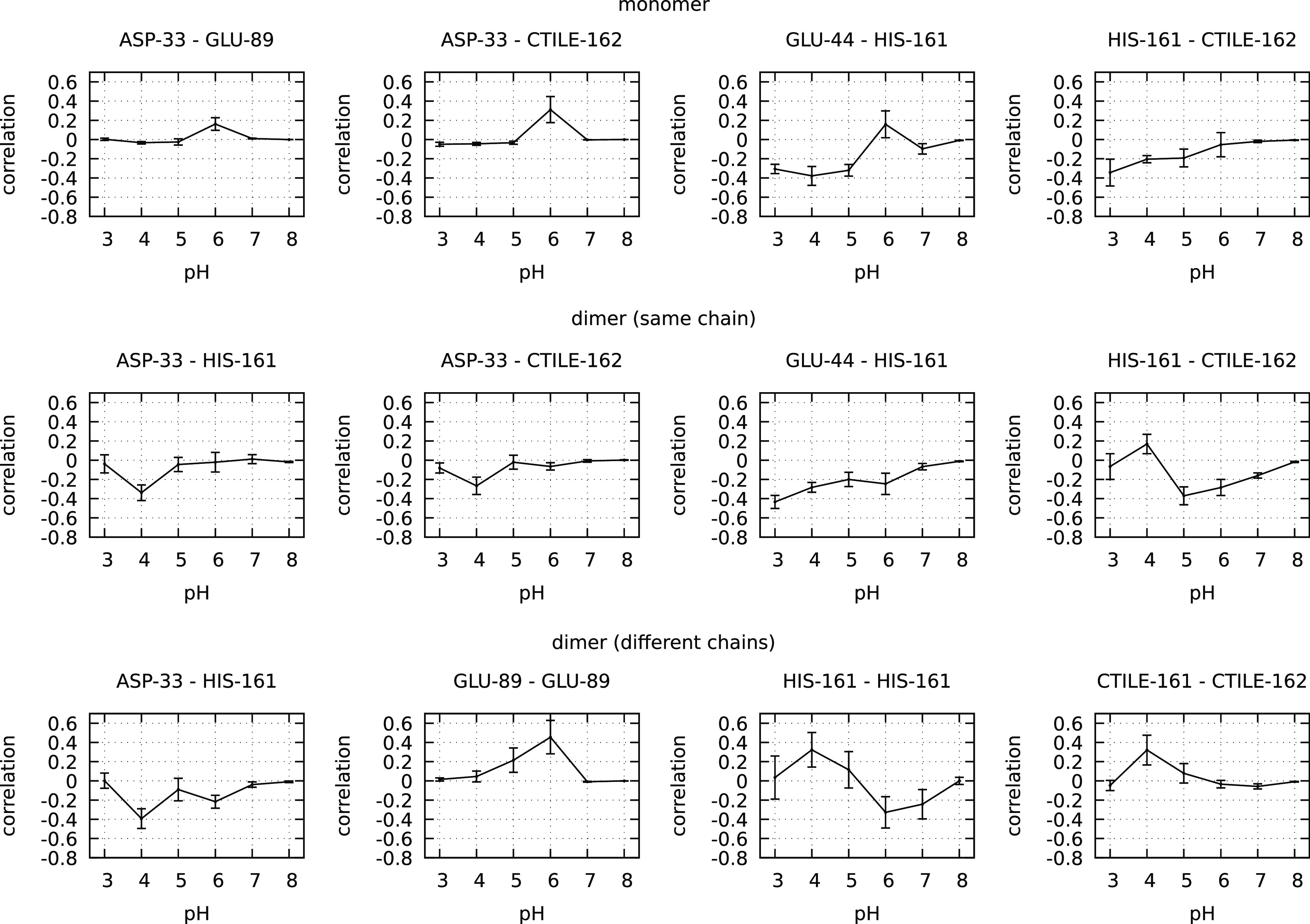
Caption remains unchanged.

**Figure 7:**
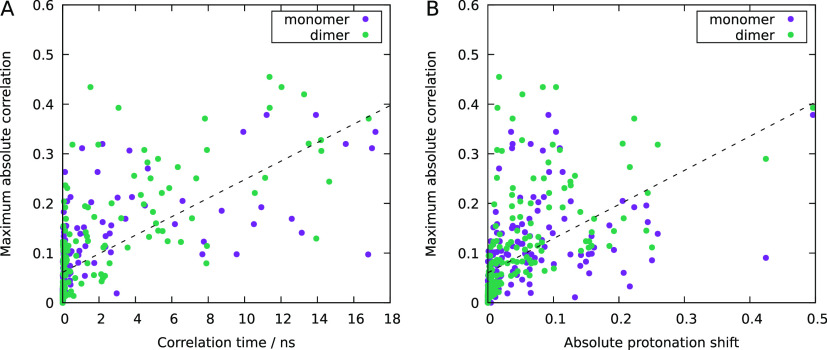
Caption remains unchanged.

## Supporting Information Available

Corrected Table S2 and Figure S10 (PDF)

