# Supplementary material for: Approach to Study pH-Dependent Protein Association Using Constant-pH Molecular Dynamics: Application to the Dimerization of β-Lactoglobulin
Source: J Chem Theory Comput. 2022 Feb 16;18(3):1982–2001. doi: 10.1021/acs.jctc.1c01187 (PMC9775224; doi:10.1021/acs.jctc.1c01187)
Supplement: Supplementary file 1 — ct1c01187_si_001.pdf [file ct1c01187_si_001.pdf]

Supporting Information: An approach to study pH-dependent protein association using constant-pH MD: application to the dimerization of β-lactoglobulin

Lucie da Rocha, António M. Baptista,* and Sara R. R. Campos*

Instituto de Tecnologia Química e Biológica António Xavier, Universidade Nova de Lisboa, Av. da República, 2780-157 Oeiras, Portugal

E-mail: baptista@itqb.unl.pt; scampos@itqb.unl.pt

Contents

List of Tables **S-2**

List of Figures **S-3**

1 Introduction **S-4**

2 Methods **S-5**

2.1 Spline interpolation S-7

2.2 Bootstrap Errors S-9

3 Results **S-11**

3.1 Equilibration Analyses S-11

3.2 Protonation Curves S-47

3.3 Dimerization Free Energy S-51

3.4 Protonation Correlations S-52

3.5 Electrostatic Complementarity S-60

3.6 Dimer Configurations S-62

3.7 EF Loop S-65

References **S-66**

List of Tables

S1 Average BLG net charge in preliminary simulations without ions and calculated numbers of Na^+ and Cl^- ions. S-5

S2 Average system net charge in the simulations with ions. S-6

S3 Experimental BLG dimerization free energies. S-51

List of Figures

S1 Dependence of the fraction of dimer as a function of BLG concentration. . . S-4
S2 Simulation time series of the RMSD of the monomer and each dimer chain. . S-11
S3 Simulation time series of the secondary structure fraction. S-12
S4 Simulation time series of the contact surface area between the two dimer partners. S-12
S5 Simulation time series of the distance between the centers of mass of the two dimer partners. S-13
S6 Simulation time series of the cumulative average of the total protonation. . . S-14
S7 Simulation time series of the cumulative average of the protonation of BLG sites. S-15
S8 Identification of dissociated structures in the dimer simulations. S-46
S9 Individual protonation curves of BLG sites in the monomer and dimer. . . . S-47
S10 Networks of protonation correlations in BLG monomer. S-52
S11 Networks of protonation correlations in BLG dimer. S-55
S12 BLG structure showing the network of the protonation correlations in the dimer at pH 5. . . . . S-58
S13 Correlation time of the proton occupancy of the BLG sites in the monomer and dimer. S-59
S14 Density contours of 200 mM for the Na^+ and Cl^- ions in the dimer simulations S-60
S15 Density contours of 150 mM for the Na^+ and Cl^- ions in the monomer simulations S-61
S16 Dihedral angles between the helices and β-strands at the interface. S-62
S17 Scatter plot of the distance between the COM vs PC1. . . . . S-63
S18 Scatter plots of the interface dihedral angles vs PC1. . . . . S-64
S19 Average values of the first PC of the EF loop PCA. S-65

1 Introduction

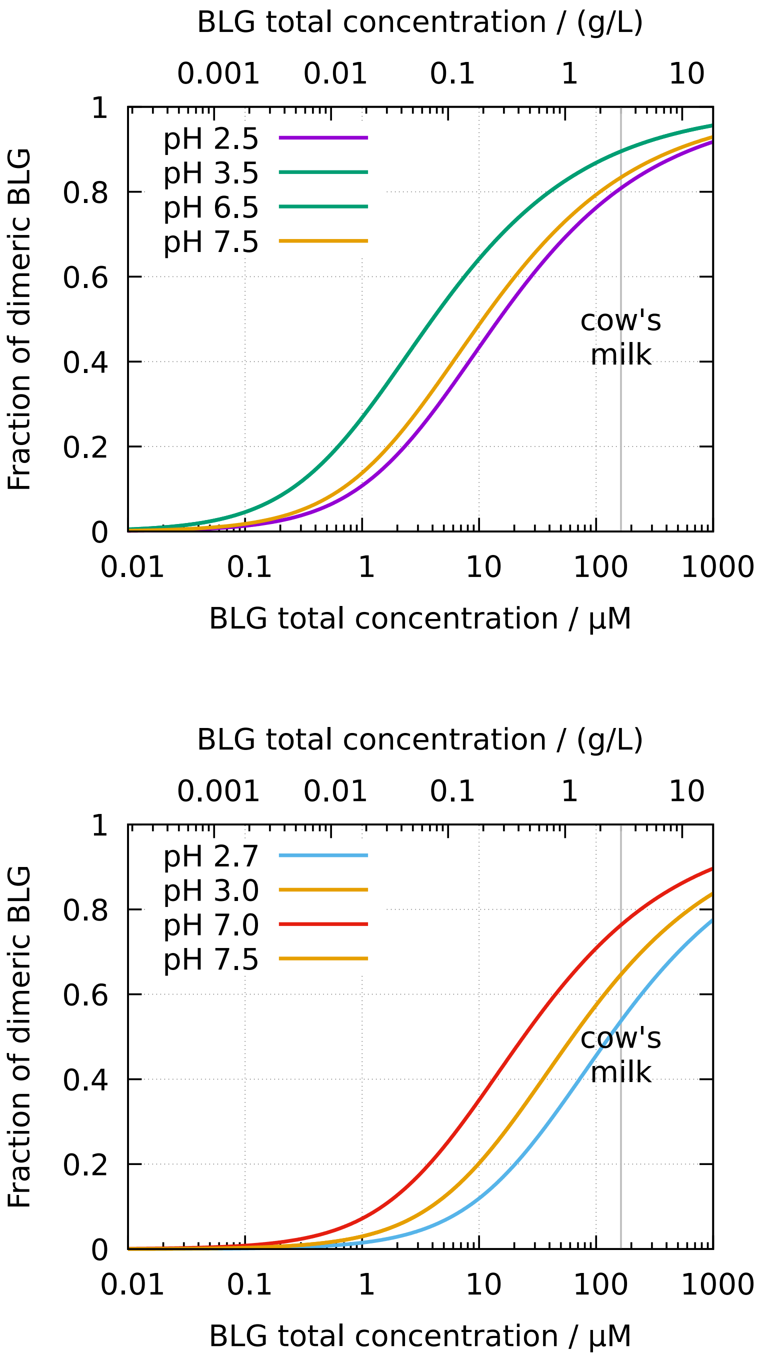

Figure S1: Dependence of the fraction of dimer as a function of BLG concentration for different pH values. The fractions were computed from the dissociation constants reported in ref. S1 (**top** plot) and in refs. S2–S5 (**bottom** plot), showing the variation between different studies. The concentration of BLG in milk is indicated as a dashed line.

2 Methods

Table S1: Average BLG net charge in preliminary simulations without ions and calculated numbers of Na^+ and Cl^- ions necessary to ensure approximate neutrality at ionic strength of 0.1 M. Simulations started with the EF loop in an open (PDB code: 1BSY) or closed (PDB code: 3BLG) configuration were treated separately.

	Monomer							
	Preliminary simulations				# ions to be added			
	net charge		Total time (ns)		Na^+	Cl^-	Na^+	Cl^-
pH	open	closed	open	closed	open		closed	
3	15.93	15.86	200	200	10	26	10	25
4	8.54	8.17	200	200	14	23	13	22
5	−0.24	−0.32	200	200	18	18	18	17
6	−5.79	−5.39	200	200	21	15	21	15
7	−8.91	−8.77	200	200	22	14	22	13
8	−9.73	−9.65	200	200	23	13	22	13

	Dimer							
	Preliminary simulations				# ions to be added			
	net charge		Total time (ns)		Na^+	Cl^-	Na^+	Cl^-
pH	open	closed	open	closed	open		closed	
3	28.34	28.31	200	173	56	85	54	82
4	15.69	16.16	200	200	63	78	60	76
5	1.11	1.80	200	200	70	71	67	68
6	−10.07	−9.15	200	200	75	65	73	63
7	−16.53	−15.24	87	157	79	62	76	60
8	−19.08	−18.97	192	200	80	61	77	58

Table S2: Average system net charge in the simulations with ions.

	net charge	
pH	monomer	dimer
3	−0.43	−0.05
4	0.05	−0.23
5	0.70	0.54
6	0.59	0.16
7	0.41	−0.31
8	0.16	−0.62

2.1 Spline interpolation

Given a set of n points $(x_1, y_1), (x_2, y_2), \ldots, (x_n, y_n)$, with $x_1 < x_2 < \cdots < x_n$, and the associated slopes $y'_1, y'_2, \ldots, y'_n$, we can define an interpolating Hermite cubic spline $f(x)$ so that, for $x \in [x_i, x_{i+1}]$, we have (see section 5.3 of Ref. S6)

$$f(x) = h_i(x) = \sum_{k=0}^{3} a_{ik}(x - x_i)^k \ ,$$

with

$$\begin{aligned}
a_{i0} &= y_i \\
a_{i1} &= y'_i \\
a_{i2} &= \frac{3(y_{i+1} - y_i)/(x_{i+1} - x_i) - 2y'_i - y'_{i+1}}{(x_{i+1} - x_i)} \\
a_{i3} &= \frac{y'_i + y'_{i+1} - 2(y_{i+1} - y_i)/(x_{i+1} - x_i)}{(x_{i+1} - x_i)^2} \ .
\end{aligned}$$

This ensures that $h_{i-1}(x_i) = h_i(x_i) = y_i$ and $h'_{i-1}(x_i) = h'_i(x_i) = y'_i$ for $2 \le i \le n-1$, meaning that $f(x)$ and $f'(x)$ are continuous in $[x_1, x_n]$.

An analytic expression for the spline integral can be obtained by summing the contributions from each interval. Thus, the integral of $f(x)$ from the initial point x_1 to a point $x \in [x_k, x_{k+1}]$ can be written as

$$\int_{x_1}^{x} f(t)\,\mathrm{d}t = \sum_{i=1}^{k-1} g_i(x_{i+1}) + g_k(x) \ ,$$

where

$$g_i(x) = \int_{x_i}^{x} h_i(t)\,\mathrm{d}t = \sum_{k=0}^{3} \frac{a_{ik}}{k+1}\,(x - x_i)^{k+1}$$

is the interval-specific integral of h_i between x_i and an arbitrary value $x \in [x_i, x_{i+1}]$. The integral of $f(x)$ is then a piecewise quartic polynomial.

As discussed in section 2.4 of the main article, we are here interested in the case where the x_i are the simulated pH values, the y_i are the total or individual average proton occupancies, and the y'_i are the slopes obtained from equation 6 or 7.

2.2 Bootstrap Errors

As discussed in section 2 of the main manuscript, r simulation replicates are run at each pH ($r = 4$ for the monomer and $r = 8$ for the dimer), each of which produces a time series of coordinates and protonation states. These r time series are then collected into a single set that is used to compute the intended quantities.

The statistical errors of all protonation-derived quantities were computed using a bootstrap method[S7] performed over replicates, which avoids the need to account for the temporal correlations present within each replicate-specific time series. Furthermore, since some quantities are explicitly derived from the pH dependency (e.g., the pK_a of a site is derived by fitting a Hill curve to its pH-dependent average protonation), each resampled data set is generated in way that includes that pH dependency. More exactly, each resampled data set is obtained by randomly selecting r new replicates from the original ones, at each of the six simulated pH values (which selects one data set from among the $\binom{2r-1}{r}^6$ different ones corresponding to this combinatorial procedure). This was repeated 1000 times for the monomer and for the dimer simulations, and the resulting resampled data sets were used to estimate the statistical errors of the protonation-derived quantities using the usual bootstrap plugin rationale,[S7] as explained below in more detail.

To estimate the error of the pK_a and Hill coefficient (h) of each site, shown in Table 1, a Hill curve was fitted to the pH-dependent average protonation obtained from each of the 1000 resampled data sets. The standard error of each parameter, pK_a and h, was then computed as the standard deviation of the set of 1000 parameter values thus produced.[S7]

Error bounds for each $\Delta\Delta G_i$ curve were computed from the resampled data sets as follows:

1. A spline curve interpolating the average protonations was derived from each of the 1000 resampled data sets, as described in section 2.4 of the paper.

2. Each of the resulting 1000 monomer/dimer curve pairs was then used to compute a

continuous $\Delta\Delta G_i$ curve using eq. 5.

3. Each of these 1000 $\Delta\Delta G_i$ curves was vertically least-square fitted to the original (non-resampled) curve.

4. At finely-spaced pH values a standard deviation was computed from the 1000 corresponding $\Delta\Delta G_i$ values, producing an essentially continuous standard error envelope for the original (non-resampled) curve, as shown in Figure 5.

The vertical fitting follows from the fact that, as noted in section 2.4 of the paper, each integration produces only a curve shape; for example, choosing the same arbitrary $\mathrm{pH}_{\mathrm{ref}}$ for each of the 1000 resamples would give $\Delta\Delta G_i(\mathrm{pH}_{\mathrm{ref}}) = 0$ for all of them and, consequently, an error of zero at that arbitrary pH value, which is absurd. The procedure just described was also used to compute the standard error envelope of the global $\Delta\Delta G$ curve, shown in Figure 4.

For each titratable site, the error of its average protonation at each simulated pH, shown in the plots of Figure S9, was obtained by computing a 68% confidence interval from the 1000 average protonations computed from the resampled data sets, using the percentile method.[S7] This gives an interval whose magnitude should be roughly similar to the symmetric one of plus/minus one standard error, but avoids error bars that go below zero or above one.

For the correlation between a pair of sites, the standard error at each simulated pH value, shown in the plots of Figure 6, was computed as the standard deviation of the set of 1000 correlation values obtained from the resampled data sets.

3 Results

3.1 Equilibration Analyses

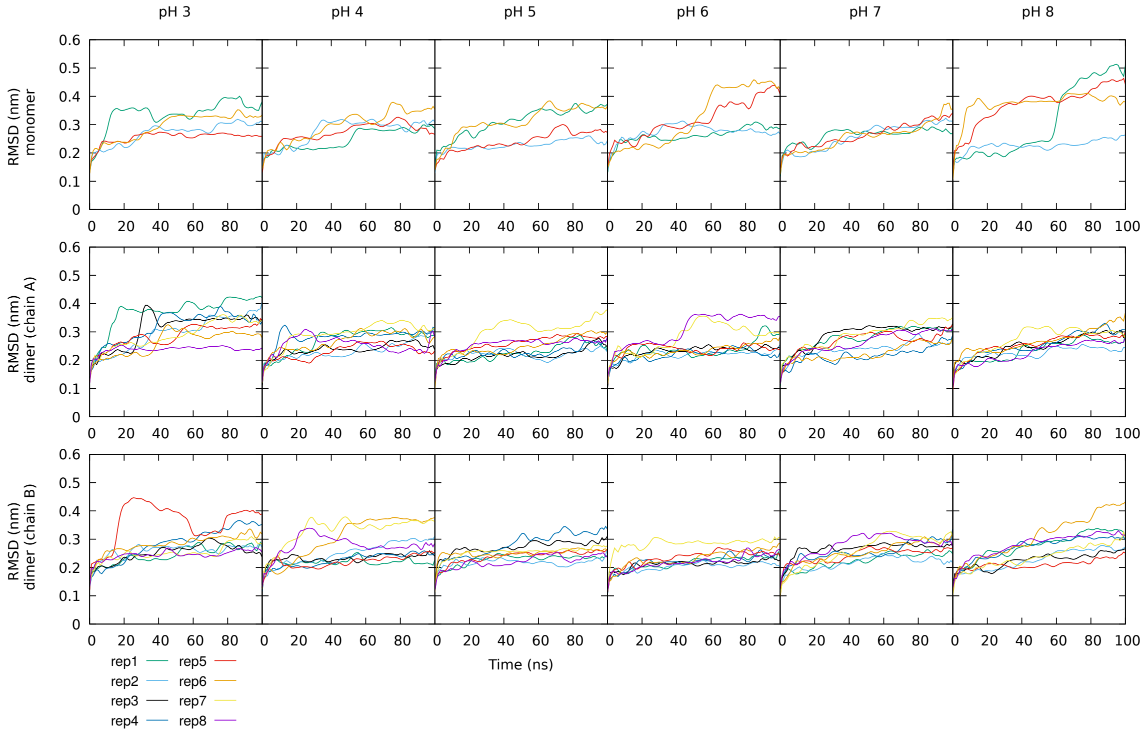

Figure S2: Simulation time series of the RMSD of the backbone atoms relative to the initial structure for the monomer and each dimer chain (chain A and chain B). Replicates 1–4 were started from an open conformation and 5–8 from a closed one.

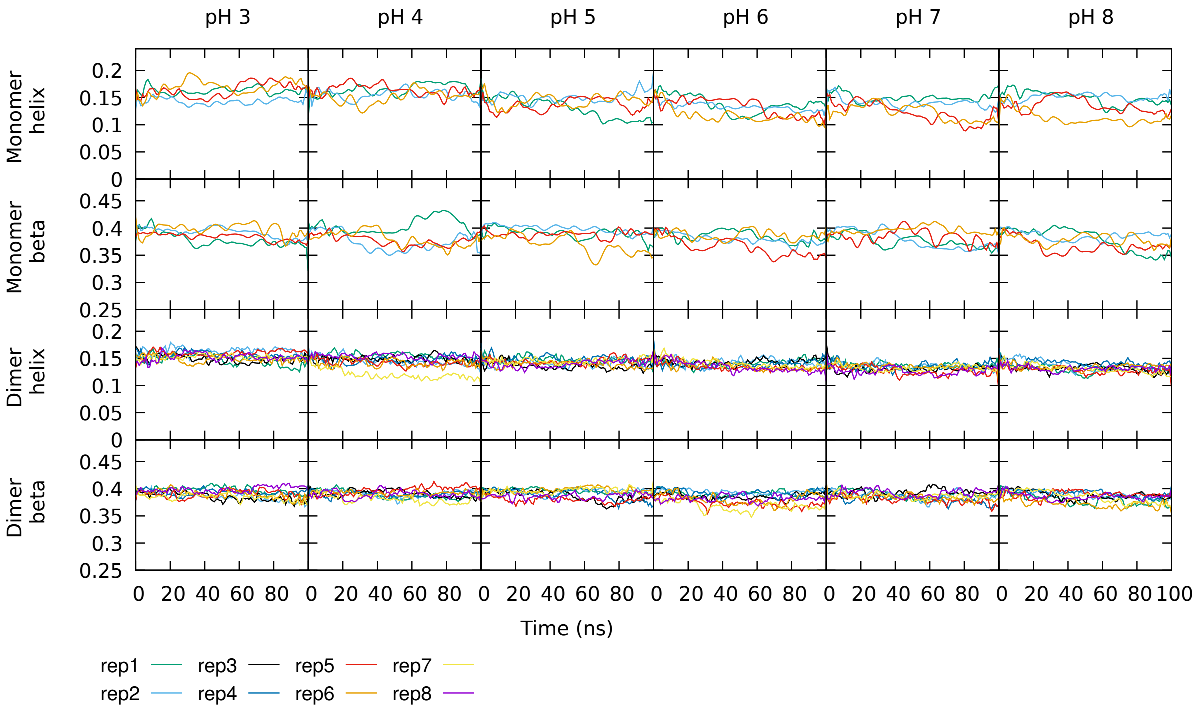

Figure S3: Simulation time series of the secondary structure fraction. The secondary structure of the protein was assigned using the DSSP criterion defined by Kabsch and Sander.[S8] The different types of helix are classified as a single helix content and the β-bridge and β-sheet are reported as a single β-structure content. Replicates 1–4 were started from an open conformation and 5–8 from a closed one.

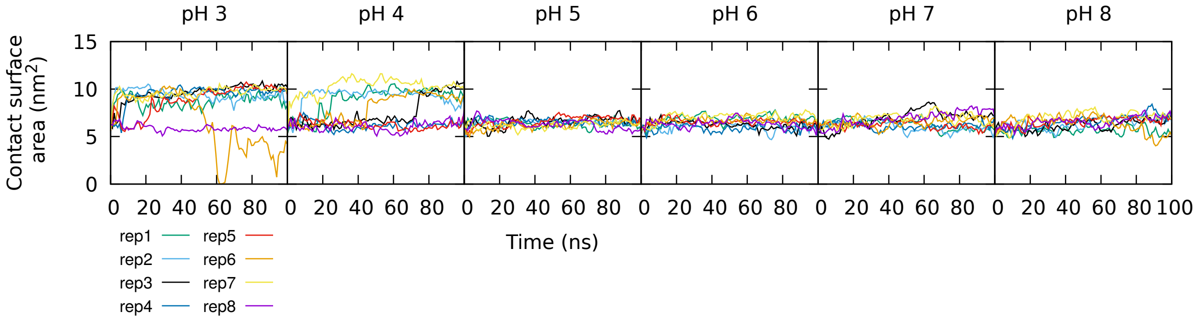

Figure S4: Simulation time series of the contact surface area between the two dimer partners. Replicates 1–4 were started from an open conformation and 5–8 from a closed one.

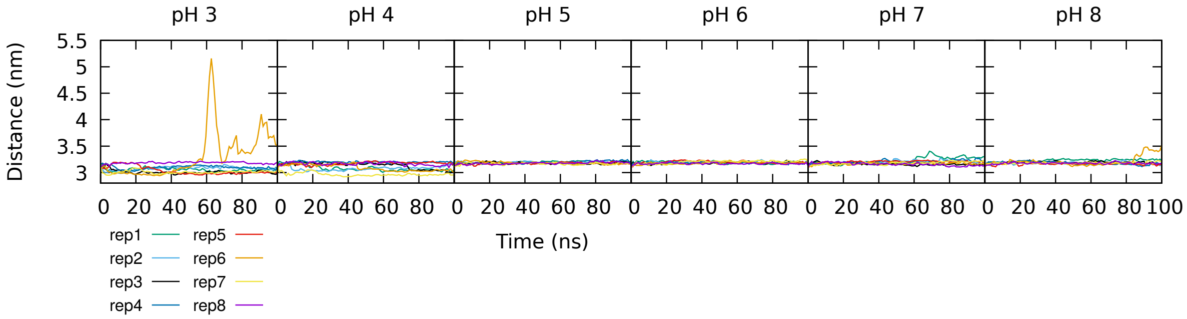

Figure S5: Simulation time series of the distance between the centers of mass of the two dimer partners. Replicates 1–4 were started from an open conformation and 5–8 from a closed one.

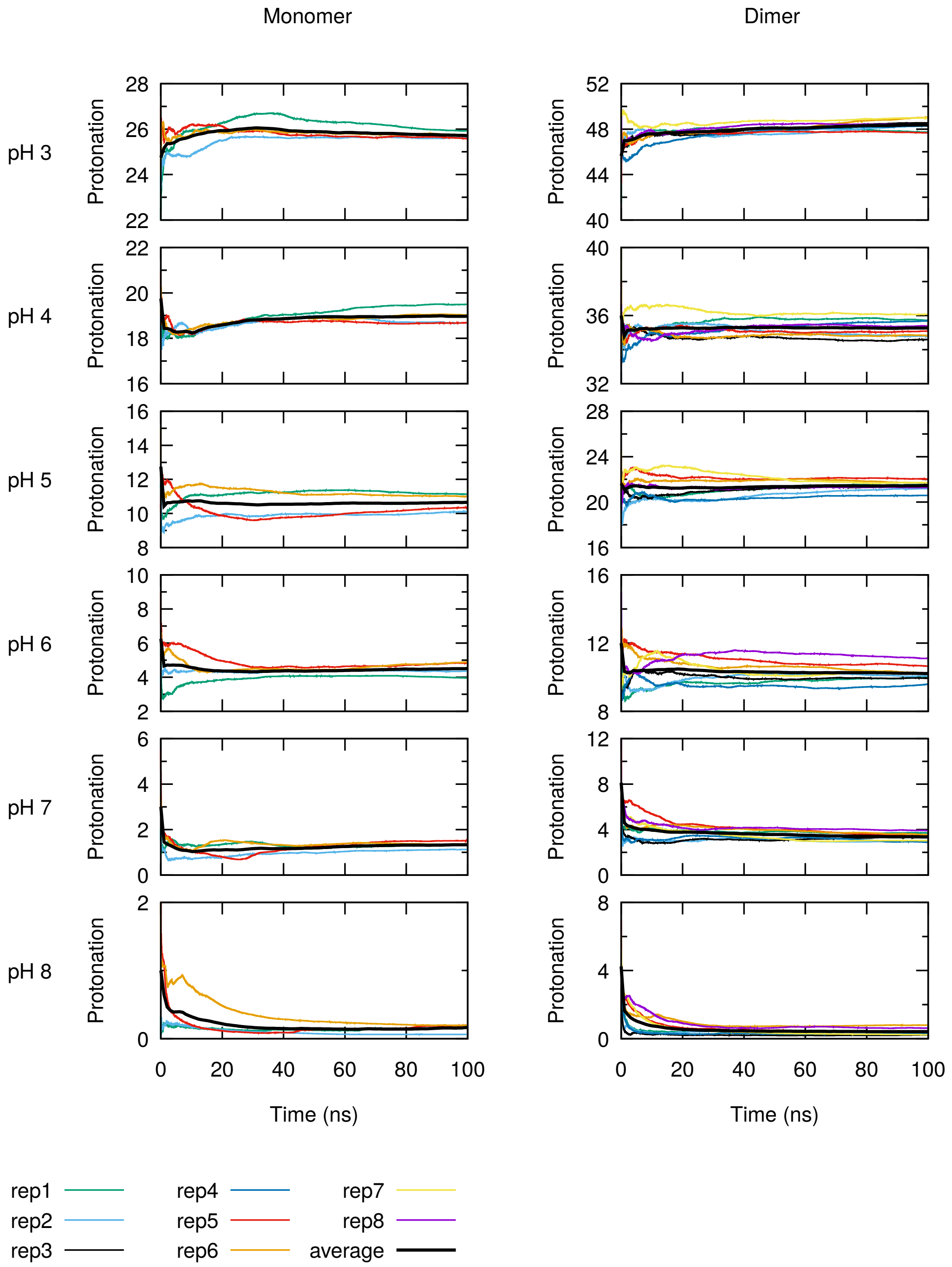

Figure S6: Simulation time series of the cumulative average of the total protonation. Replicates 1–4 were started from an open conformation and 5–8 from a closed one. The black thick curve corresponds to the average over all replicates.

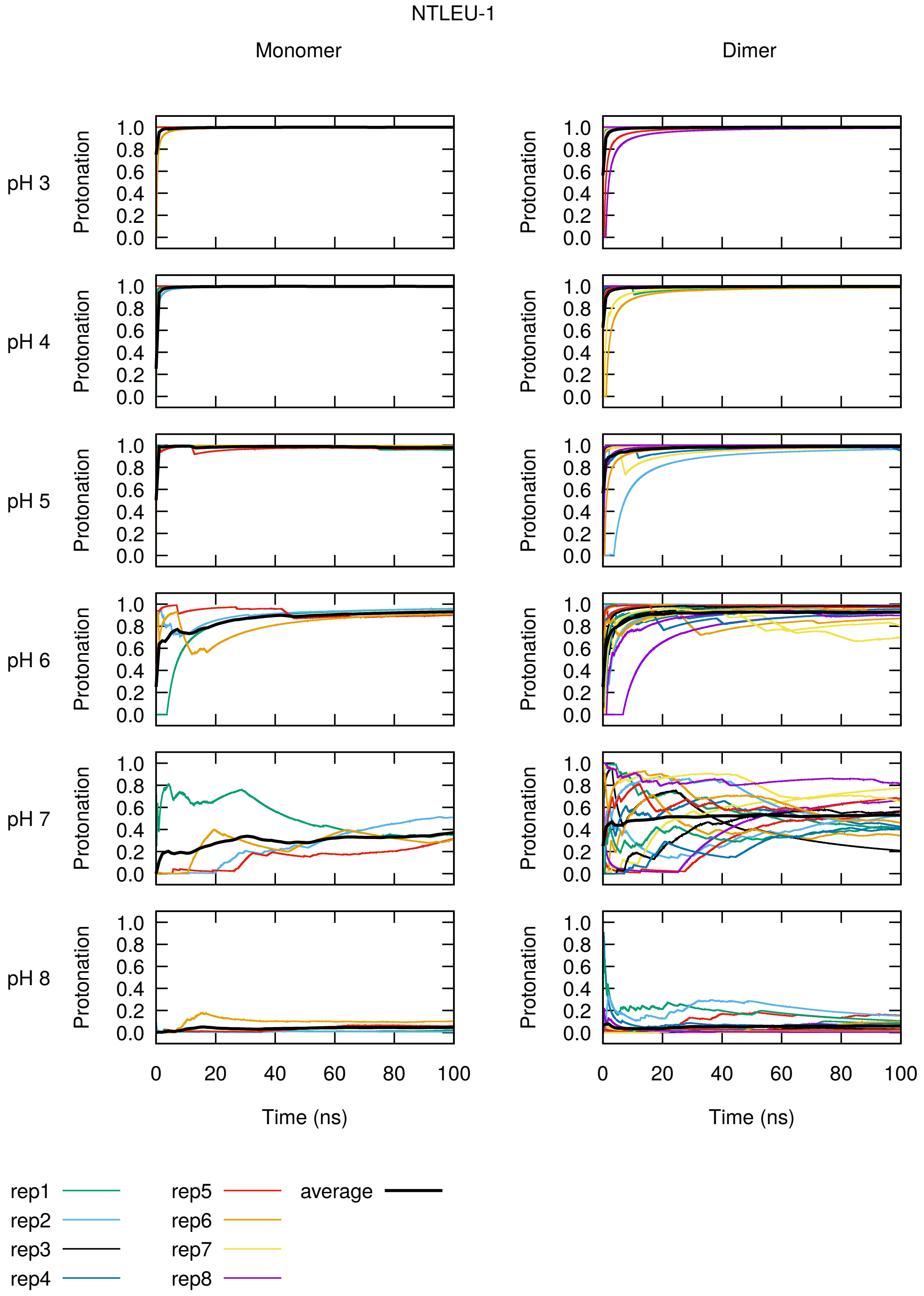

Figure S7: Simulation time series of the cumulative average of the protonation of BLG sites. Replicates 1–4 were started from an open conformation and 5–8 from a closed one. The black thick curve corresponds to the average over all replicates.

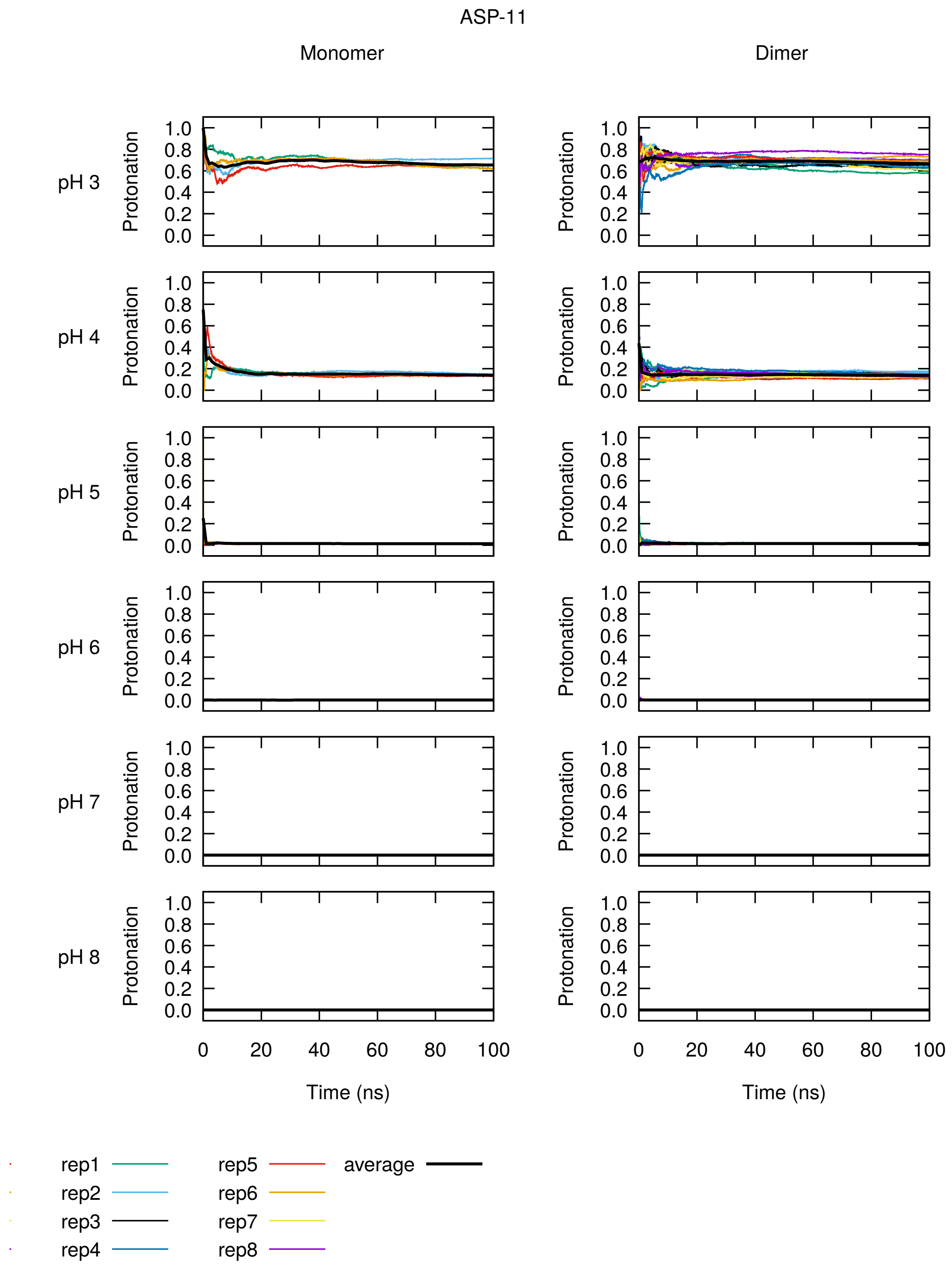

Figure S7: (continued, part 2)

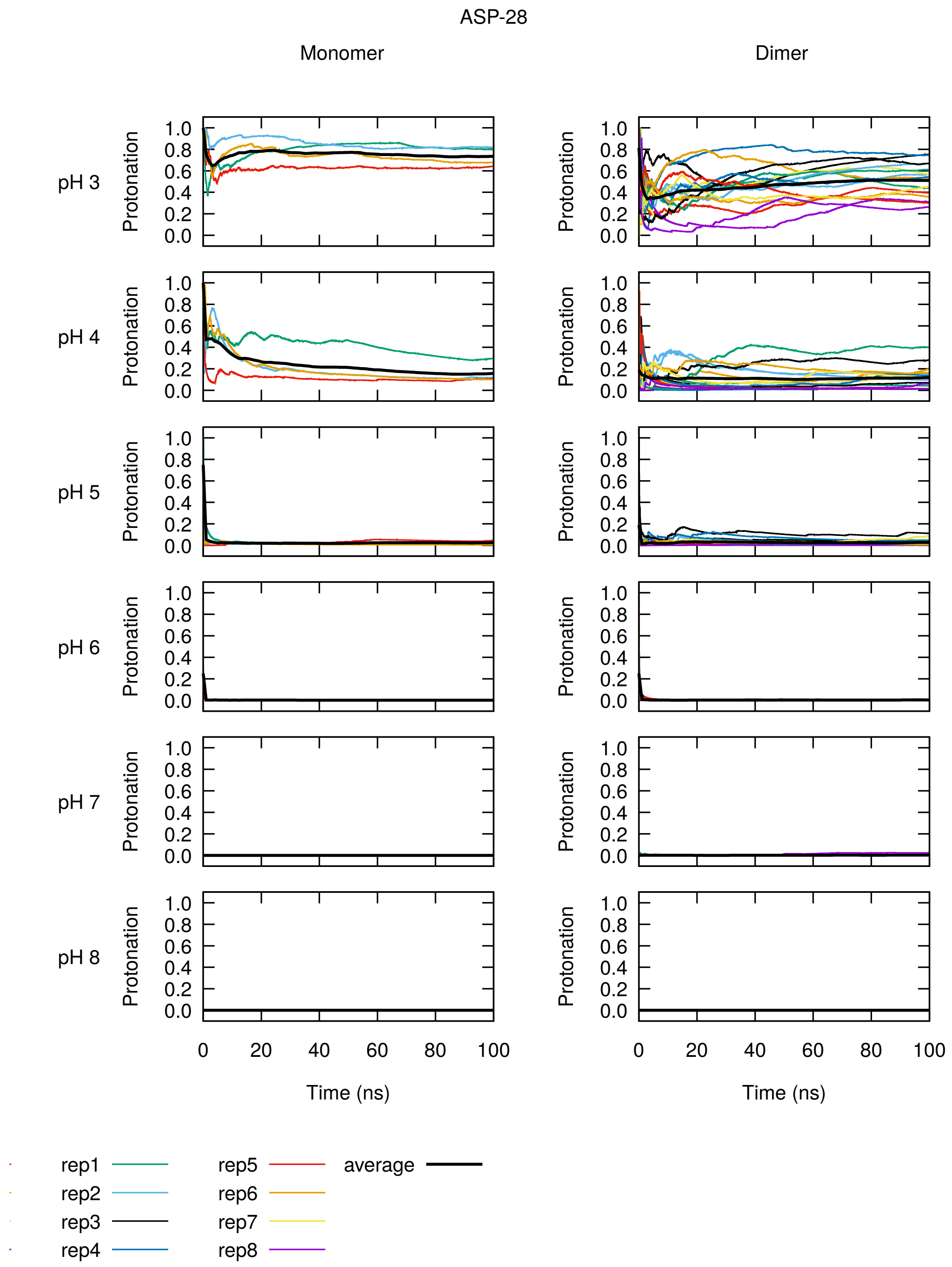

Figure S7: (continued, part 3)

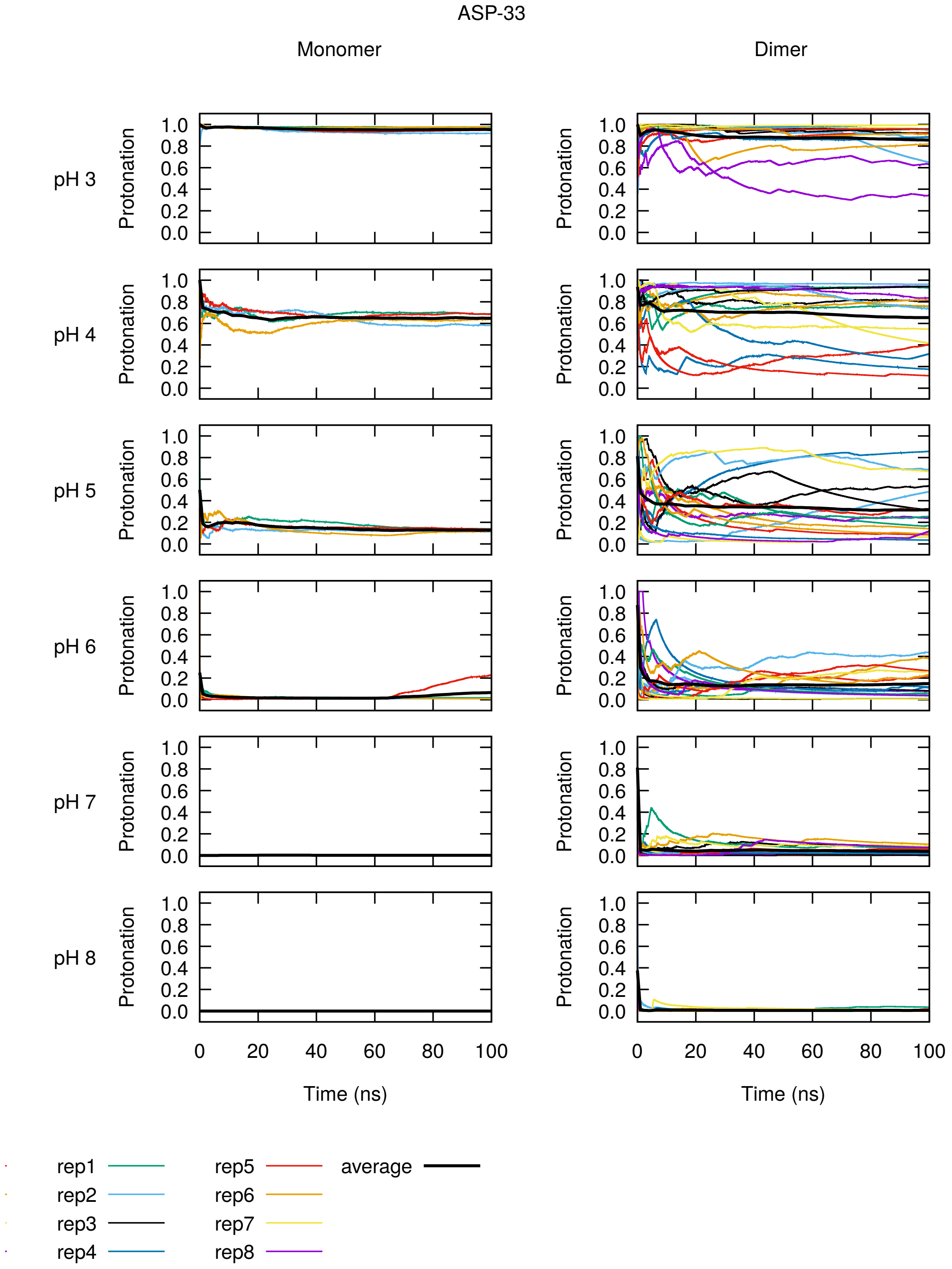

Figure S7: (continued, part 4)

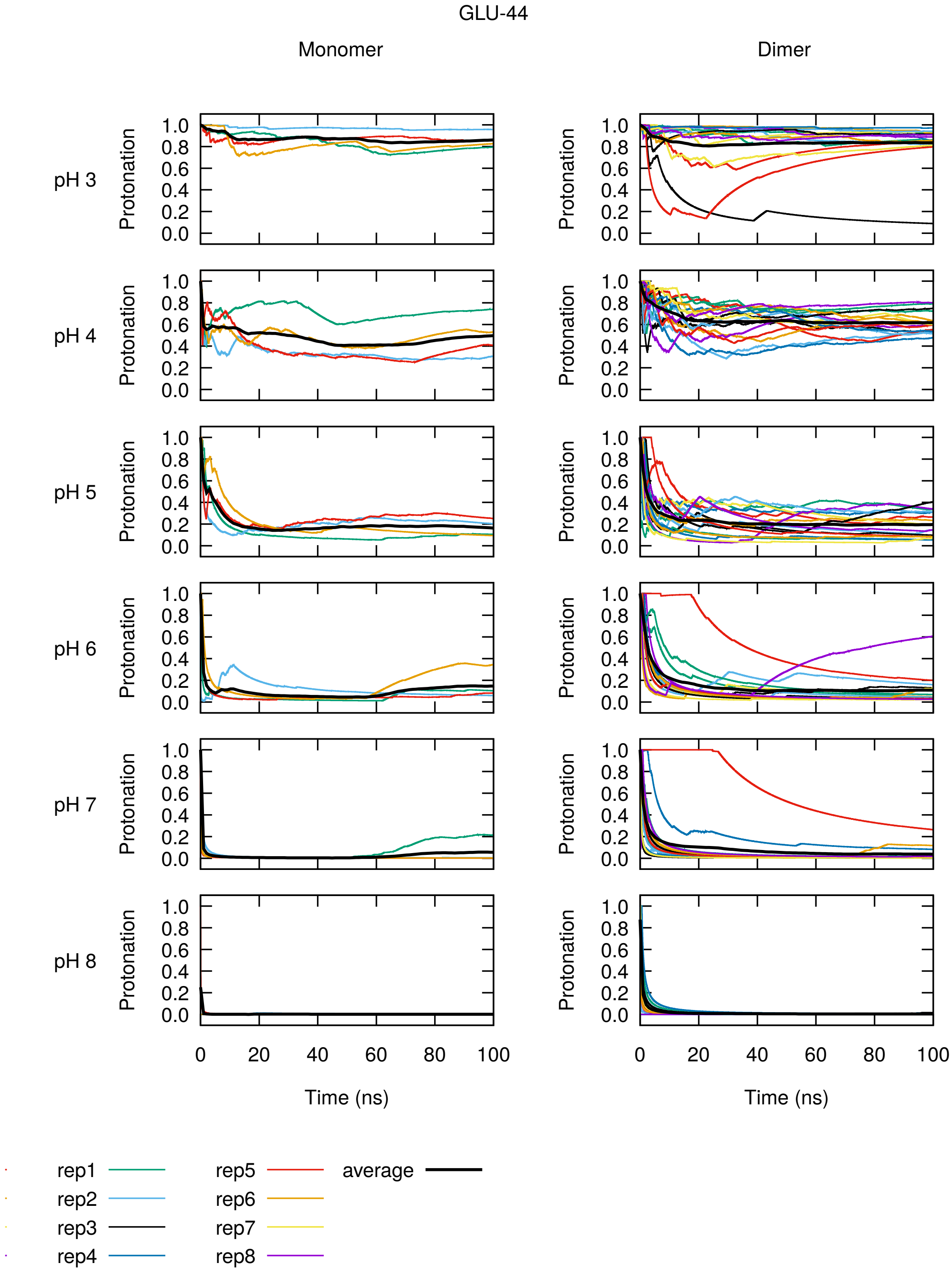

Figure S7: (continued, part 5)

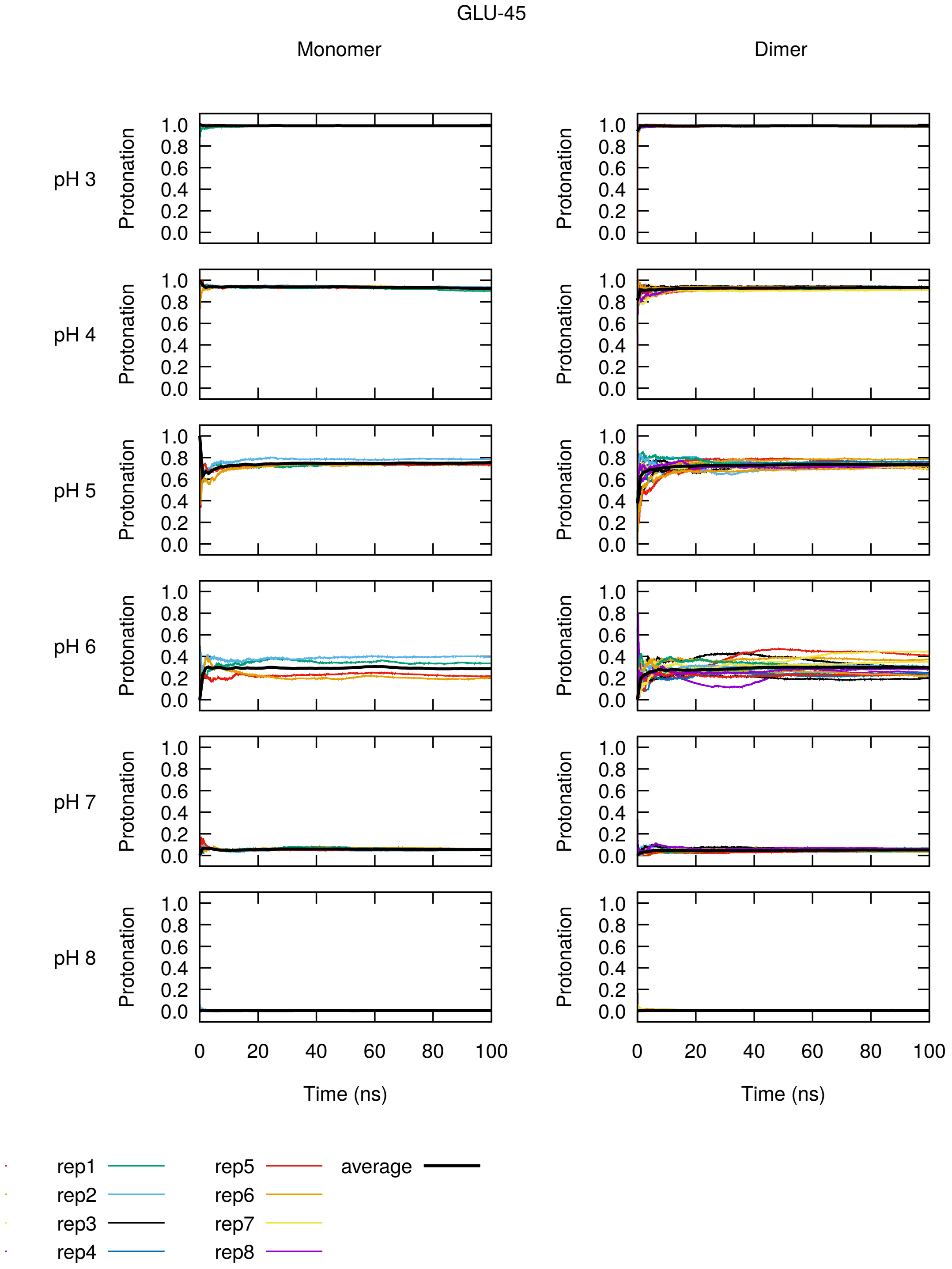

Figure S7: (continued, part 6)

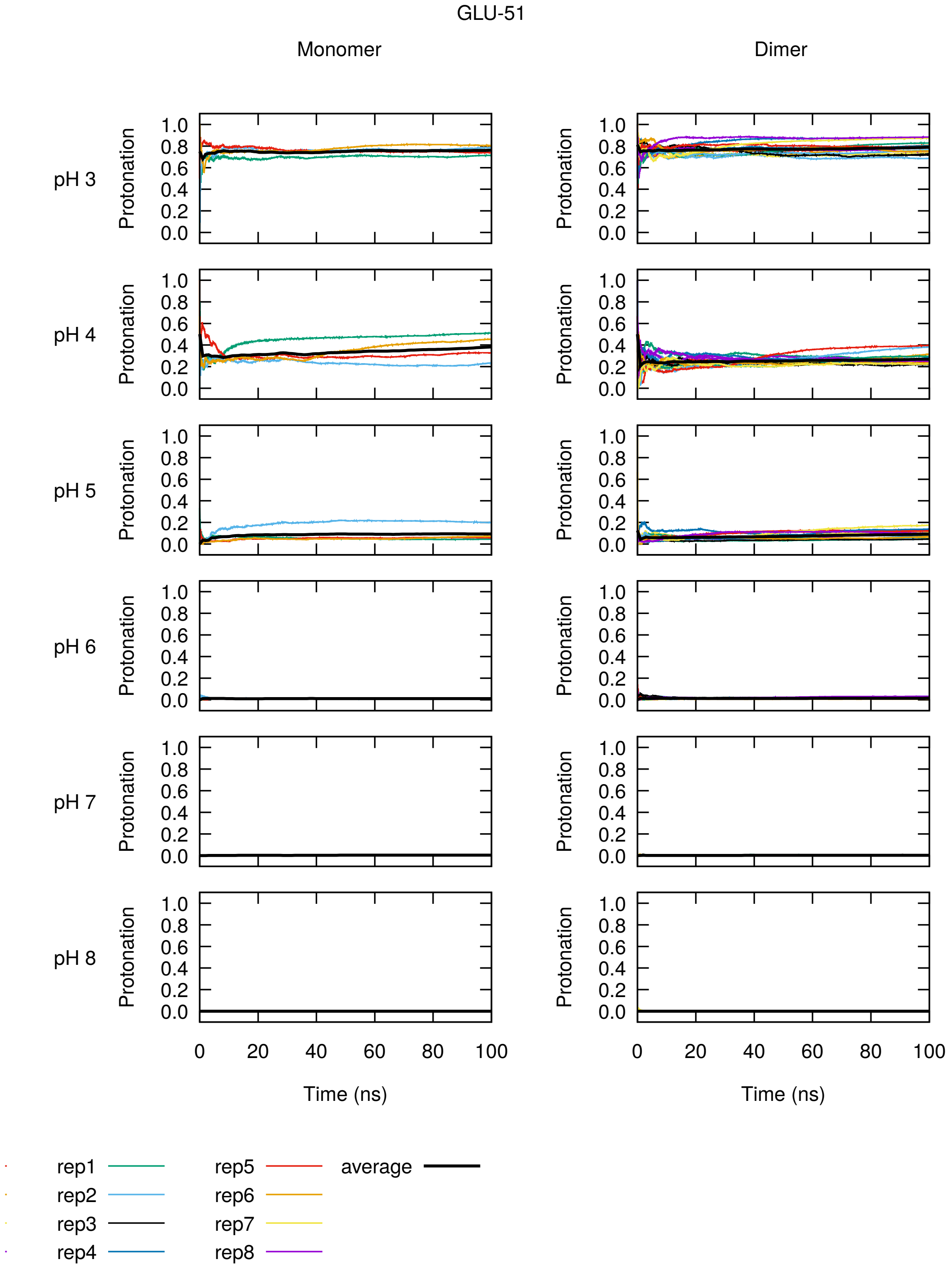

Figure S7: (continued, part 7)

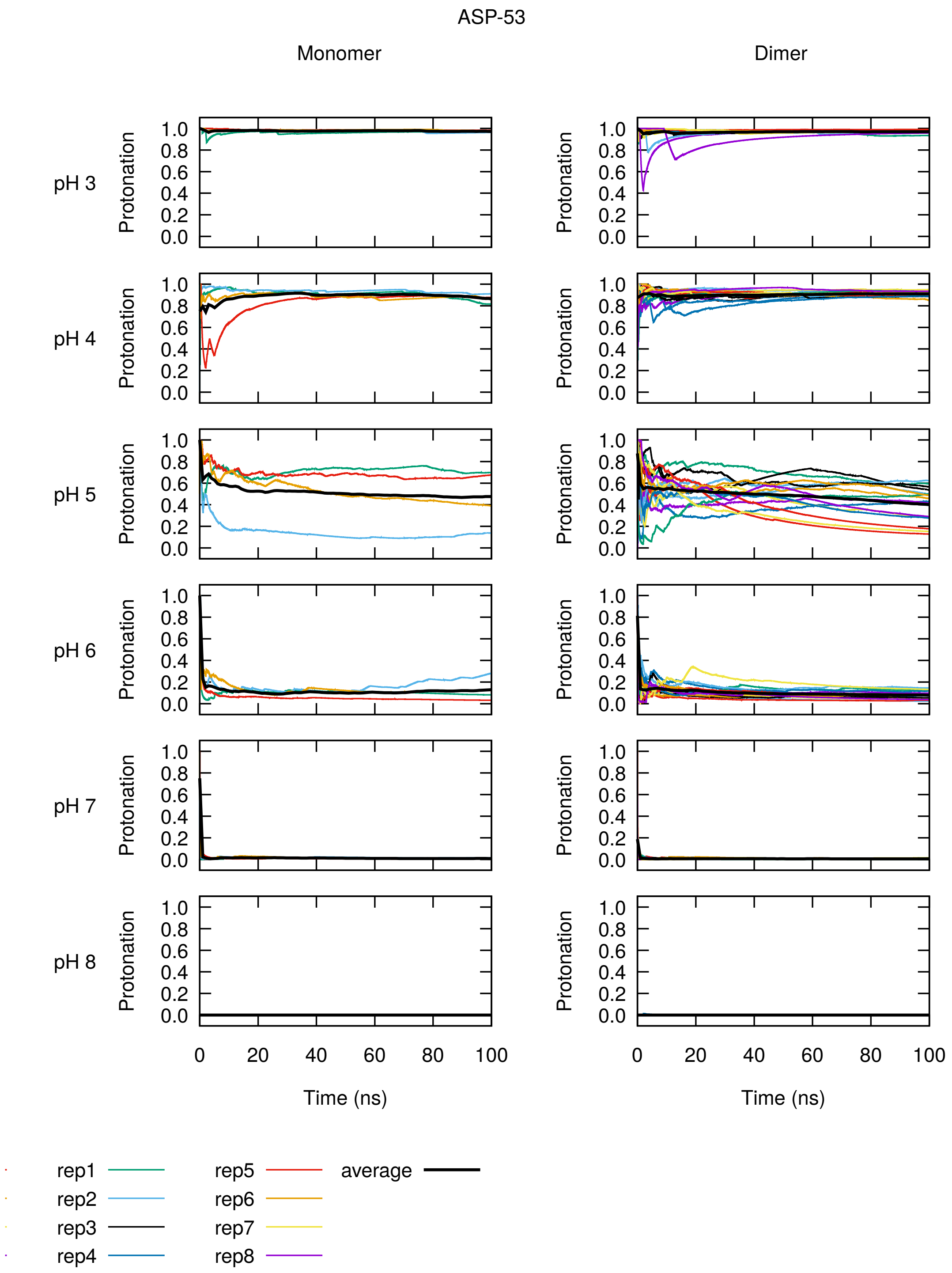

Figure S7: (continued, part 8)

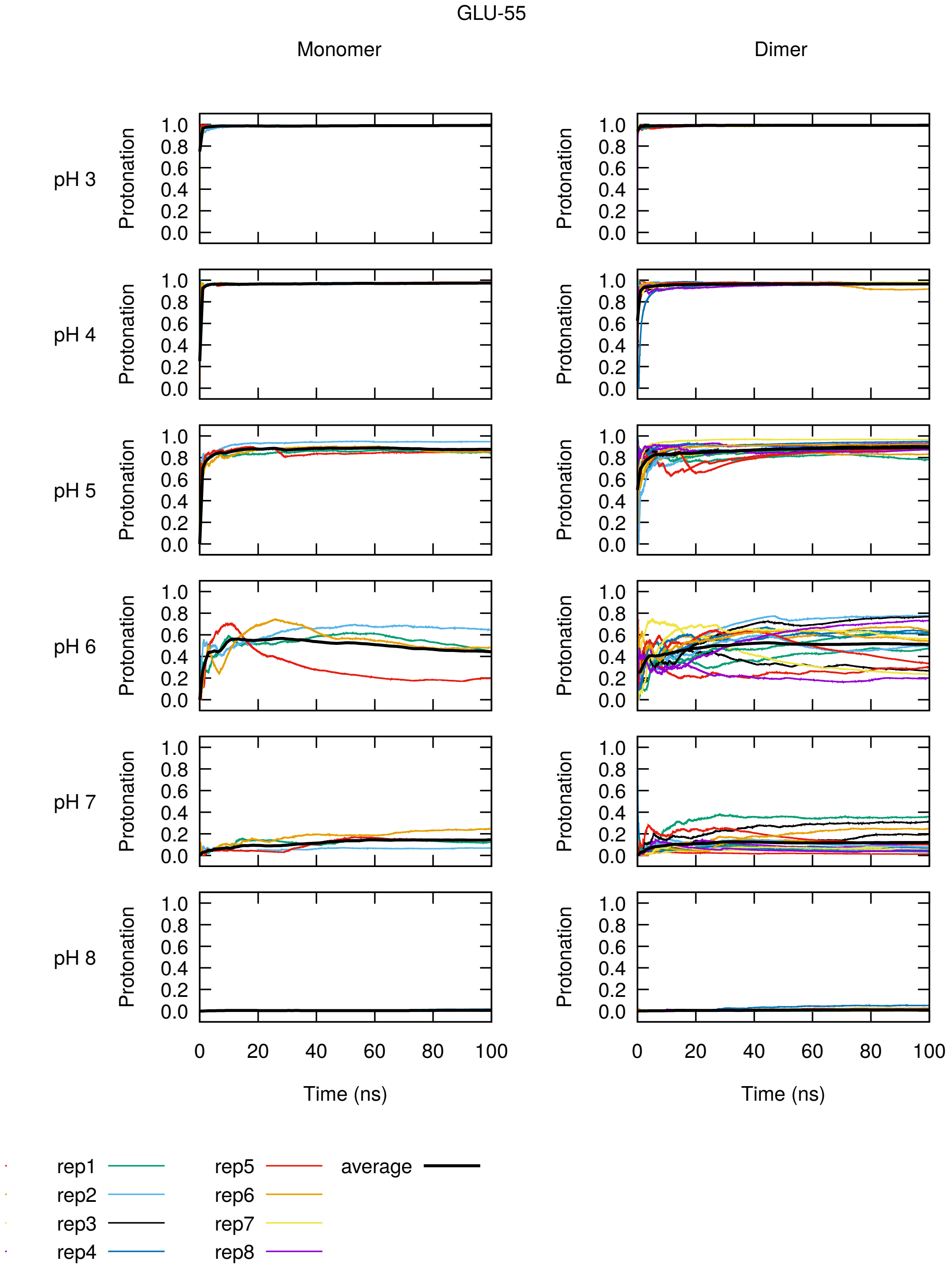

Figure S7: (continued, part 9)

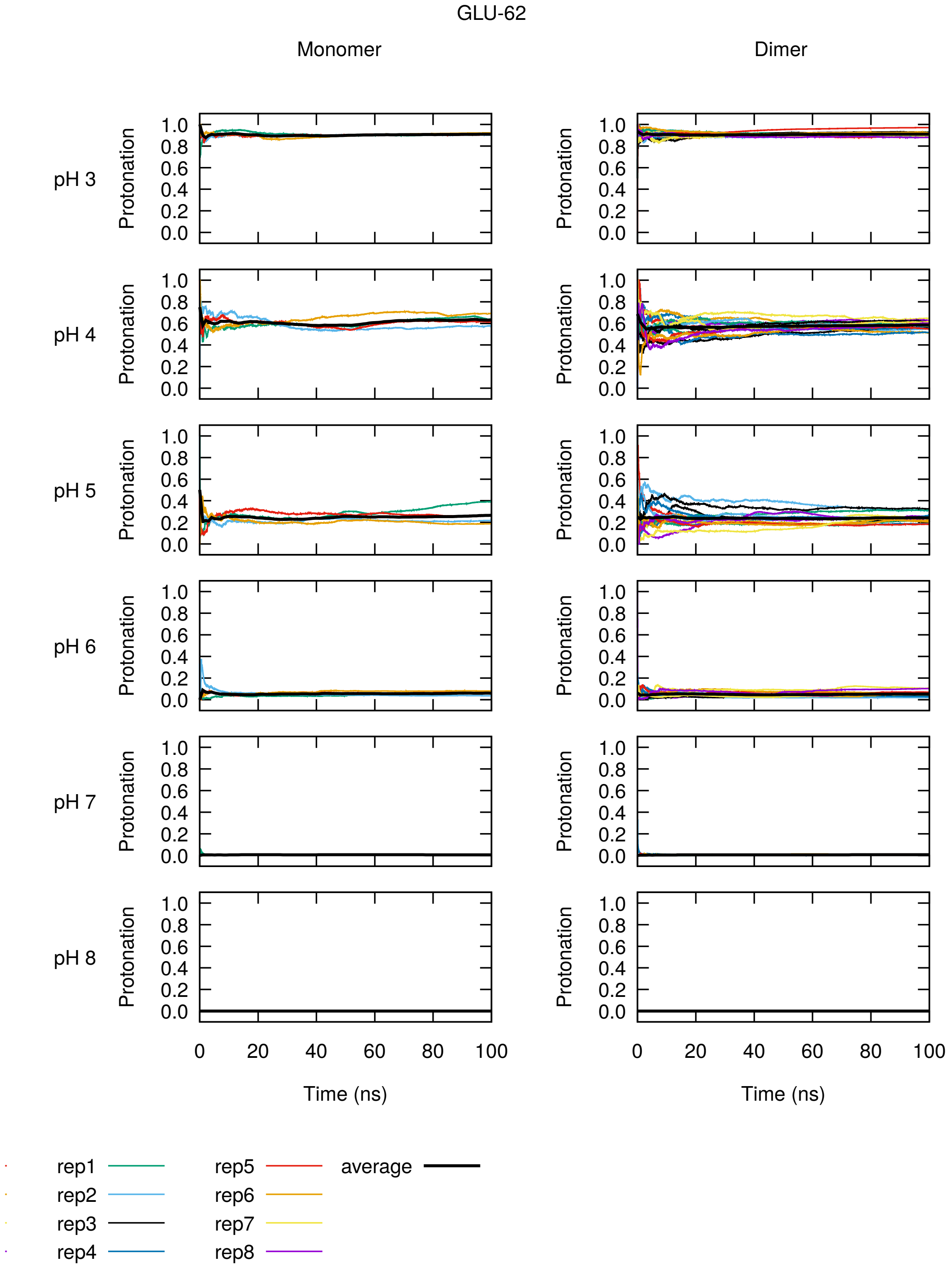

Figure S7: (continued, part 10)

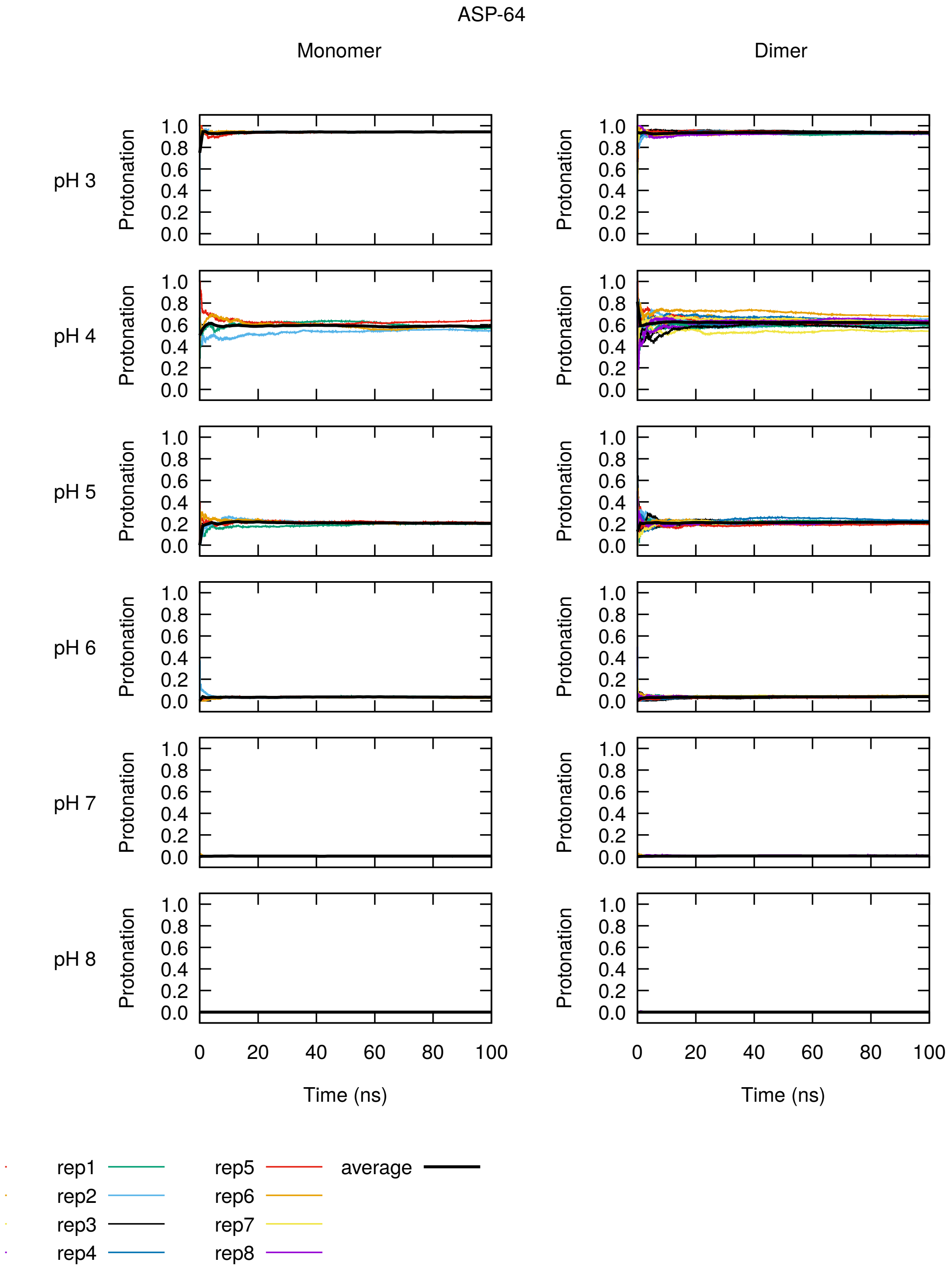

Figure S7: (continued, part 11)

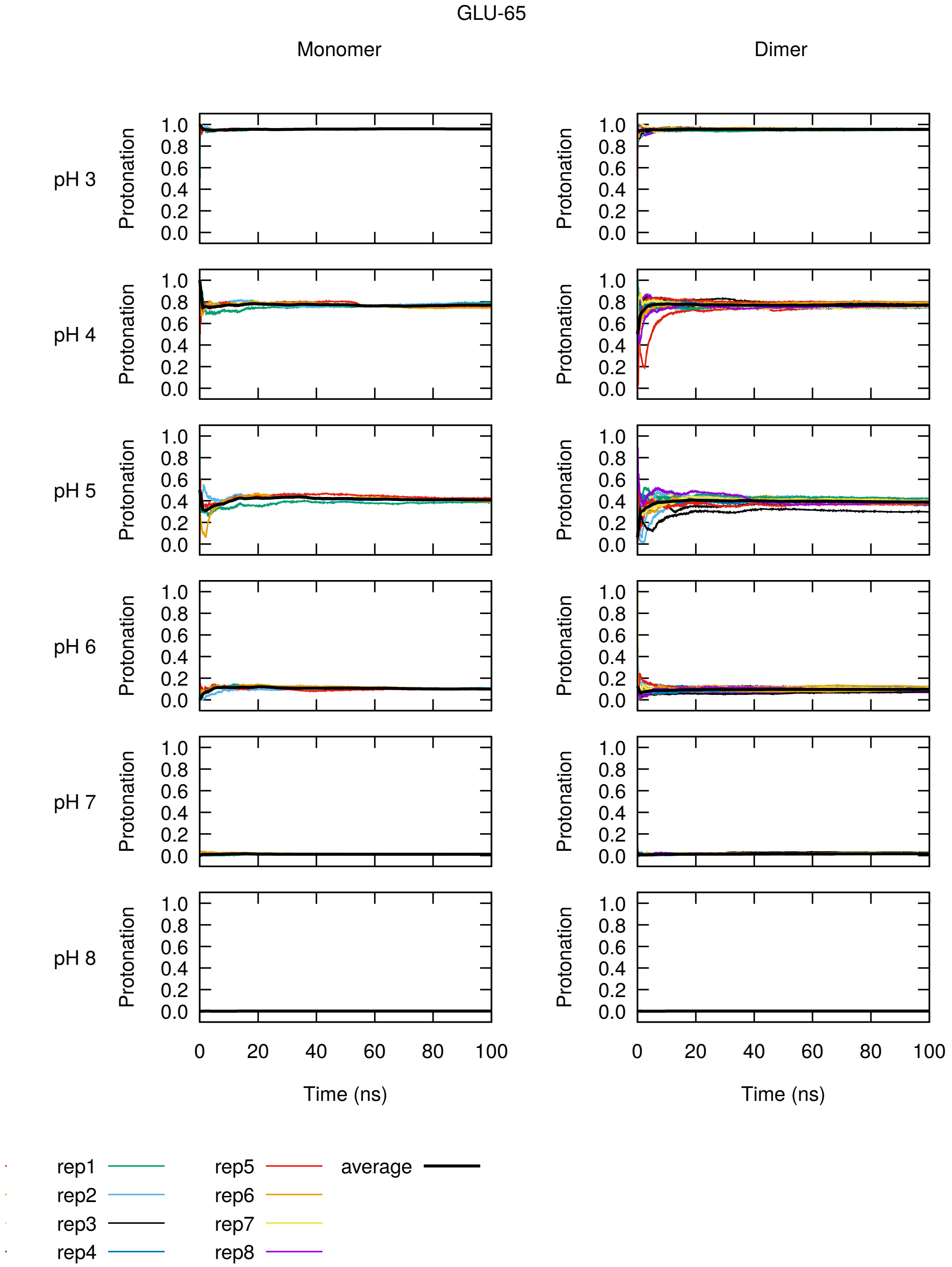

Figure S7: (continued, part 12)

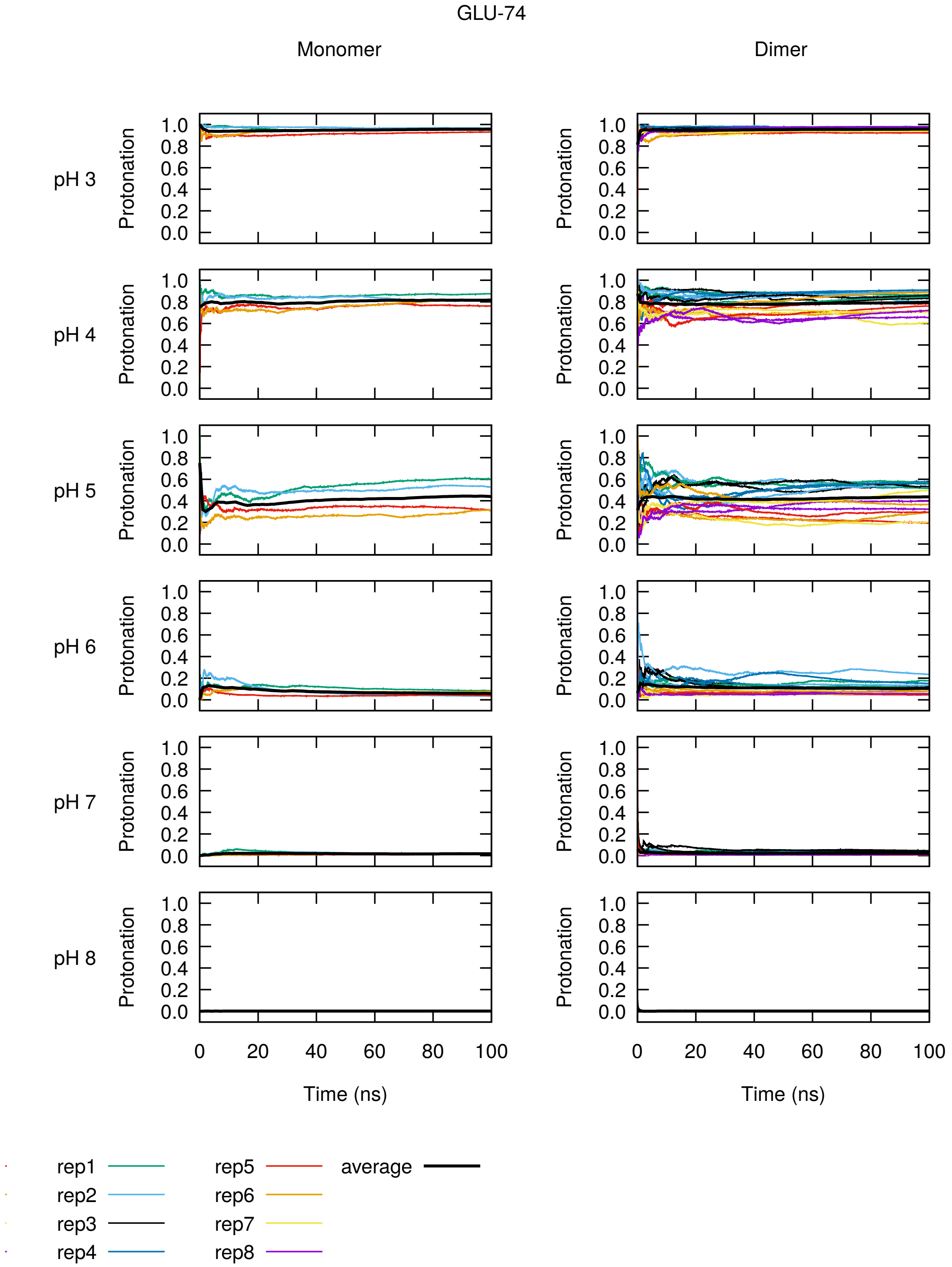

Figure S7: (continued, part 13)

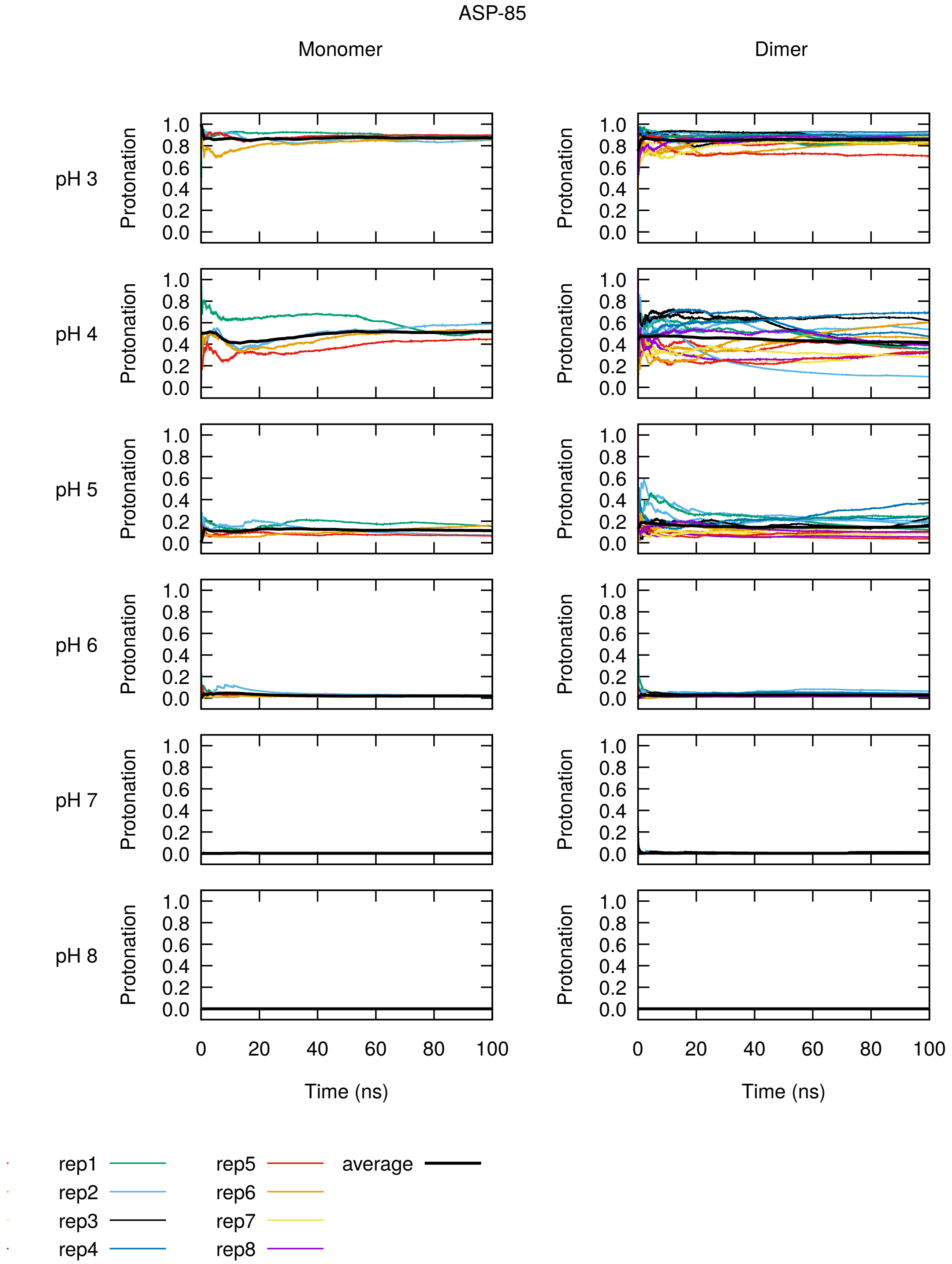

Figure S7: (continued, part 14)

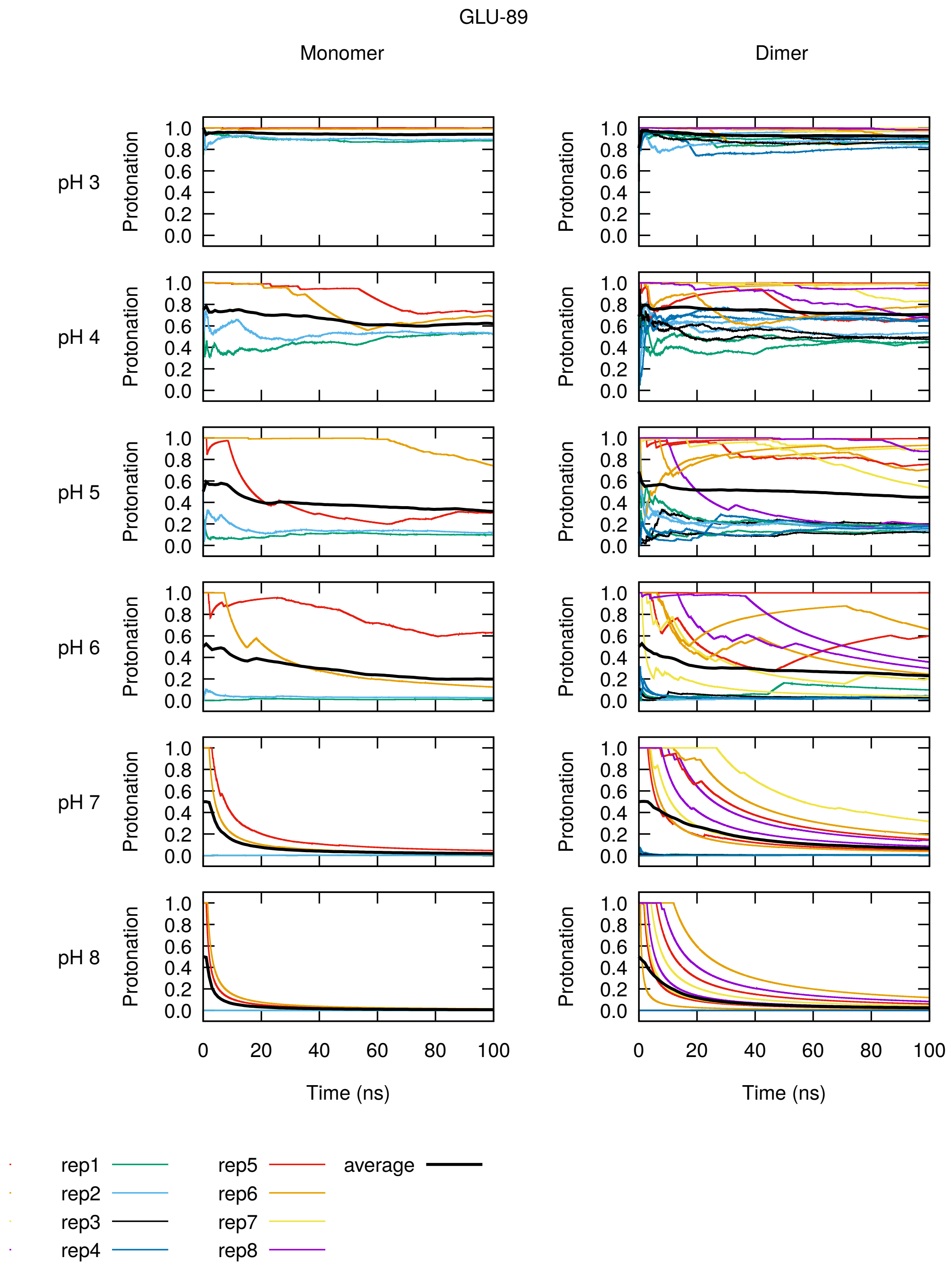

Figure S7: (continued, part 15)

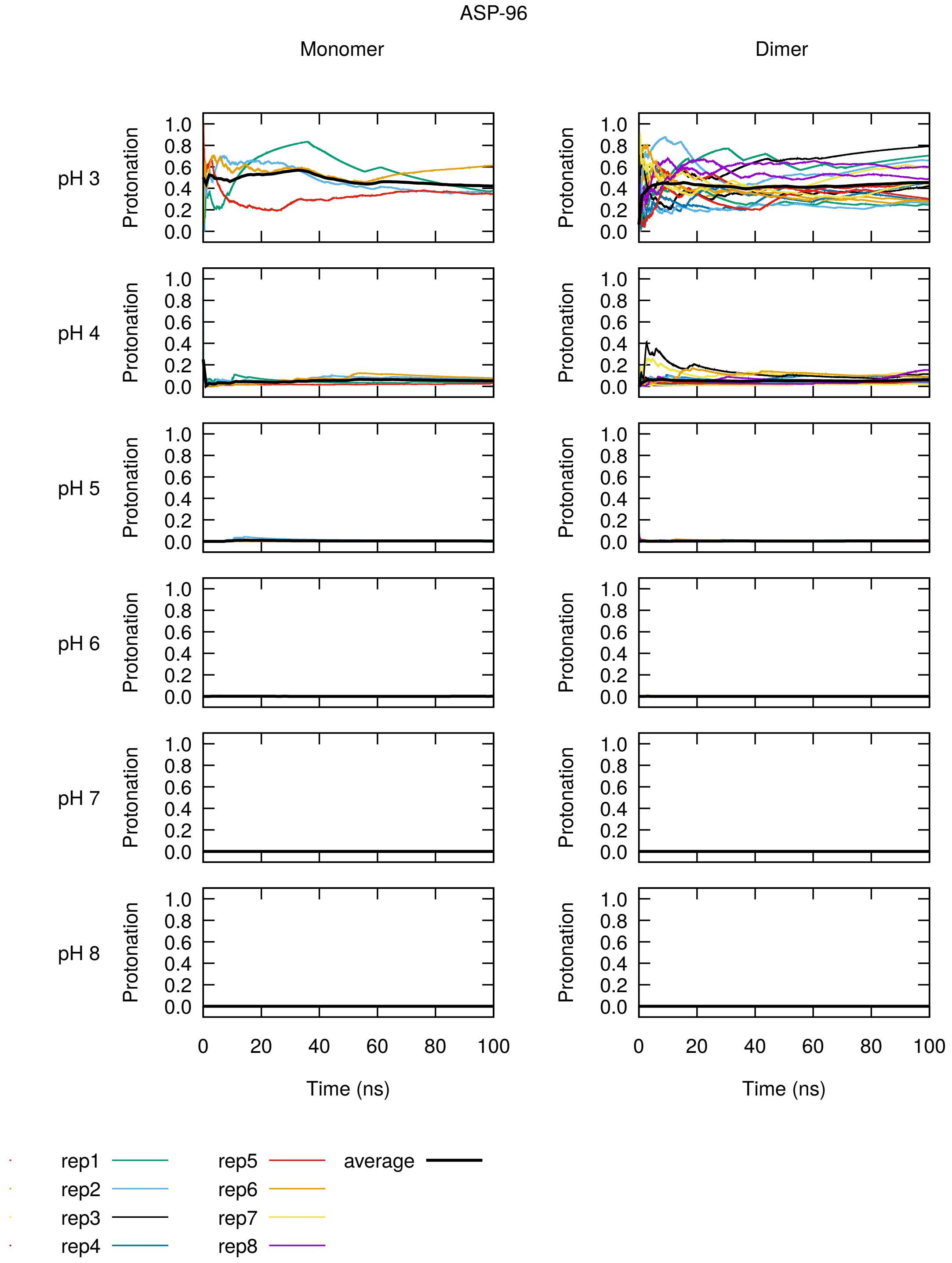

Figure S7: (continued, part 16)

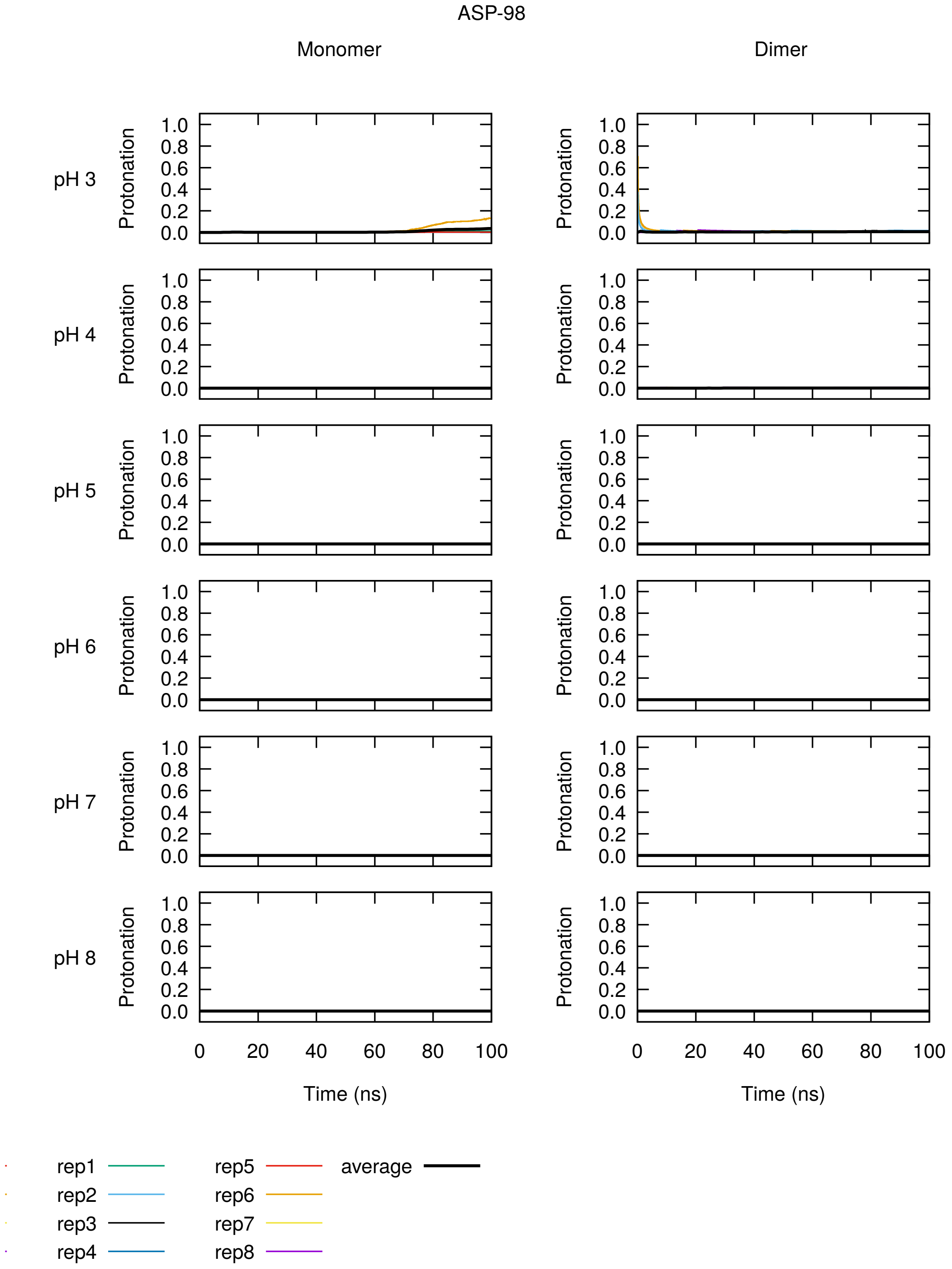

Figure S7: (continued, part 17)

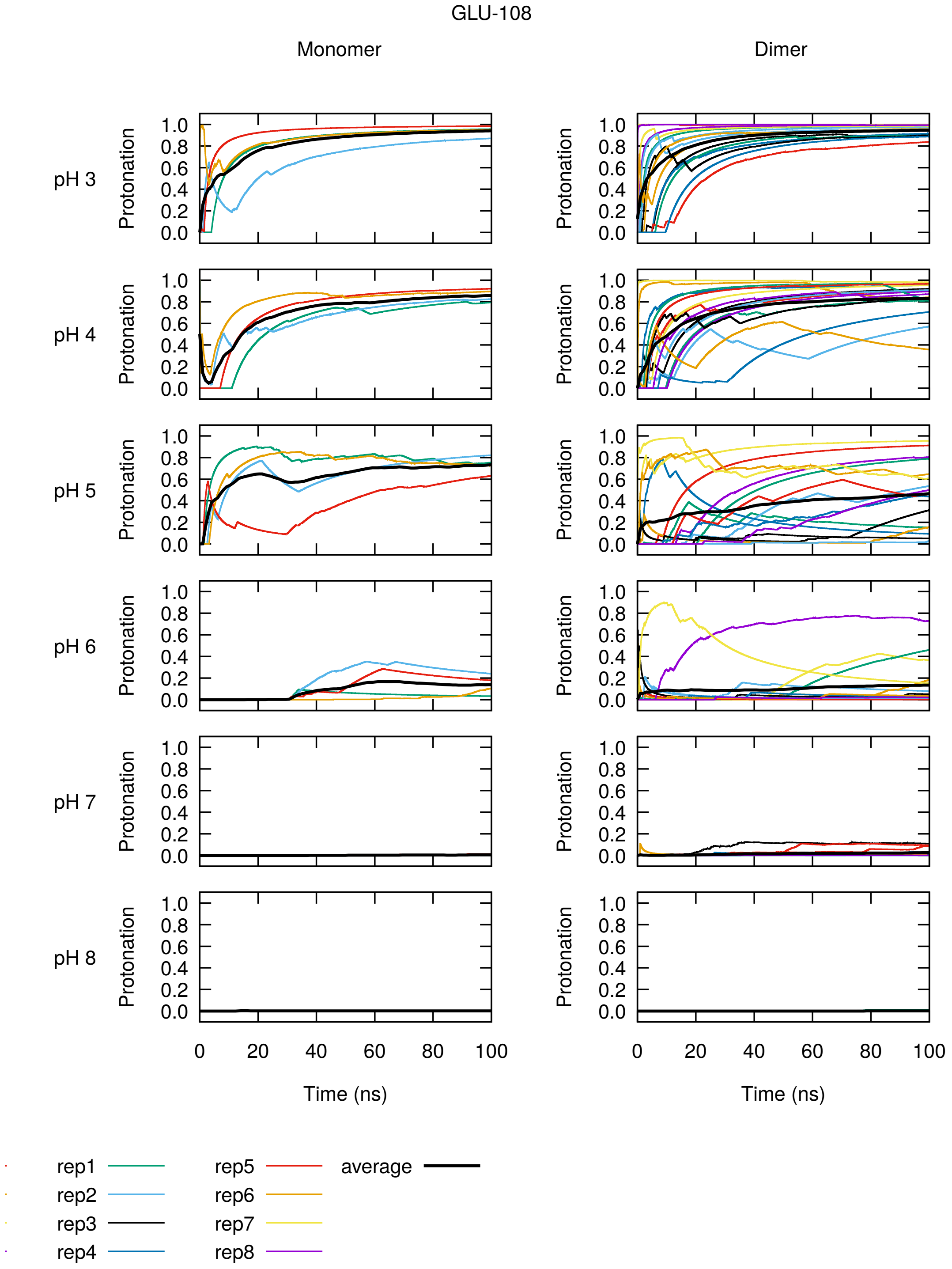

Figure S7: (continued, part 18)

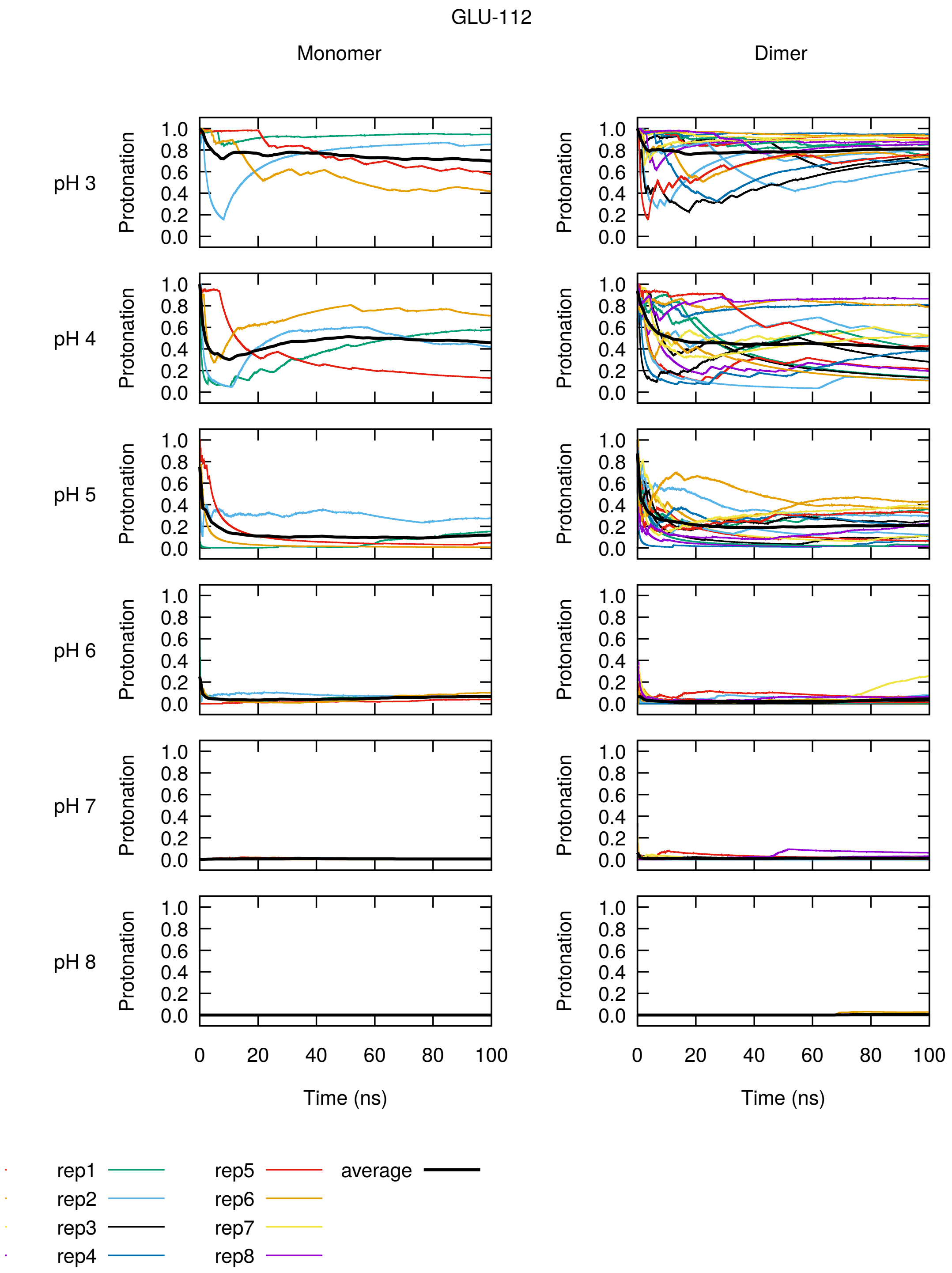

Figure S7: (continued, part 19)

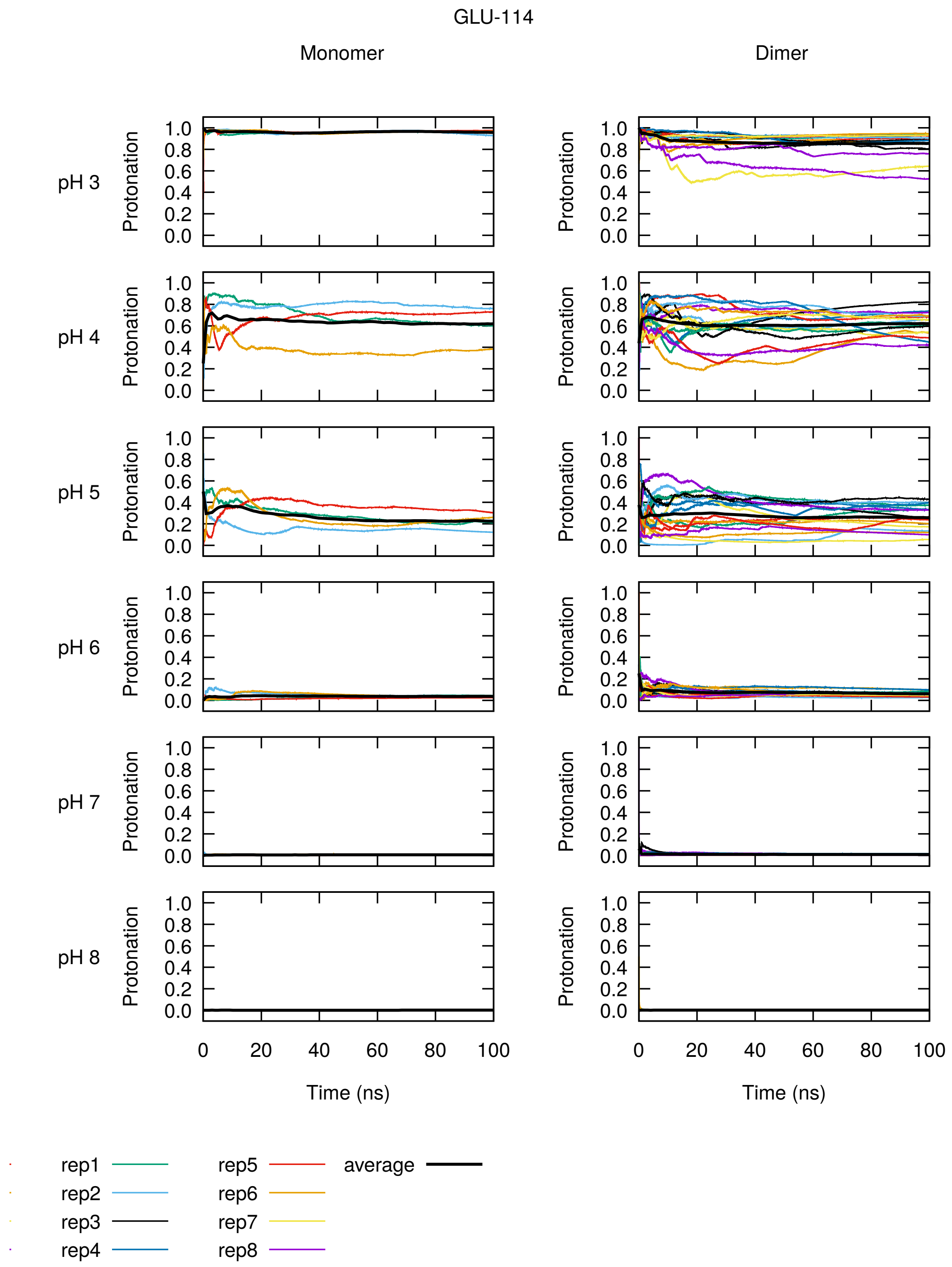

Figure S7: (continued, part 20)

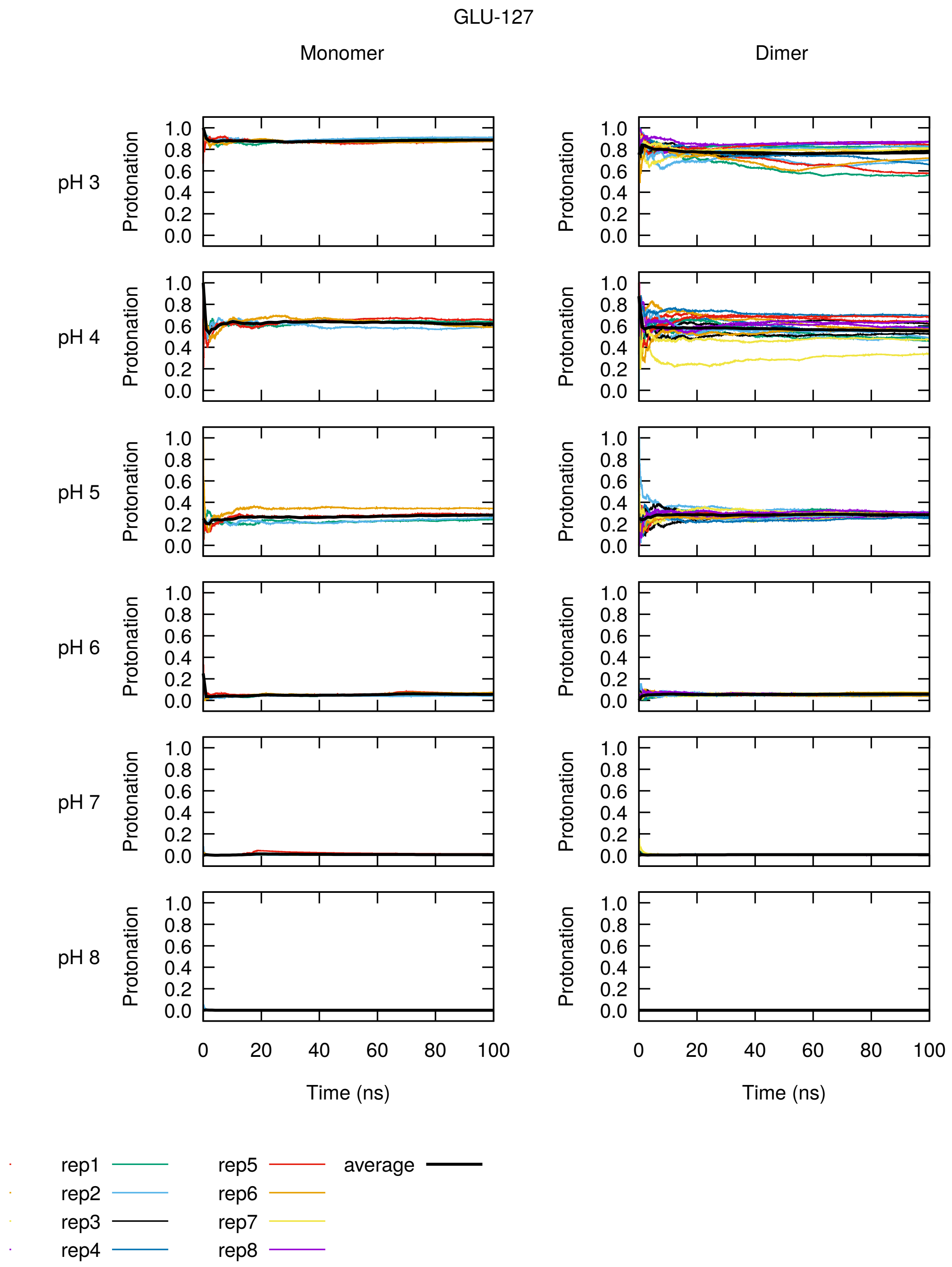

Figure S7: (continued, part 21)

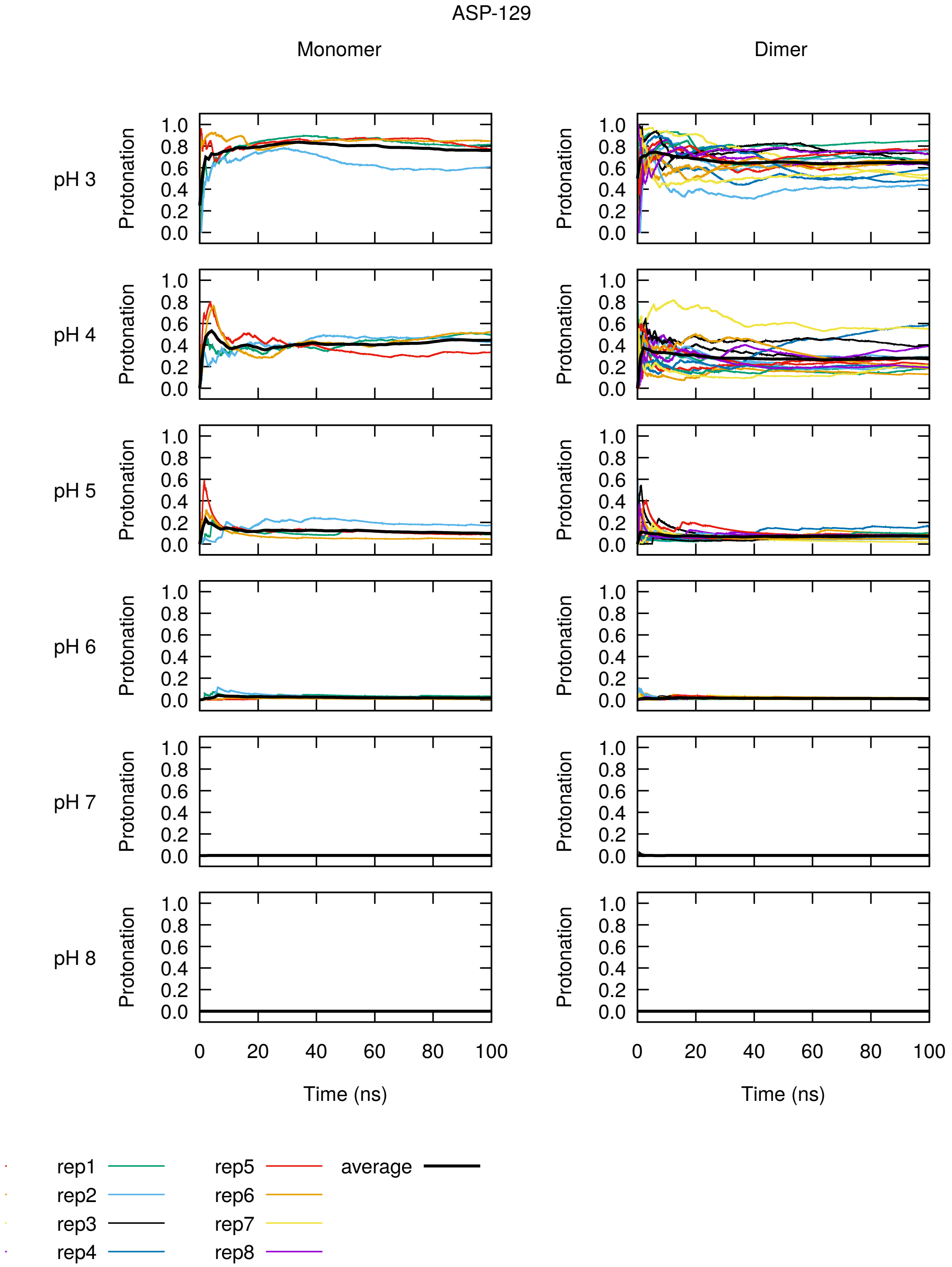

Figure S7: (continued, part 22)

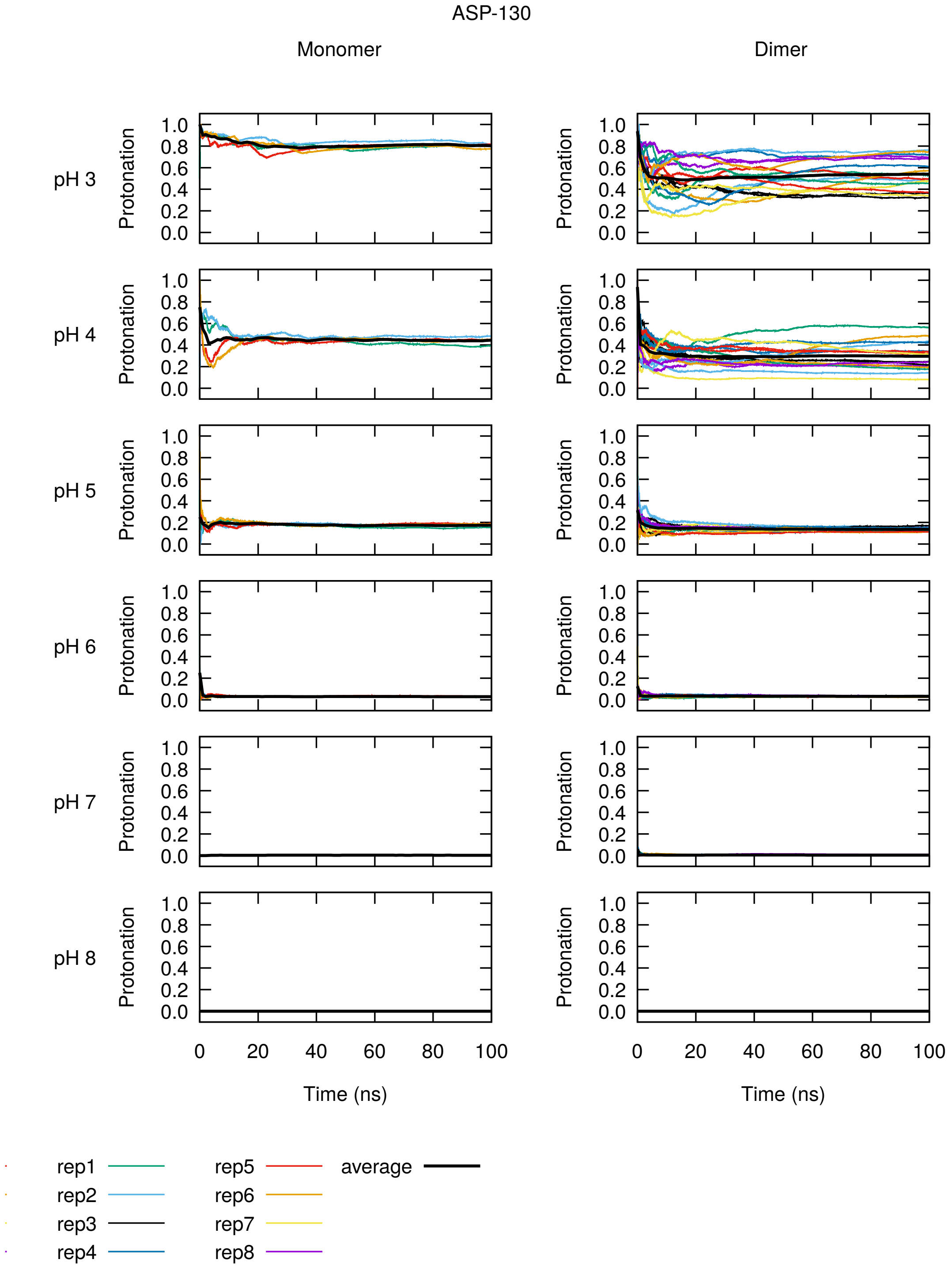

Figure S7: (continued, part 23)

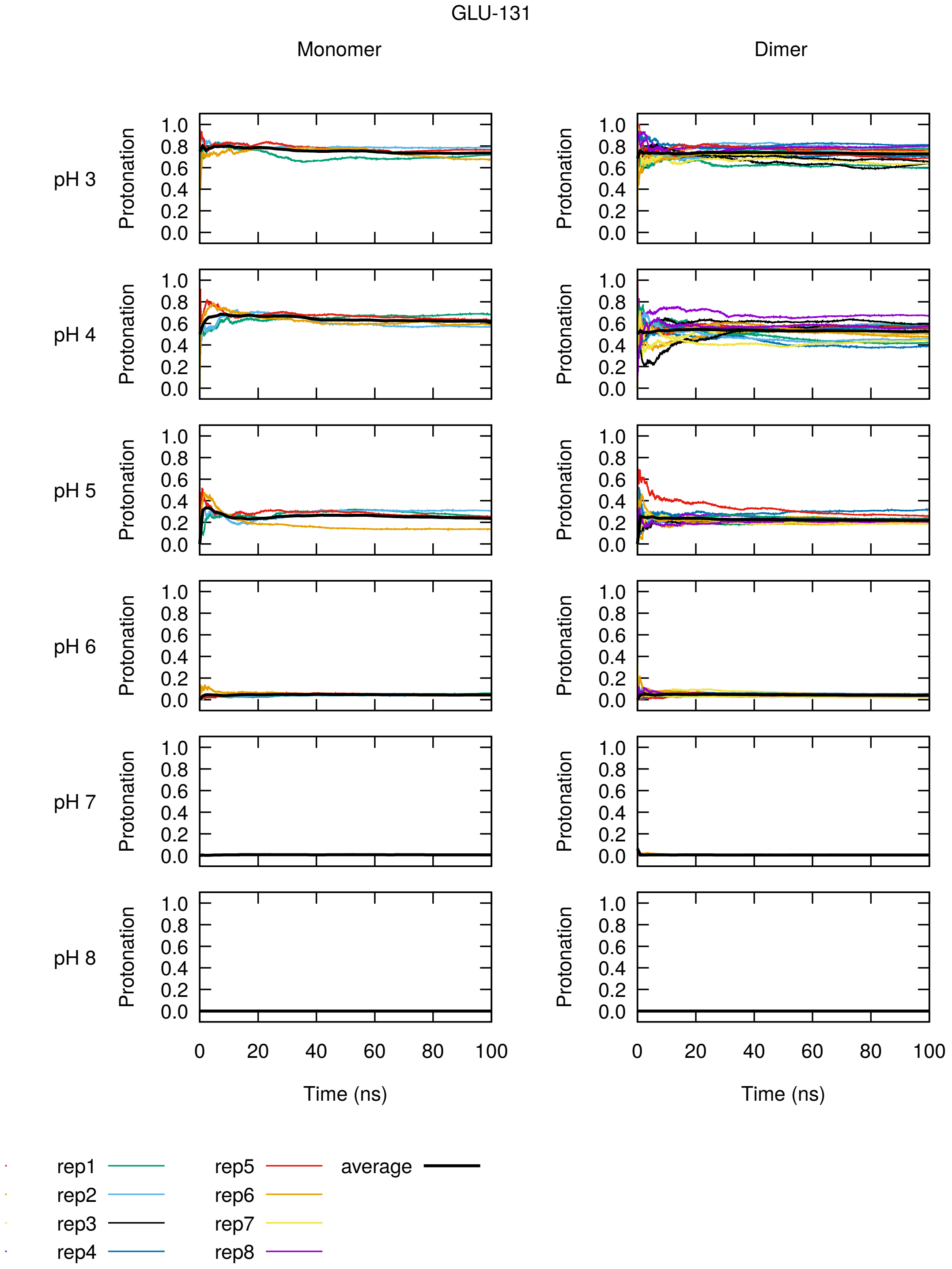

Figure S7: (continued, part 24)

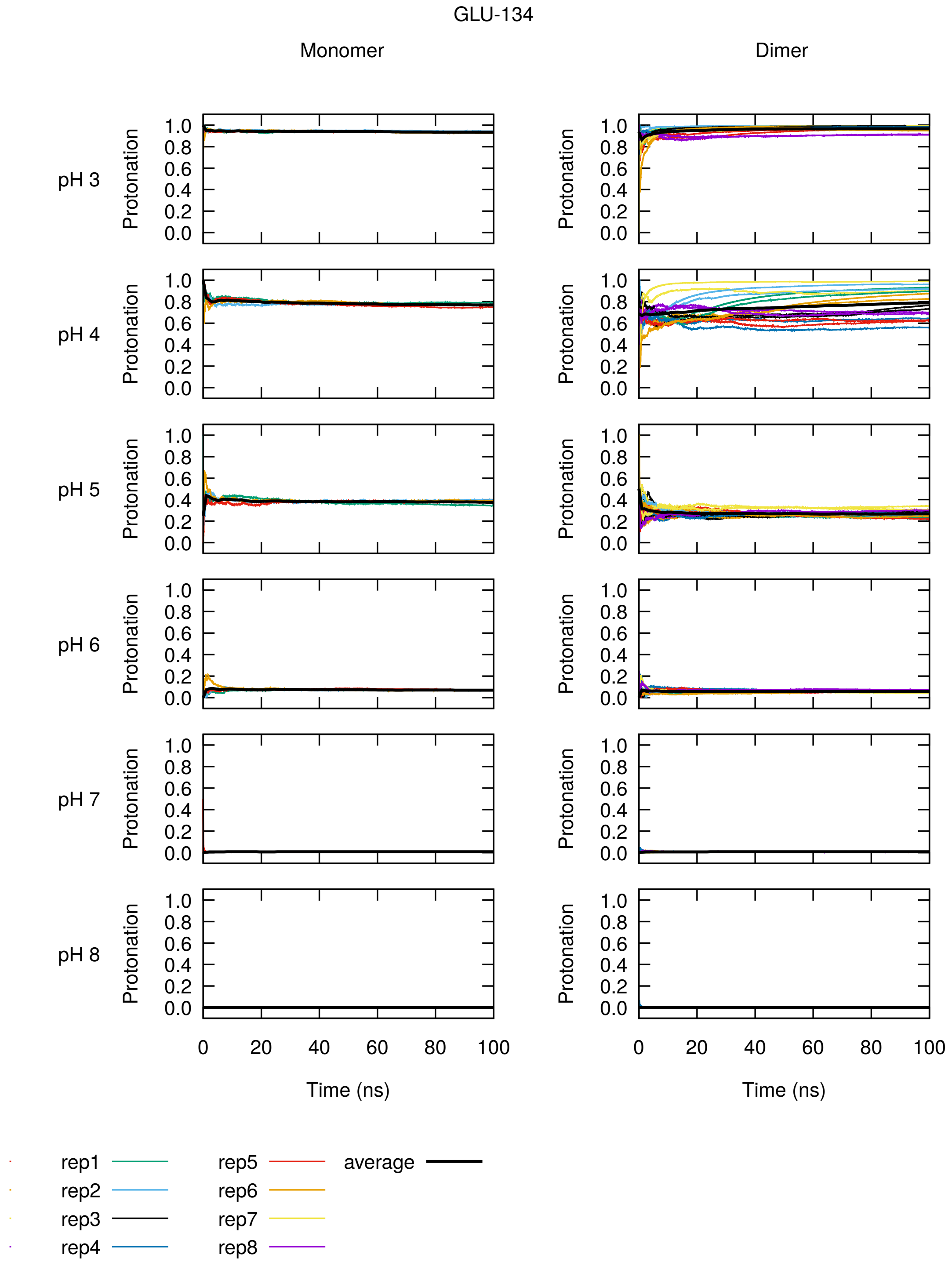

Figure S7: (continued, part 25)

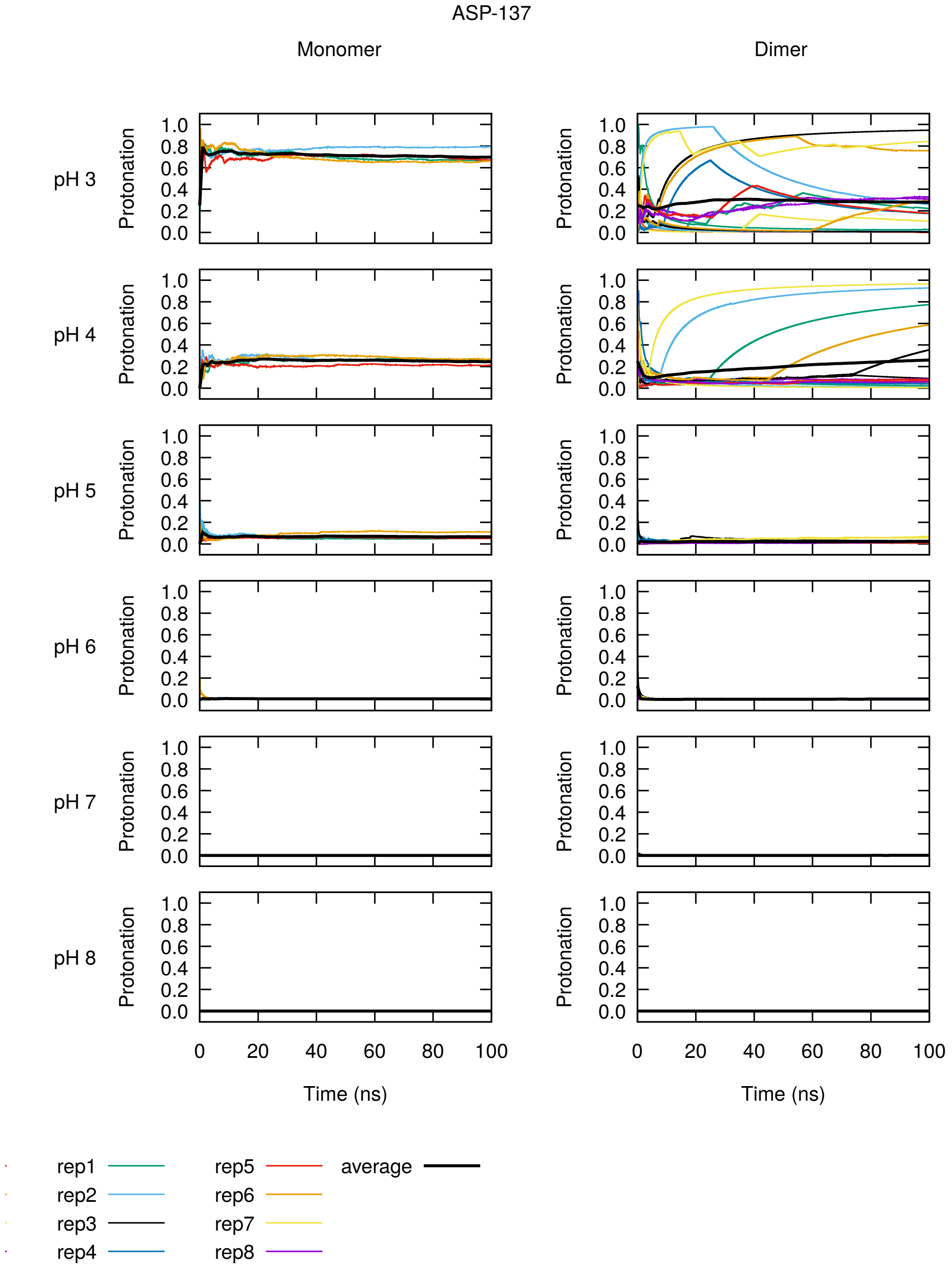

Figure S7: (continued, part 26)

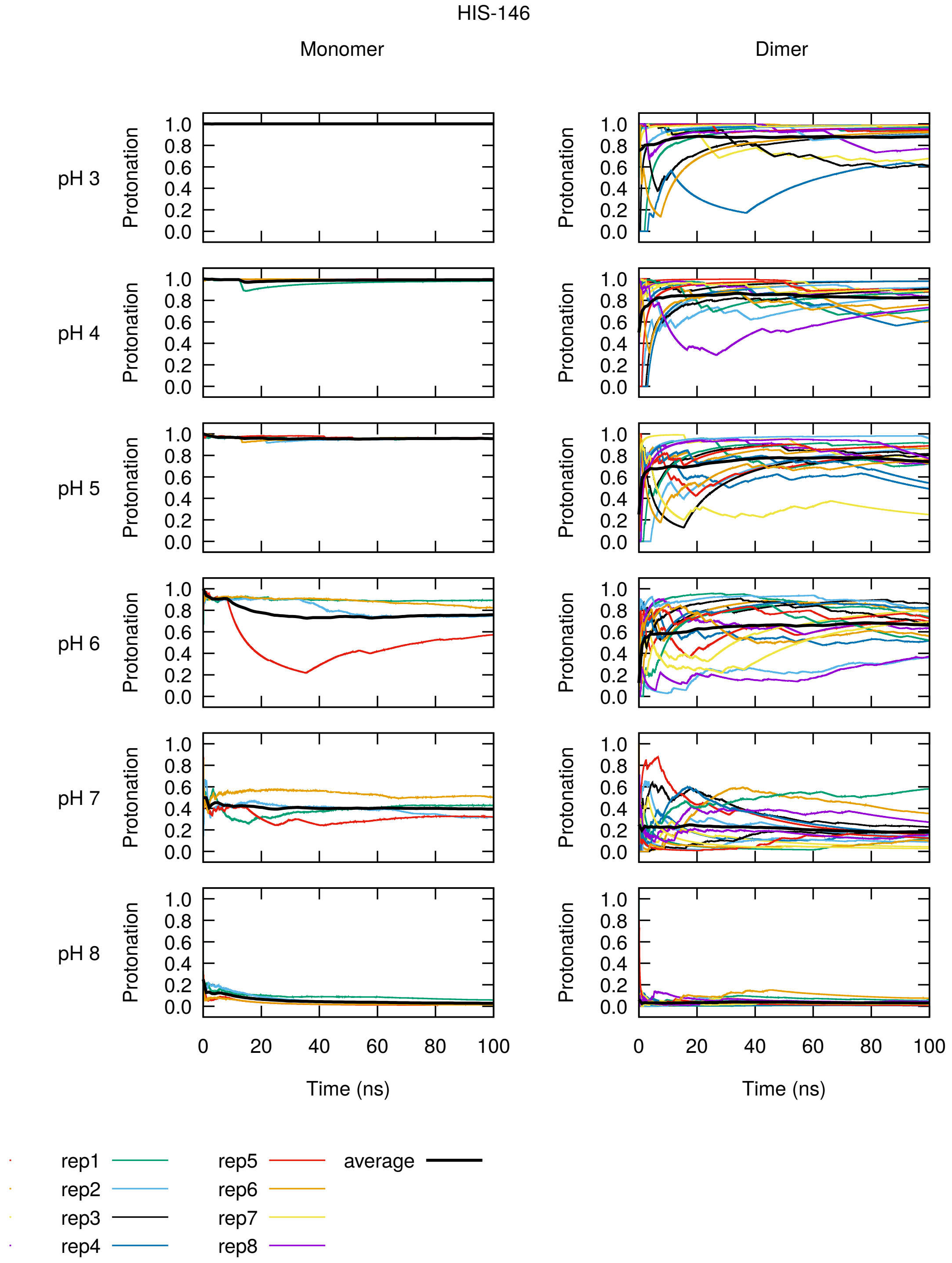

Figure S7: (continued, part 27)

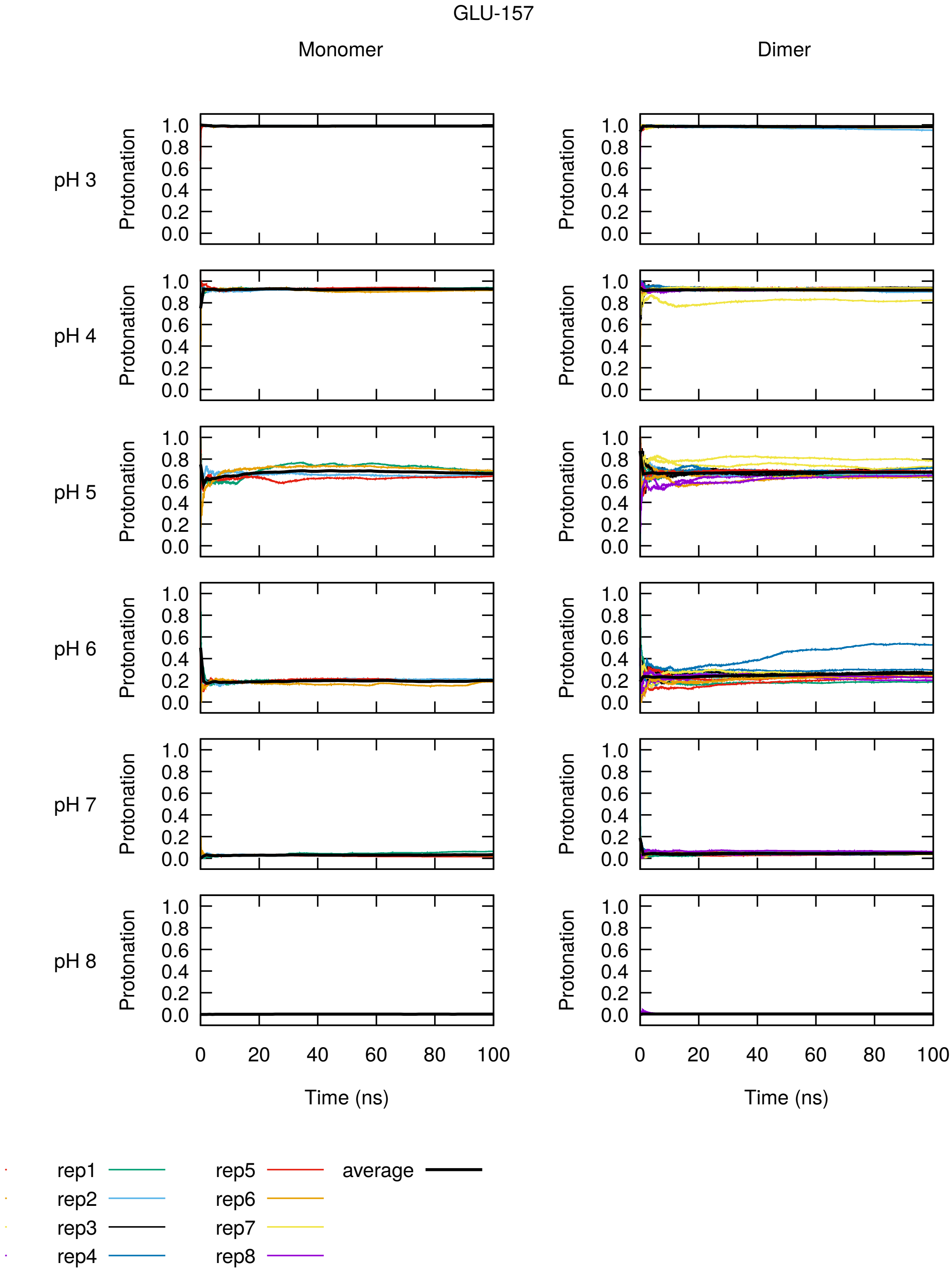

Figure S7: (continued, part 28)

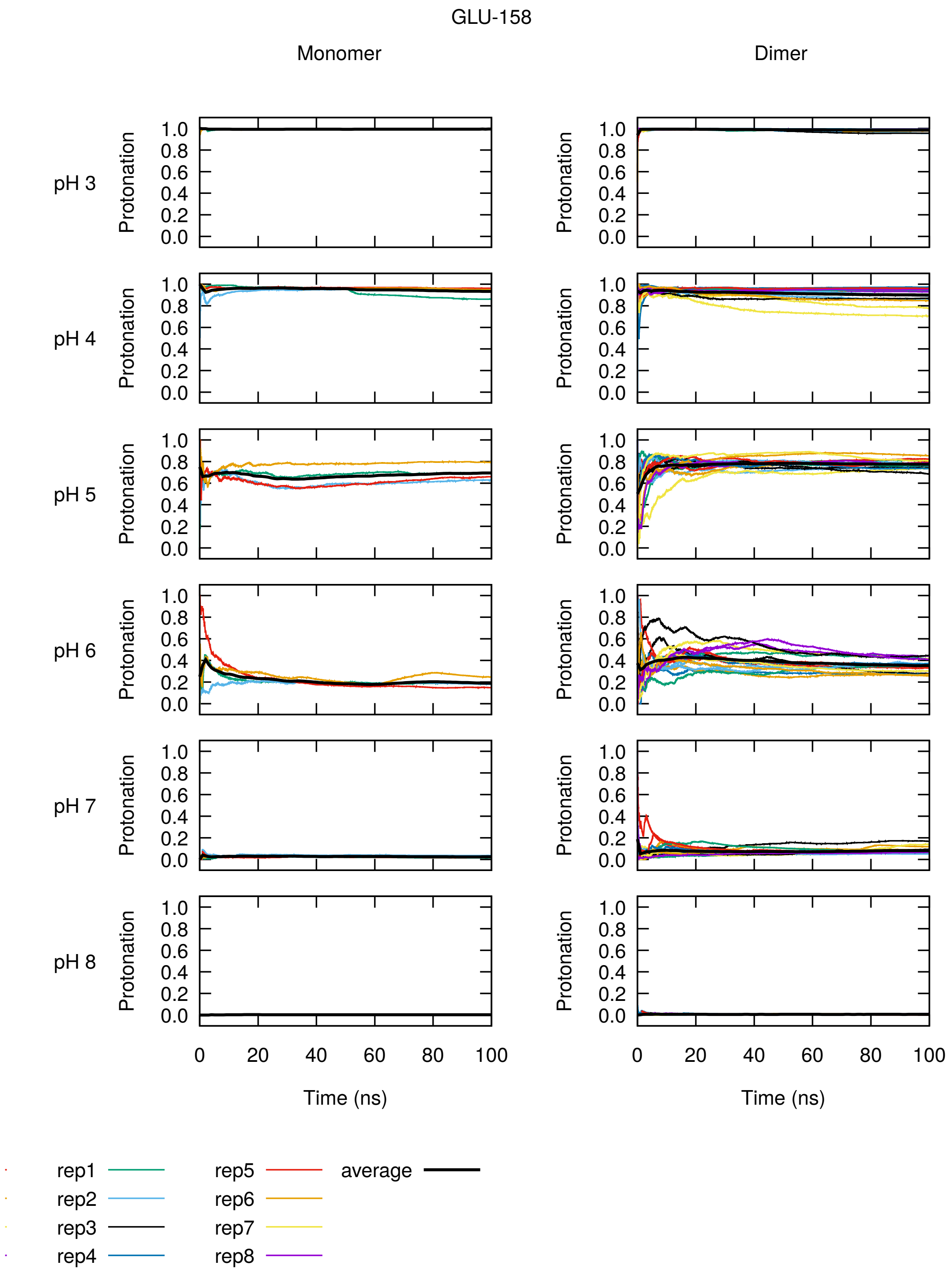

Figure S7: (continued, part 29)

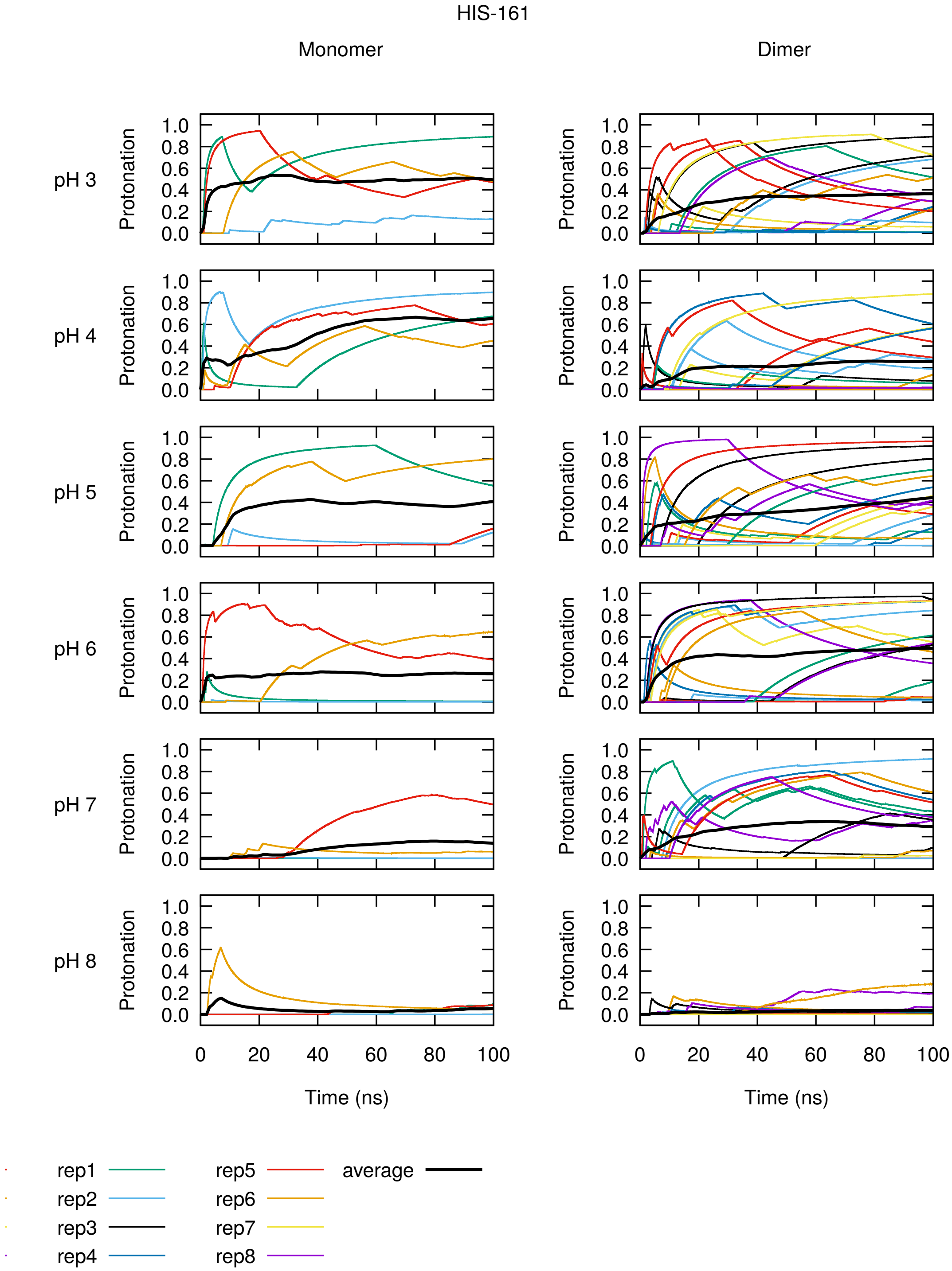

Figure S7: (continued, part 30)

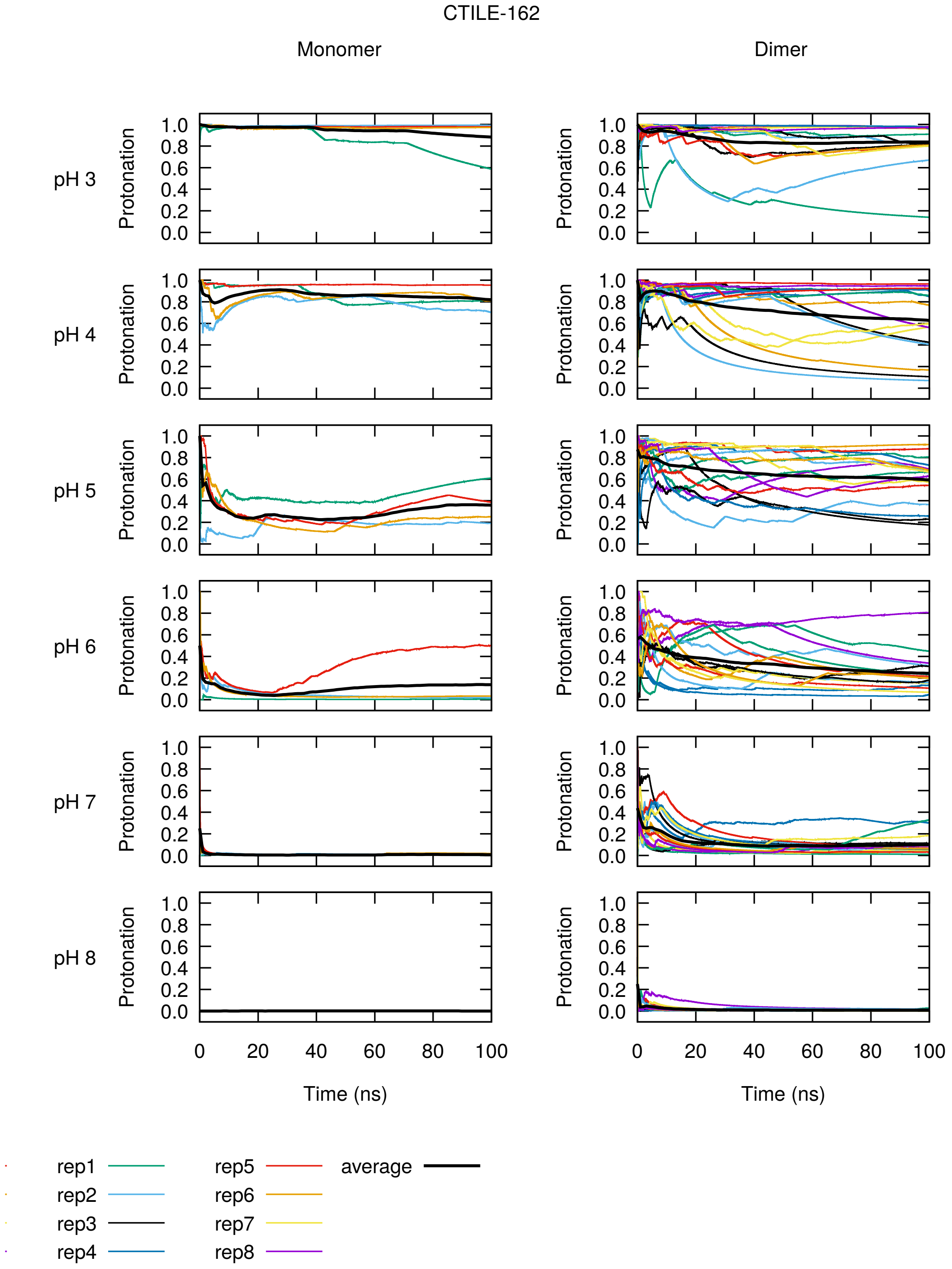

Figure S7: (continued, part 31)

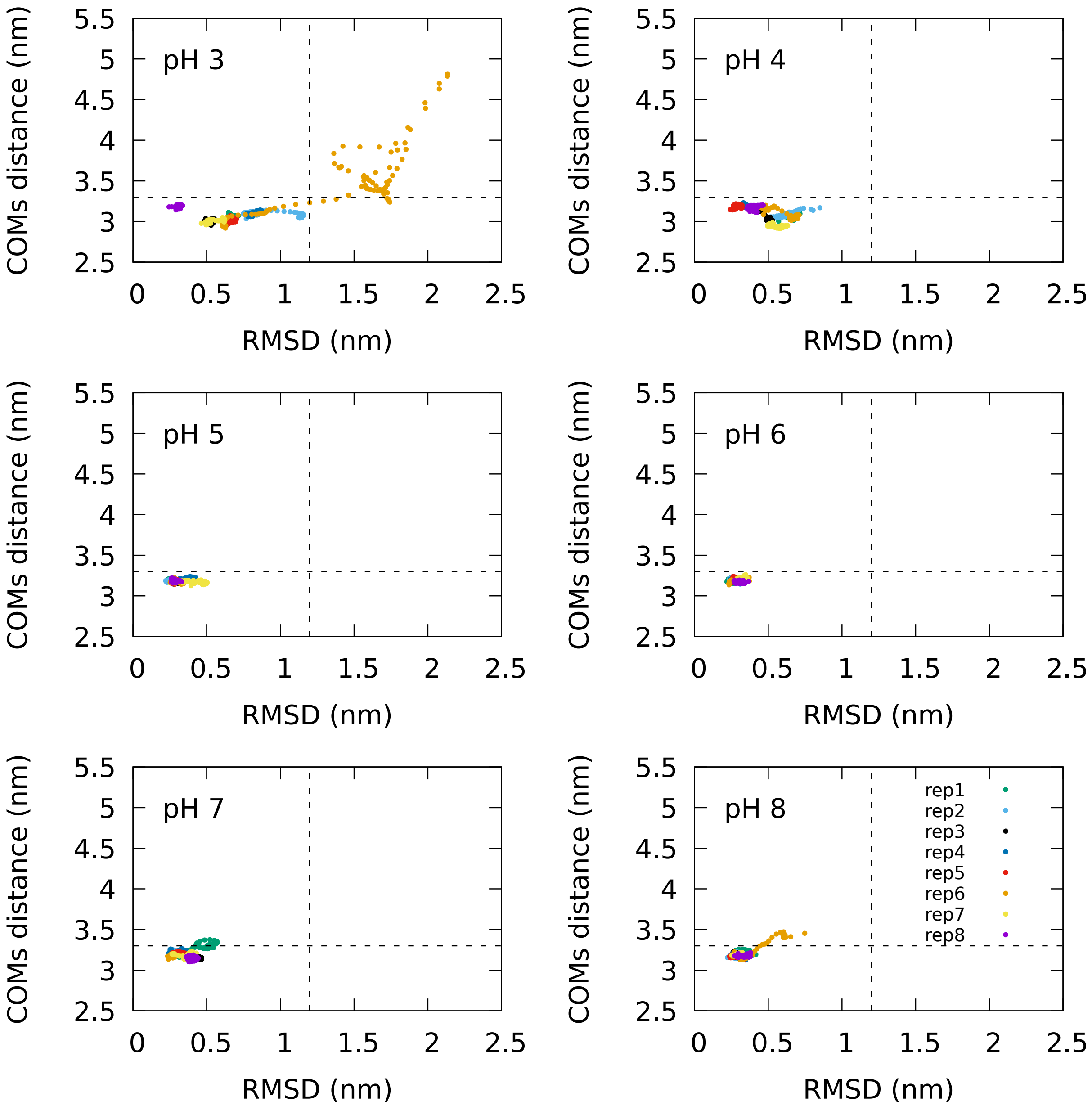

Figure S8: Identification of dissociated structures in the dimer simulations. The vertical axis corresponds to the distance between the centers of mass of the two dimer partners, and the horizontal axis to the RMSD of the dimer backbone relative to the initial dimer structure. The structures with a distance higher than 3.3 nm or an RMSD higher than 1.2 nm were considered dissociated and excluded from the analyses. Replicates 1–4 were started from an open conformation and 5–8 from a closed one.

3.2 Protonation Curves

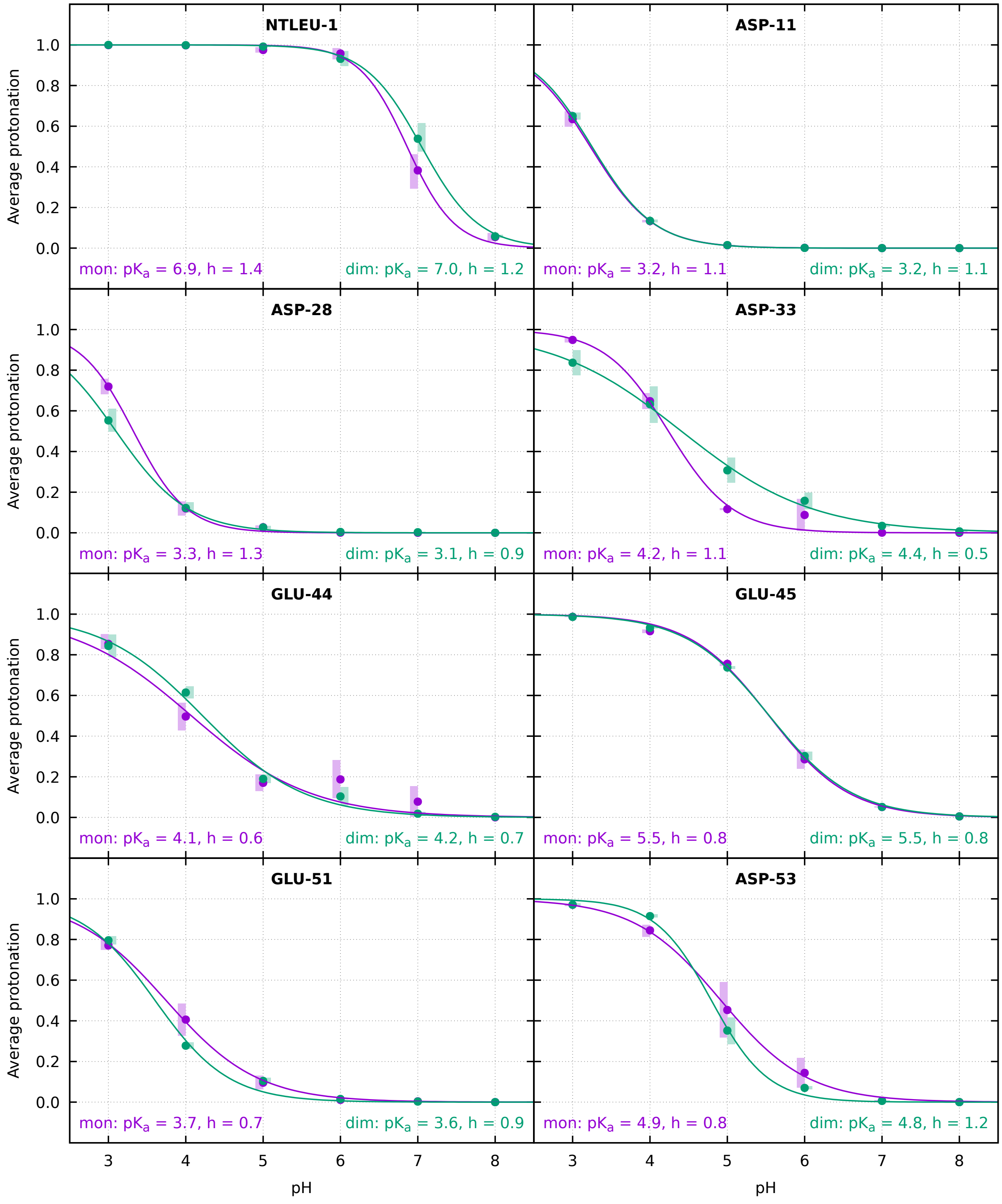

Figure S9: Average protonation of BLG sites in the monomer (purple) and dimer (green), showing the pK_a values and Hill coefficients h obtained from the fit of a Hill curve. Error bars are slightly displaced for easier visualization.

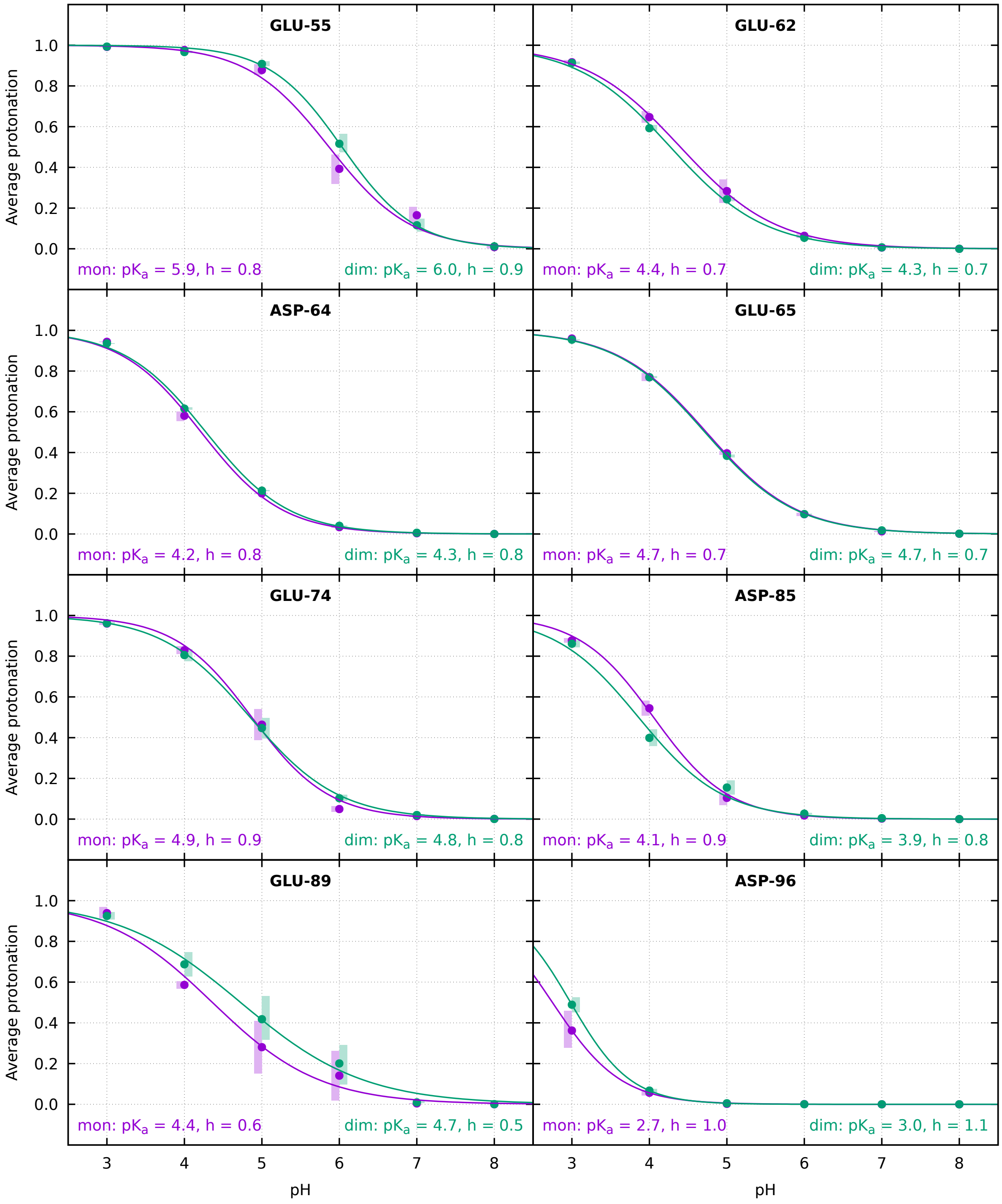

Figure S9: (continued, part 2)

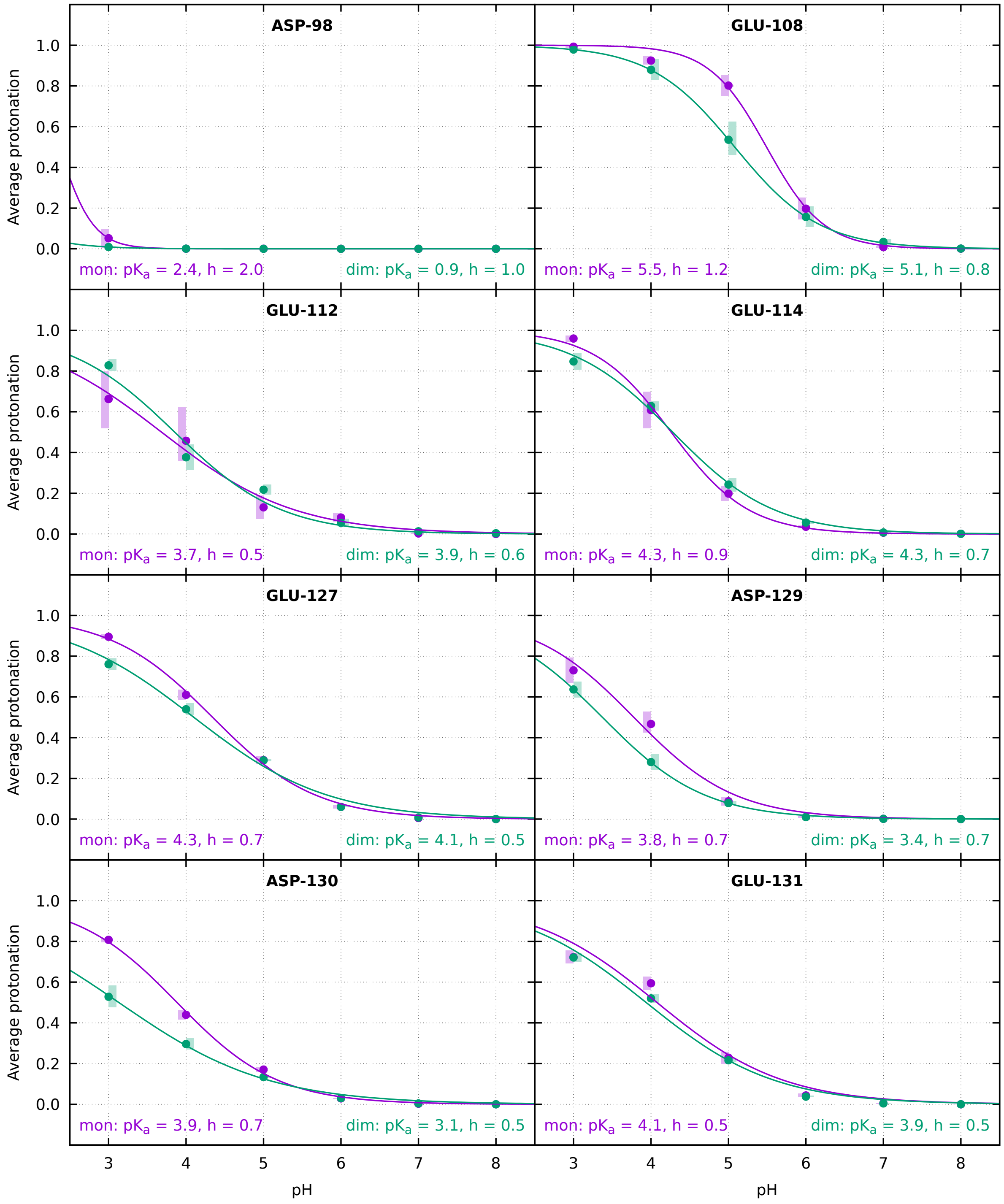

Figure S9: (continued, part 3)

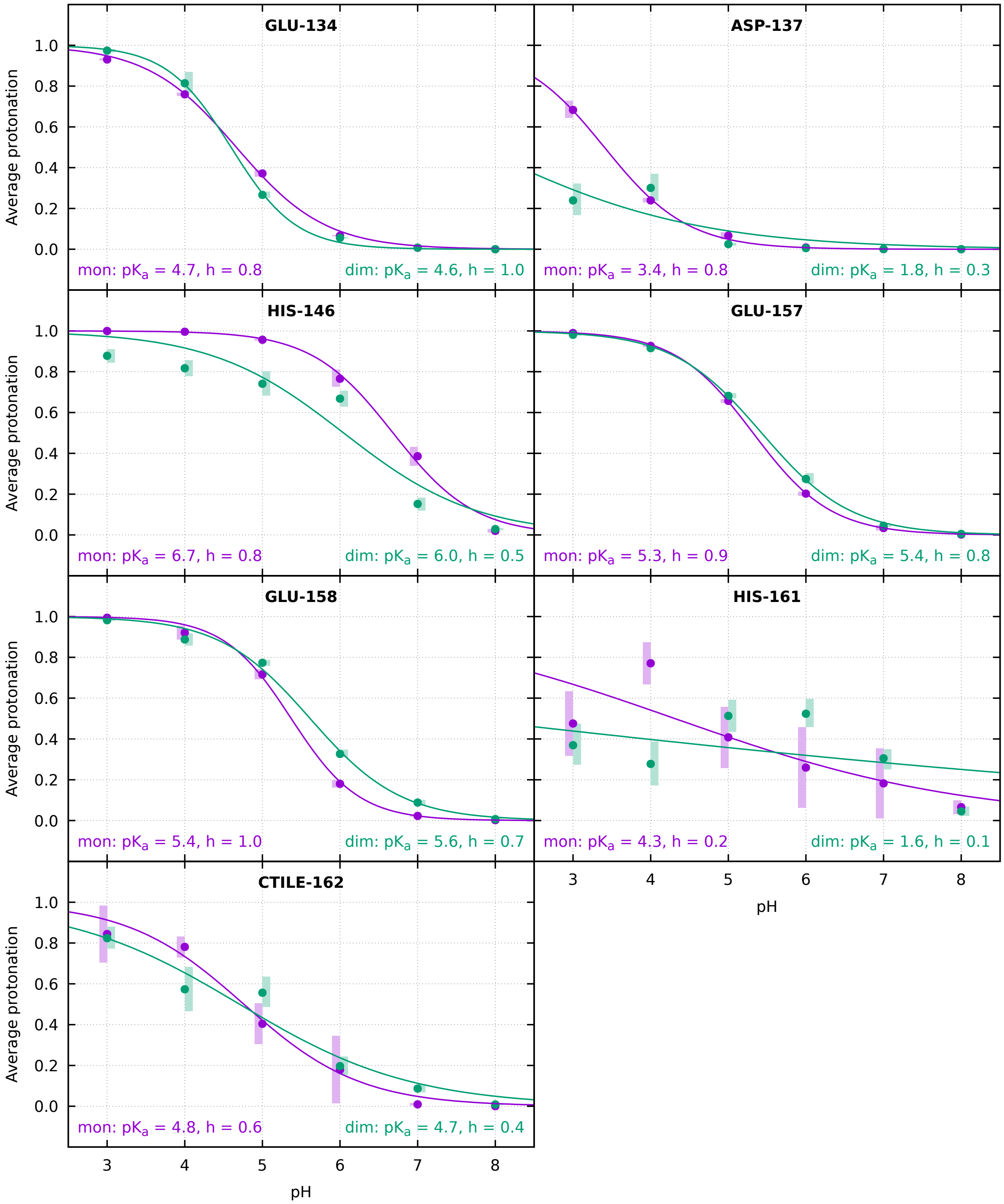

Figure S9: (continued, part 4)

3.3 Dimerization Free Energy

Table S3: Experimental BLG dimerization free energies used in Figure 4 of the main manuscript. The dimerization free energies were either taken directly from the original publication or computed from the reported association or dissociation constants (in which case the errors were estimated using the absolute differential formula[S9]). All free energies refer to a thermodynamic standard state of unit molarity.[S10] Mercadante et al. report that at pH 4.5 and 5.5 the dissociation constant has submicromolar values (< 1 μM) which correspond to $\Delta G < -8.19$ kcal/mol. In all cases the ionic strength was 0.1 M.

References	T (°C)	pH	ΔG (kcal/mol)
Mercadante et al. (2012)[S1]	25	2.5	-6.59 ± 0.29
	25	3.5	-7.36 ± 0.55
	25	4.5	< -8.19
	25	5.5	< -8.19
	25	6.5	-7.36 ± 0.43
	25	7.5	-6.78 ± 0.45
Bello et al. (2008)[S4]	25	7.0	-6.214 ± 0.004
Sakurai et al. (2001)[S3]	20	3.0	-5.640 ± 0.062
Tang and Adams (1973)[S11]	25	2.46	-4.51
Kelly and Reithel (1971)[S12]	20	5.2	-8.08 ± 0.11
McKenzie and Sawyer (1967)[S2]	20	7.5	-5.63 ± 0.13
Timasheff and Townend (1961)[S5]	25	2.7	-5.3 ± 0.1

3.4 Protonation Correlations

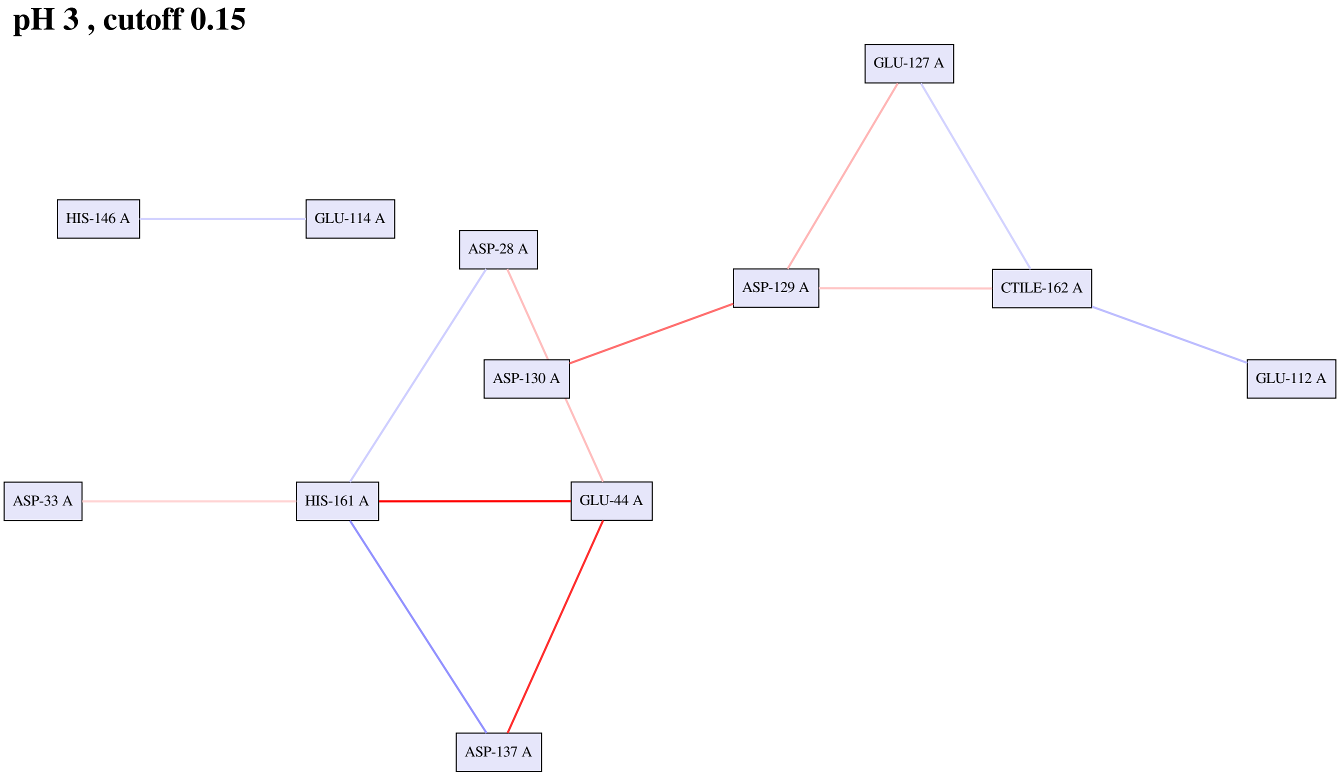

Figure S10: Networks of the protonation correlations in BLG **monomer** that are above 0.15 in absolute value. At pH 8, no correlations above this cutoff are observed. Red/blue indicate negative/positive correlations and more intense colors correspond to higher absolute values. The strongest negative correlation is −0.58 (at pH 3) and the strongest positive correlation is 0.35 (at pH 6). Networks were drawn with the Graphviz software package (https://graphviz.org).

pH 4 , cutoff 0.15

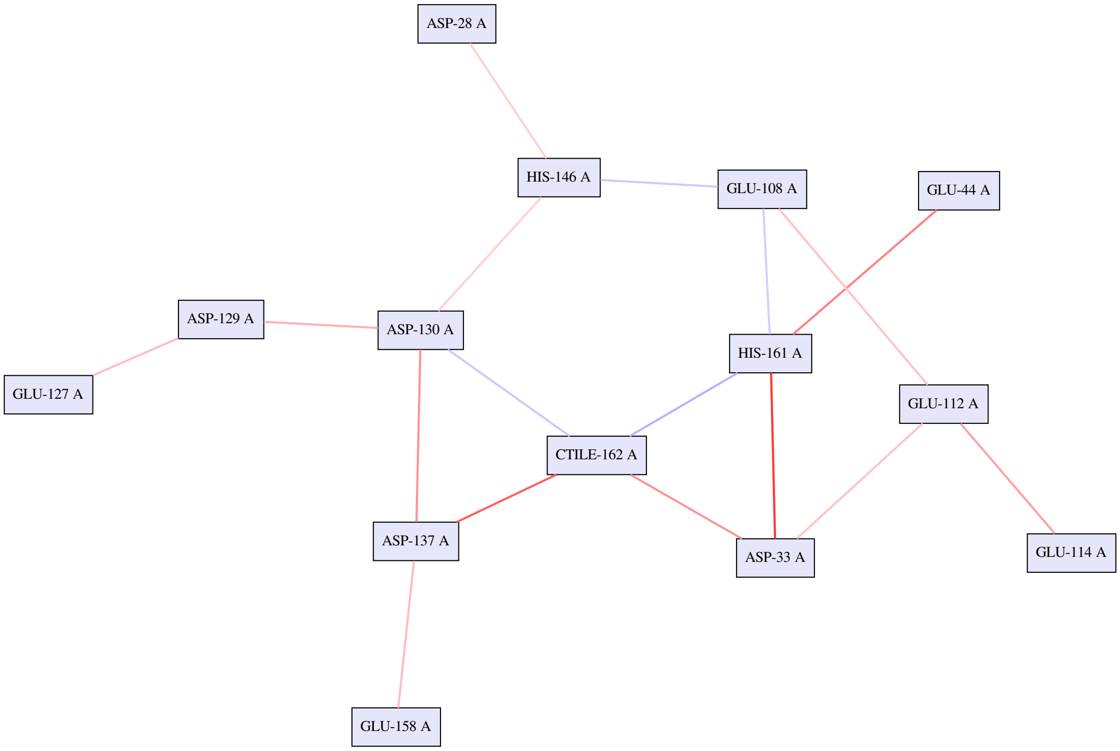

Figure S10: (continued, part 2)

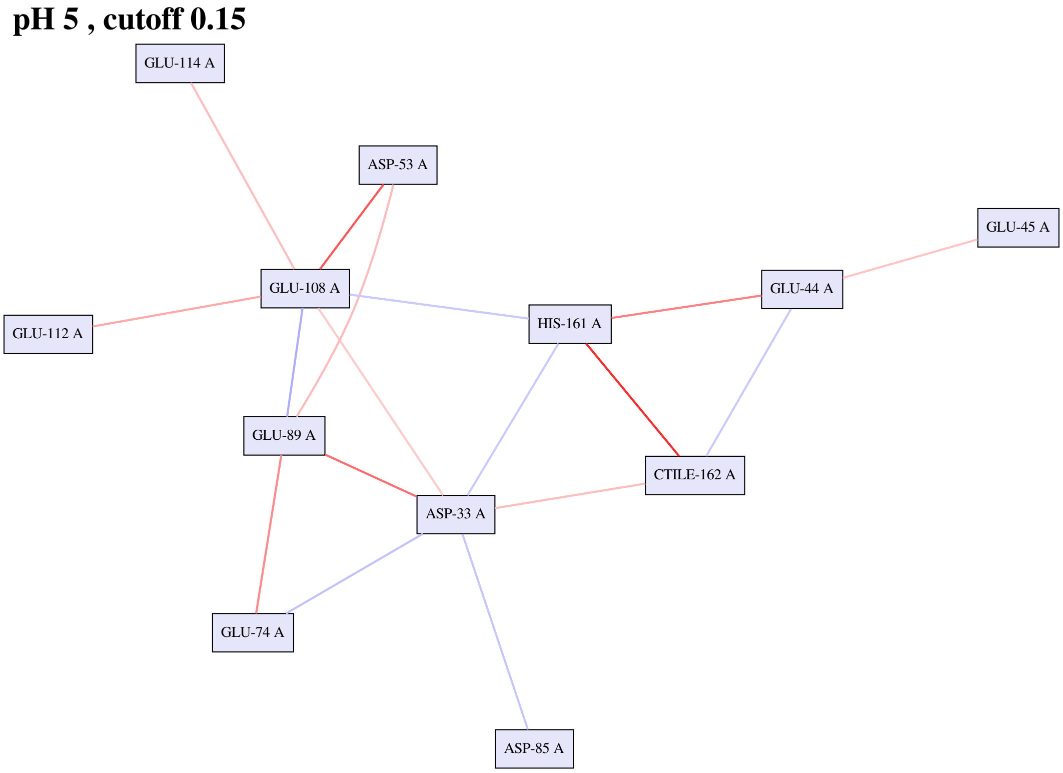

Figure S10: (continued, part 3)

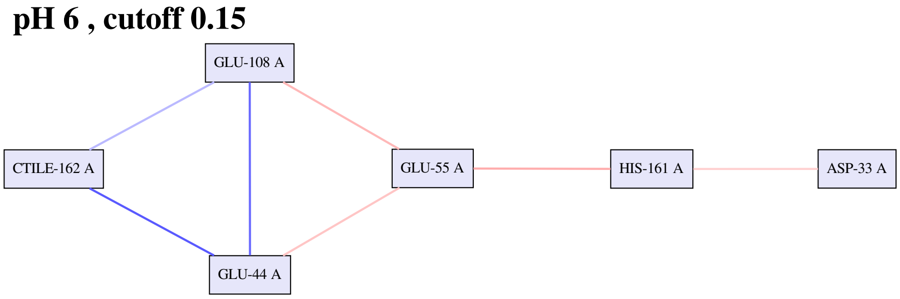

Figure S10: (continued, part 4)

pH 3 , cutoff 0.15

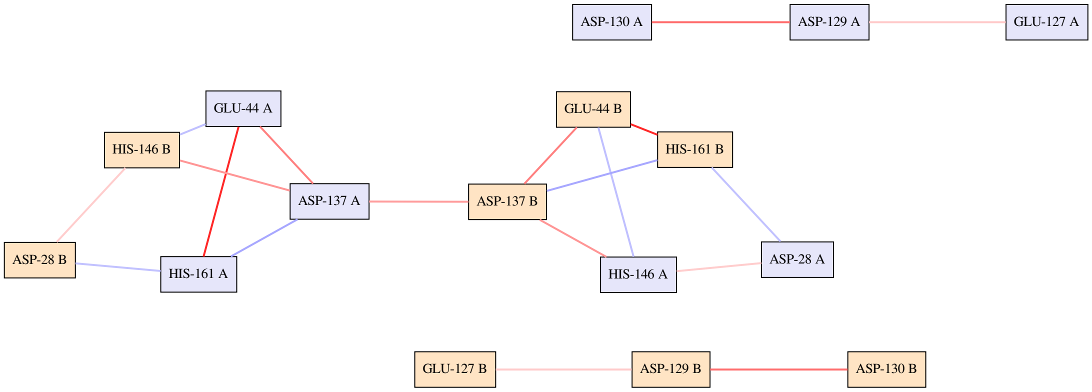

Figure S11: Networks of the protonation correlations in BLG **dimer** that are above 0.15 in absolute value. At pH 8, no correlations above this cutoff are observed. Red/blue indicate negative/positive correlations and more intense colors correspond to higher absolute values. The strongest negative correlation is −0.43 (at pH 3) and the strongest positive correlation is 0.45 (at pH 6). Different rectangle colors represent different dimer partners. Networks were drawn with the Graphviz software package (https://graphviz.org).

pH 4 , cutoff 0.15

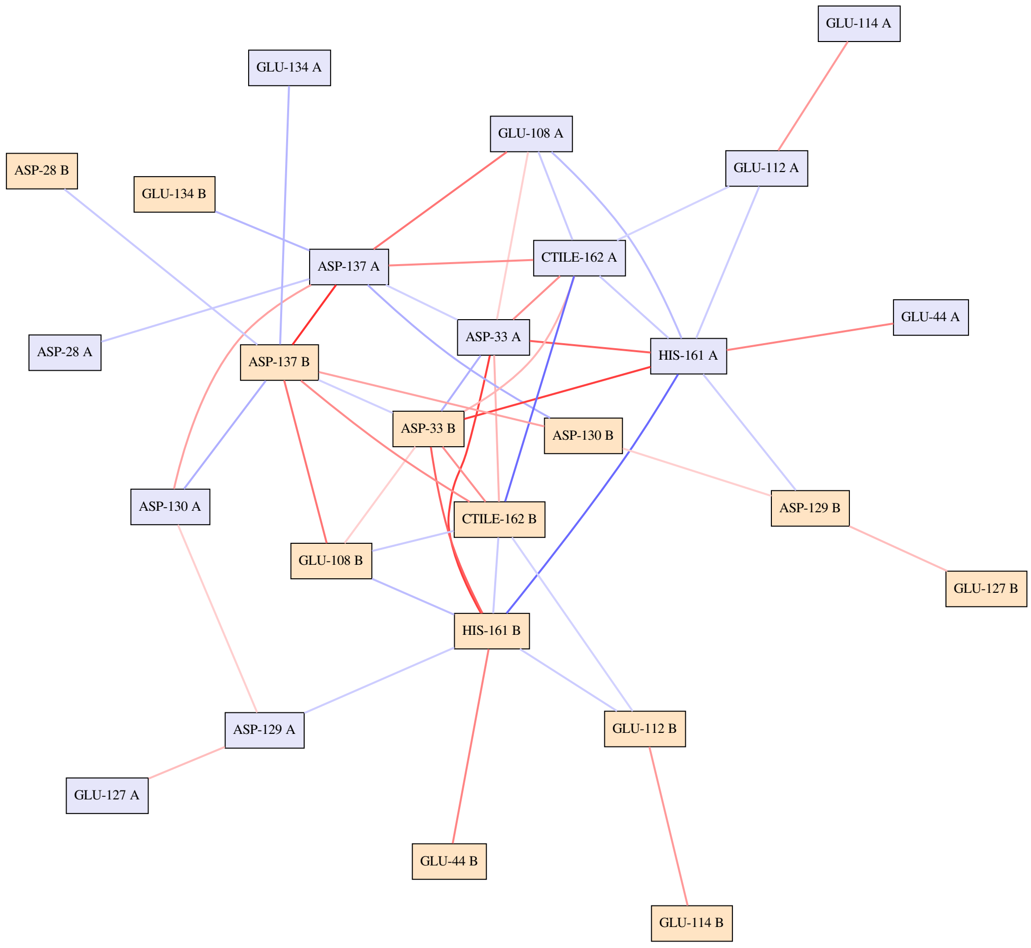

Figure S11: (continued, part 2)

pH 5 , cutoff 0.15

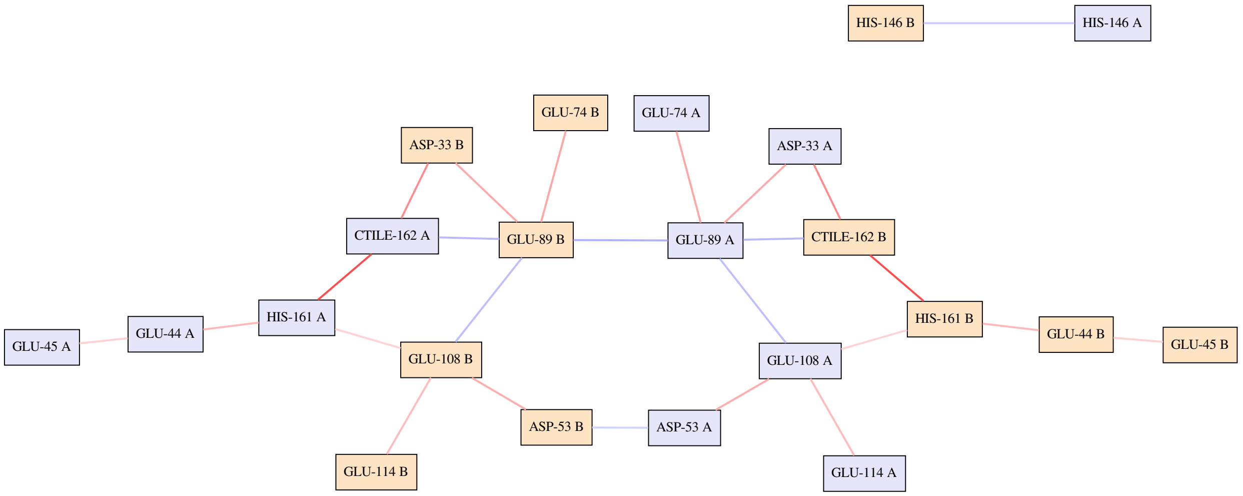

Figure S11: (continued, part 3)

pH 6 , cutoff 0.15

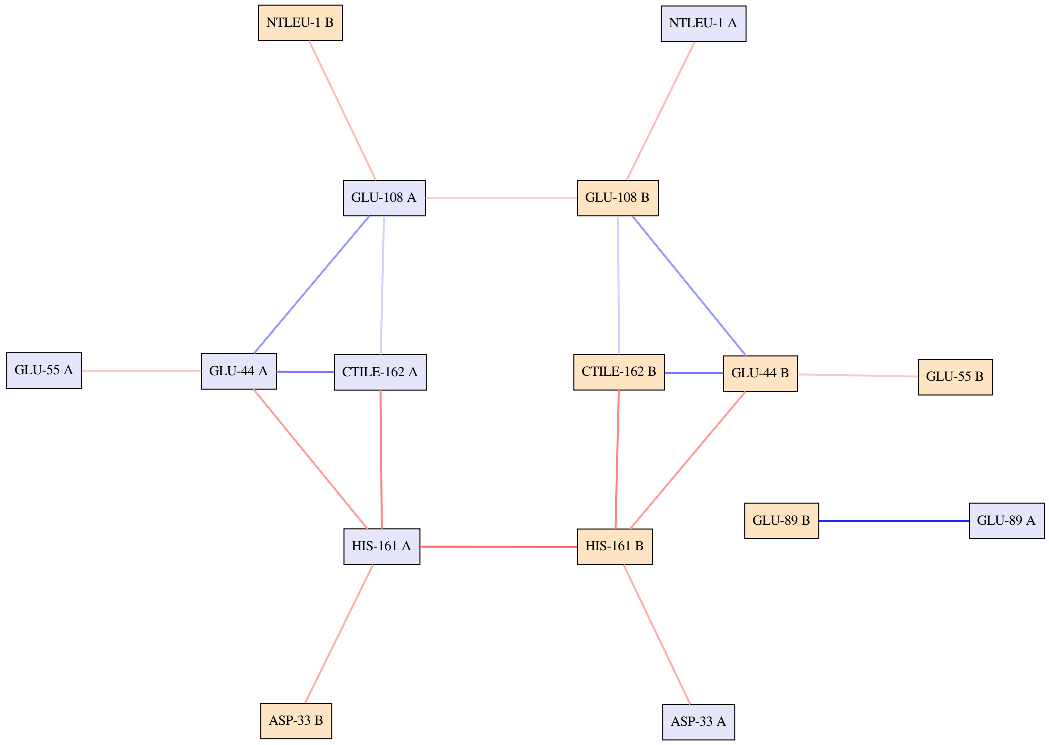

Figure S11: (continued, part 4)

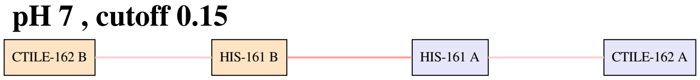

Figure S11: (continued, part 5)

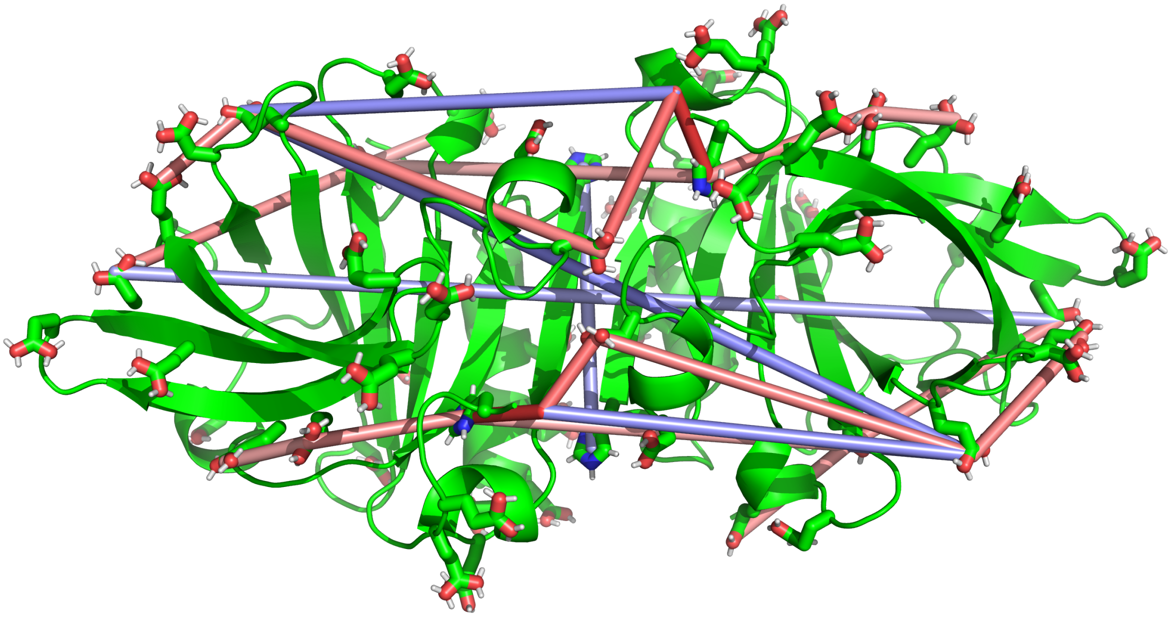

Figure S12: BLG structure showing the network of the protonation correlations in the dimer, at pH 5, that are above 0.15 in absolute value. Red/blue indicate negative/positive correlations and more intense colors correspond to higher absolute values.

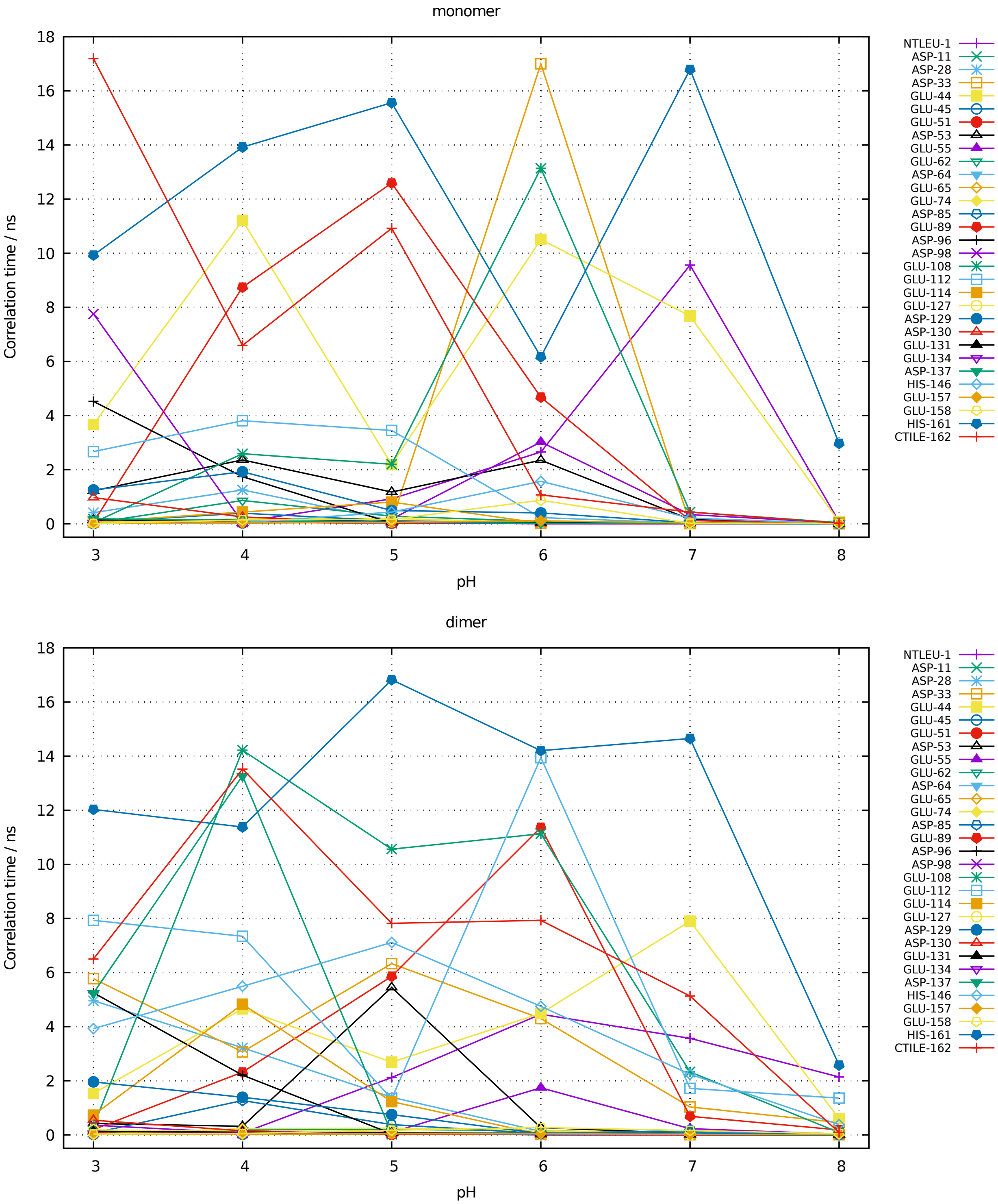

Figure S13: Correlation time of the proton occupancy (0 or 1) of the BLG sites in the monomer (**top**) and dimer (**bottom**), as a function of pH. This was estimated as the time at which the protonation autocorrelation function becomes lower than 0.1.[S13]

3.5 Electrostatic Complementarity

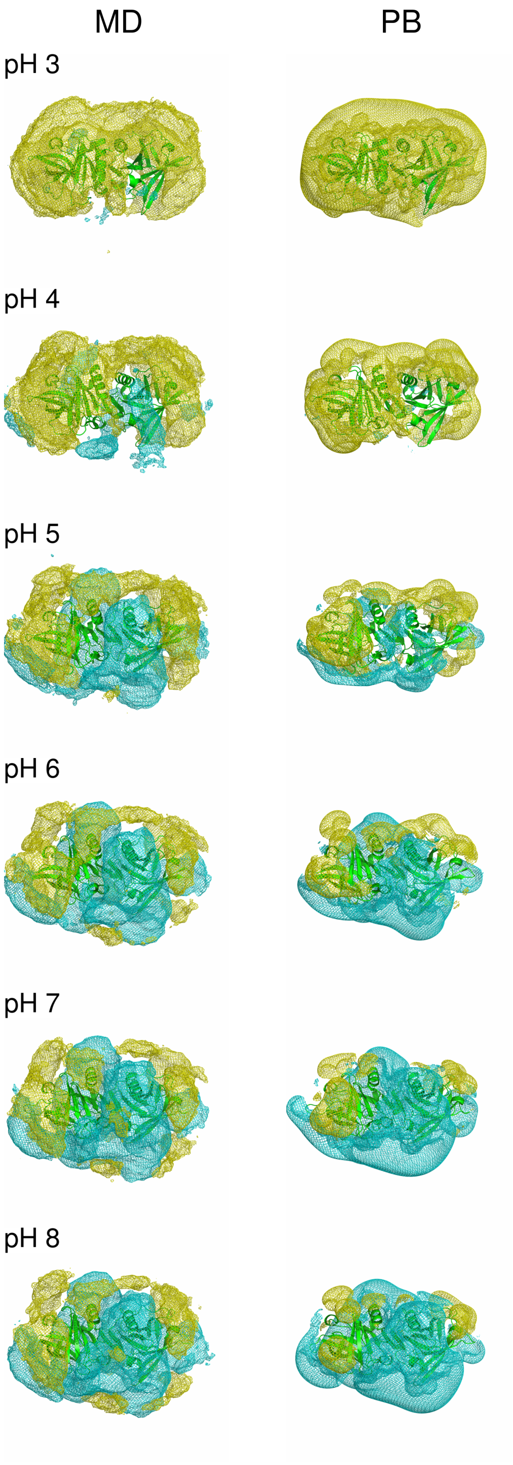

Figure S14: Density contours of 200 mM for the Na^+ (cyan) and Cl^- (yellow) ions in the dimer simulations, at different pH values, computed from either the MD simulations (**left**) or the PB model (**right**).

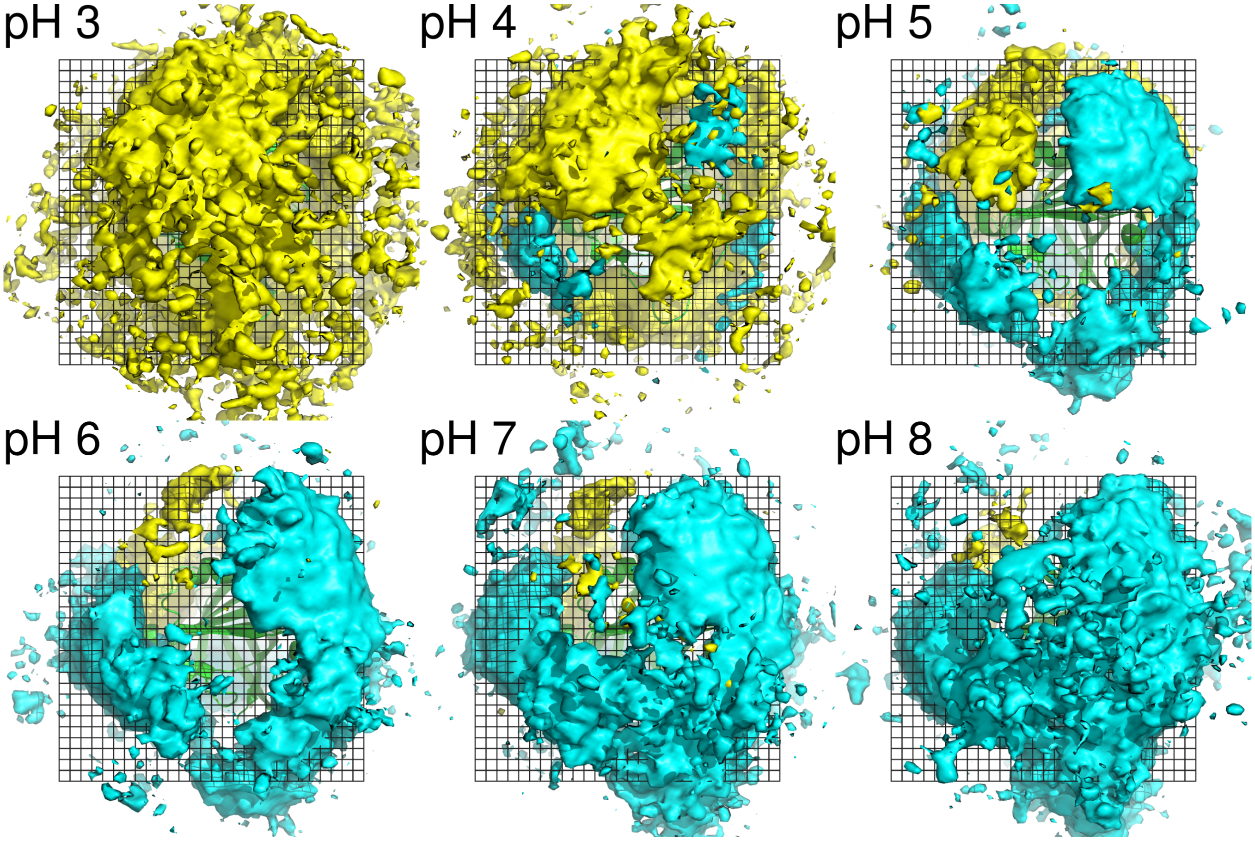

Figure S15: Density contours of 150 mM for the Na^+ (cyan) and Cl^- (yellow) ions in the monomer simulations, oriented to show the monomer face that would be found at the interface of the dimer, with the plane between both partners represented as a grid.

3.6 Dimer Configurations

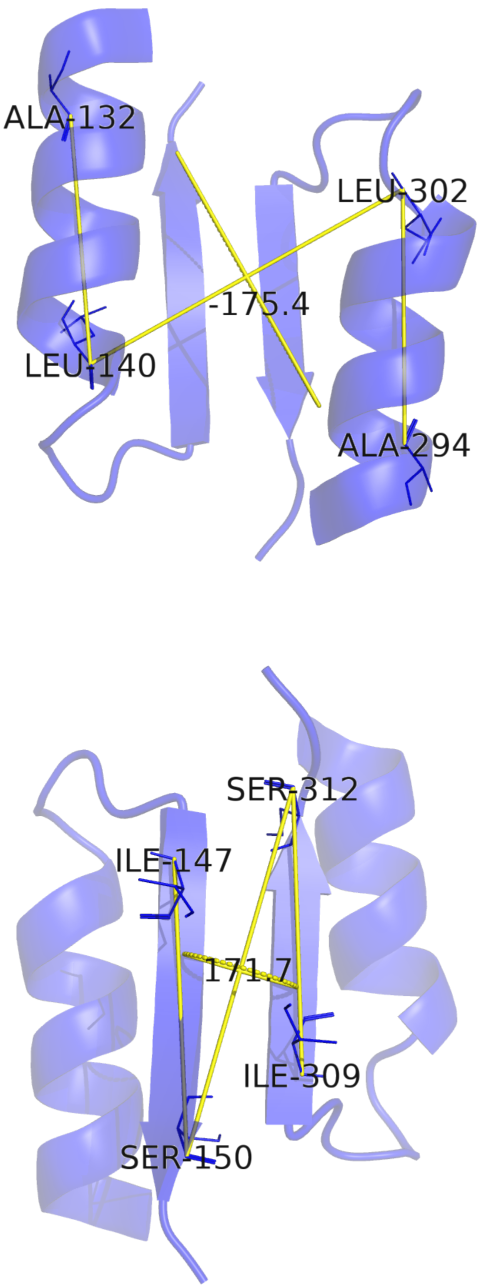

Figure S16: Dihedral angles between the helices (**top**) and β-strands (**bottom**) at the interface, whose average values as a function of pH are shown in Figure 12 in the main article. The dihedral between helices is defined with the C atoms of Ala132, Leu140, Leu302 and Ala294, in this order. The dihedral between β-strands is defined with atoms N of Ile147, C of Ser150, C of Ser312 and N of Ile309, in this order.

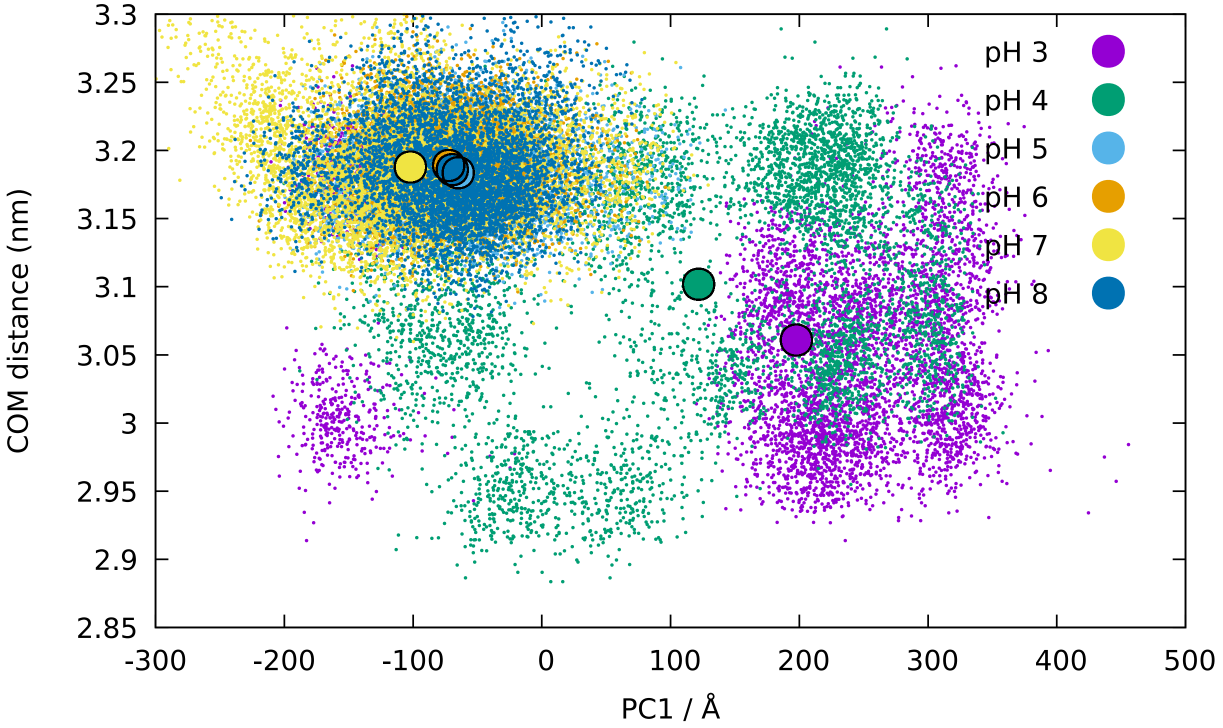

Figure S17: Scatter plot of the distance between the centers of mass of the dimer partners versus the first PC of the dimer configuration PCA. The large circles represent the averages at each pH value.

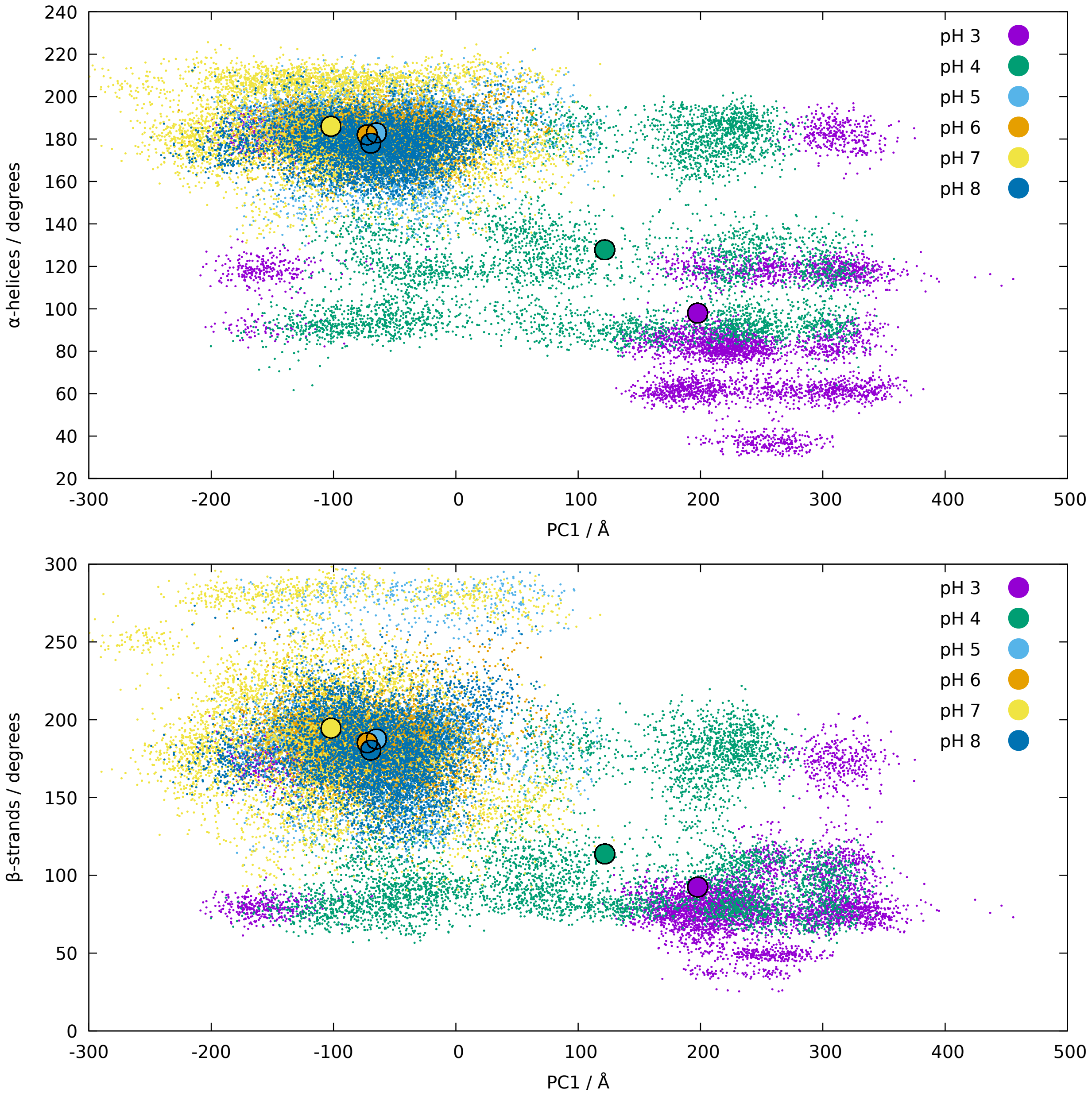

Figure S18: Scatter plots of the dihedral angles between the helices (**top**) and β-strands (**bottom**) at the dimer interface versus the first PC of the dimer configuration PCA. The large circles represent the averages at each pH value.

3.7 EF Loop

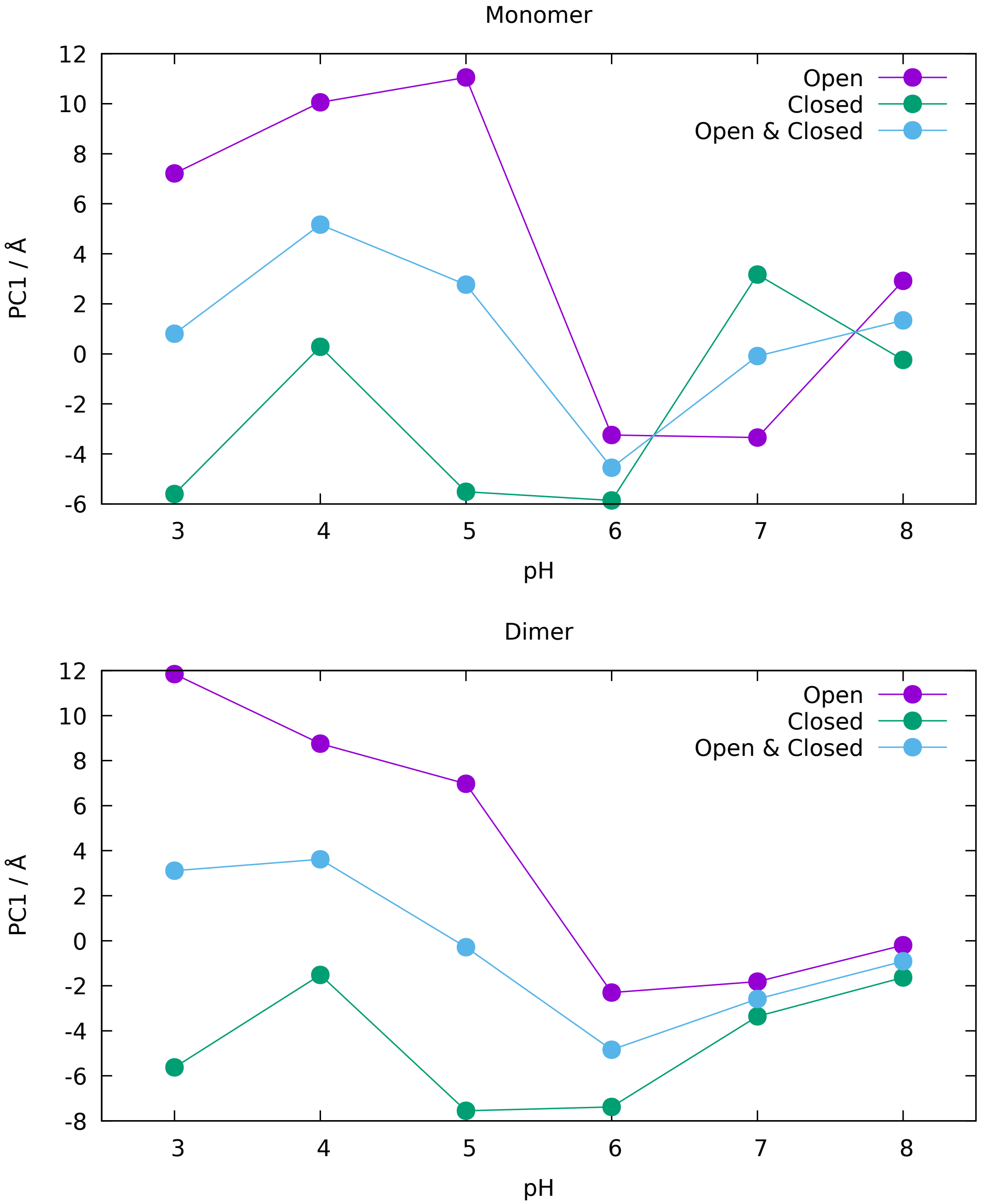

Figure S19: Average values of the first PC of the EF loop PCA, at different pH values. Averages were calculated for the simulations starting with an open conformation (purple), a closed conformation (green), and these two sets together (blue).

References

(S1) Mercadante, D.; Melton, L. D.; Norris, G. E.; Loo, T. S.; Williams, M. A.; Dobson, R. C.; Jameson, G. B. Bovine β-lactoglobulin is dimeric under imitative physiological conditions: dissociation equilibrium and rate constants over the pH range of 2.5–7.5. *Biophys. J.* **2012**, *103*, 303–312.

(S2) McKenzie, H.; Sawyer, W. Effect of pH on β-Lactoglobulins. *Nature* **1967**, *214*, 1101–1104.

(S3) Sakurai, K.; Oobatake, M.; Goto, Y. Salt-dependent monomer–dimer equilibrium of bovine β-lactoglobulin at pH 3. *Protein Sci.* **2001**, *10*, 2325–2335.

(S4) Bello, M.; Pérez-Hernández, G.; Fernández-Velasco, D. A.; Arreguín-Espinosa, R.; García-Hernández, E. Energetics of protein homodimerization: effects of water sequestering on the formation of β-lactoglobulin dimer. *Proteins: Struct., Funct., Bioinf.* **2008**, *70*, 1475–1487.

(S5) Timasheff, S. N.; Townend, R. Molecular Interactions in β-Lactoglobulin. V. The Association of the Genetic Species of β-Lactoglobulin below the Isoelectric Point. *J. Am. Chem. Soc.* **1961**, *83*, 464–469.

(S6) Knott, G. D. *Interpolating cubic splines*; Springer Science & Business Media: New York, 2000; Vol. 18.

(S7) Efron, B.; Tibshirani, R. J. *An Introduction to the Bootstrap*, 1st ed.; Chapman and Hall/CRC; Springer: Boca Raton, Florida, 1993.

(S8) Kabsch, W.; Sander, C. Dictionary of protein secondary structure: Pattern recognition of hydrogen-bonded and geometrical features. *Biopolymers* **1983**, *22*, 2577–2637.

(S9) Taylor, J. *An Introduction to Error Analysis: The Study of Uncertainties in Physical Measurements*; University Science Books: Sausalito, CA, 1997.

(S10) Ben-Naim, A. Y. *Statistical thermodynamics for chemists and biochemists*; Springer Science & Business Media: New York, 1992.

(S11) Tang, L.-H.; Adams Jr, E. Sedimentation equilibrium in reacting systems. VII. The temperature-dependent self-association of β-lactoglobulin A at pH 2.46. *Arch. Biochem. Biophys.* **1973**, *157*, 520–530.

(S12) Kelly, M. J.; Reithel, F. J. Thermodynamic analysis of the monomer–dimer association of β-lactoglobulin A at the isoelectric point. *Biochemistry* **1971**, *10*, 2639–2644.

(S13) Allen, M. P.; Tildesley, D. J. *Computer Simulation of Liquids*, 1st ed.; Oxford University Press: New York, 1987.
