# Supplementary material for: Correction to “Approach to Study pH-Dependent Protein Association Using Constant-pH Molecular Dynamics: Application to the Dimerization of β-Lactoglobulin”
Source: J Chem Theory Comput. 2022 Jun 29;18(7):4596–7. doi: 10.1021/acs.jctc.2c00605 (PMC9815691; doi:10.1021/acs.jctc.2c00605)
Supplement: Supplementary file 1 — ct2c00605_si_001.pdf [file ct2c00605_si_001.pdf]

Supporting Information: Approach to study pH-dependent protein association using constant-pH MD: application to the dimerization of β-lactoglobulin

Lucie da Rocha, António M. Baptista,* and Sara R. R. Campos*

Instituto de Tecnologia Química e Biológica António Xavier, Universidade Nova de Lisboa, Av. da República, 2780-157 Oeiras, Portugal

E-mail: baptista@itqb.unl.pt; scampos@itqb.unl.pt

Table S2: Average system net charge in the simulations with ions.

	net charge	
pH	monomer	dimer
3	0.07	0.46
4	0.04	−0.21
5	1.20	0.04
6	0.09	0.14
7	−0.10	−0.30
8	−0.35	−0.12

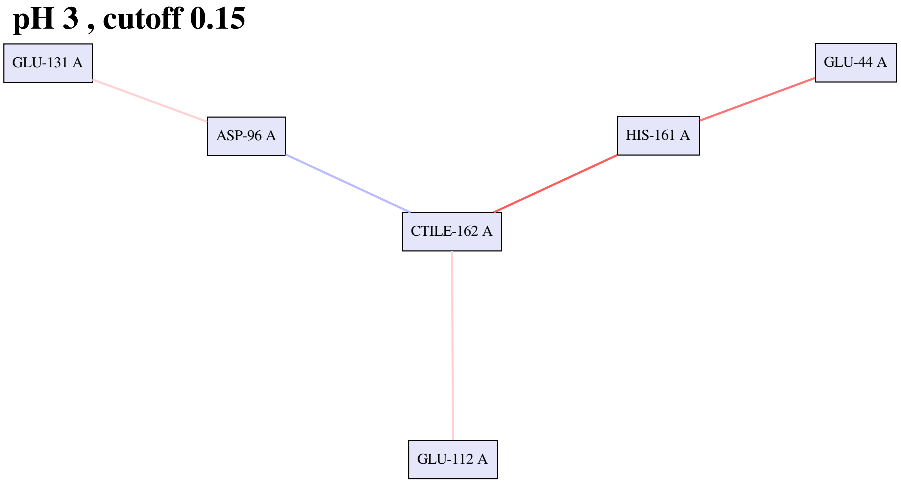

Figure S10: Networks of the protonation correlations in BLG **monomer** that are above 0.15 in absolute value. At pHs 7 and 8, no correlations above this cutoff are observed. Red/blue indicate negative/positive correlations and more intense colors correspond to higher absolute values. The strongest negative correlation is -0.38 (at pH 4) and the strongest positive correlation is 0.31 (at pH 6). Networks were drawn with the Graphviz software package (https://graphviz.org).

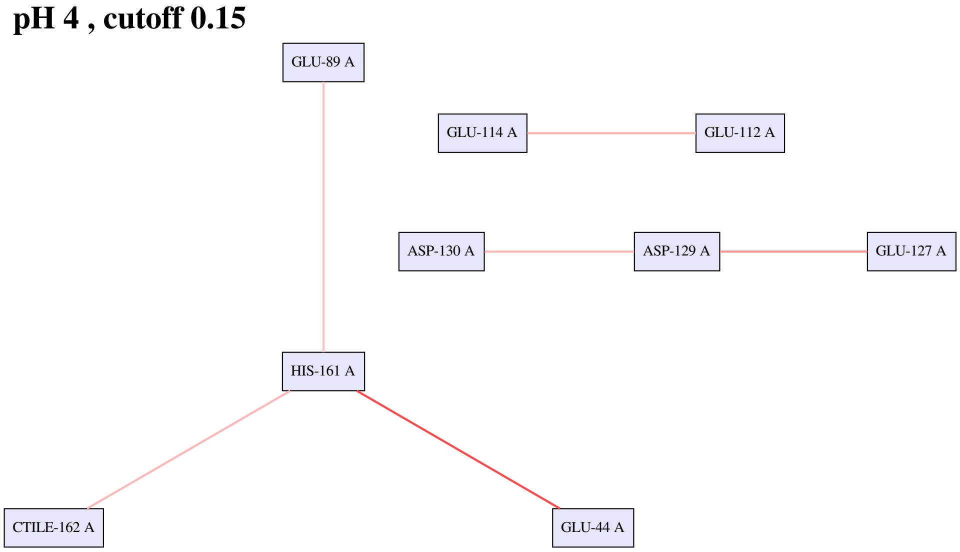

Figure S10: (continued, part 2)

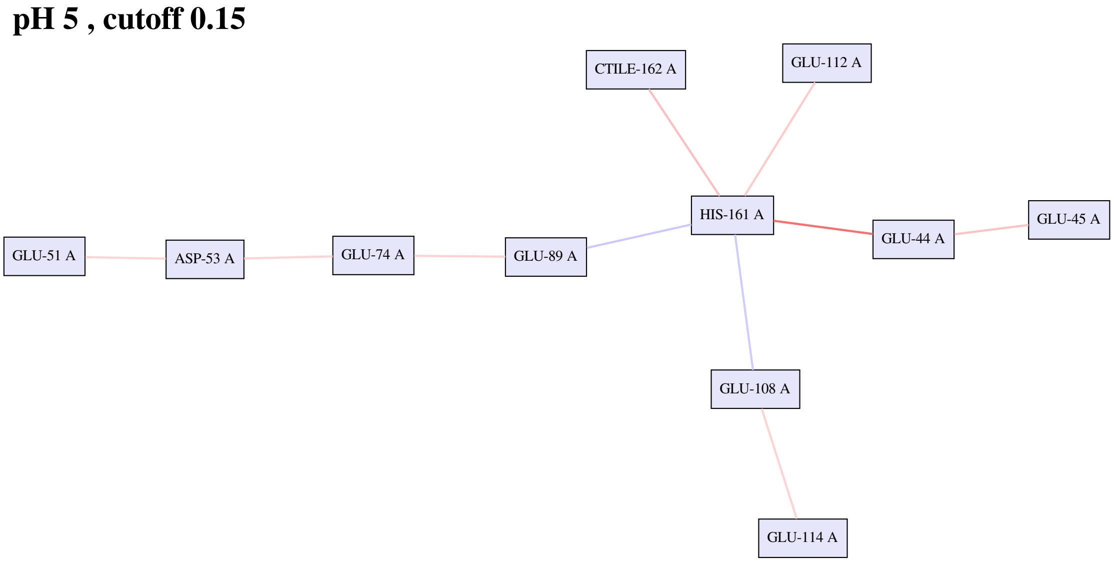

Figure S10: (continued, part 3)

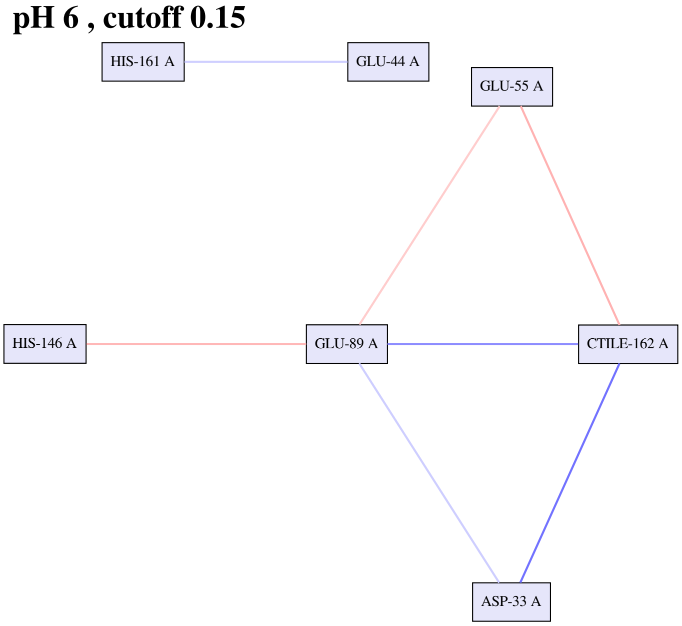

Figure S10: (continued, part 4)
